# Fine-tune control of targeted RNAi efficacy by plant artificial small RNAs

**DOI:** 10.1093/nar/gkaa343

**Published:** 2020-05-12

**Authors:** Lucio López-Dolz, Maria Spada, José-Antonio Daròs, Alberto Carbonell

**Affiliations:** Instituto de Biología Molecular y Celular de Plantas, Consejo Superior de Investigaciones Científicas-Universitat Politècnica de València, 46022 Valencia, Spain

## Abstract

Eukaryotic RNA interference (RNAi) results in gene silencing upon the sequence-specific degradation of target transcripts by complementary small RNAs (sRNAs). In plants, RNAi-based tools have been optimized for high efficacy and high specificity, and are extensively used in gene function studies and for crop improvement. However, efficient methods for finely adjusting the degree of induced silencing are missing. Here, we present two different strategies based on artificial sRNAs for fine-tuning targeted RNAi efficacy in plants. First, the degree of silencing induced by synthetic-trans-acting small interfering RNAs (syn-tasiRNAs) can be adjusted by modifying the precursor position from which the syn-tasiRNA is expressed. The accumulation and efficacy of Arabidopsis *TAS1c*-based syn-tasiRNAs progressively decrease as the syn-tasiRNA is expressed from positions more distal to the trigger miR173 target site. And second, syn-tasiRNA activity can also be tweaked by modifying the degree of base-pairing between the 3′ end of the syn-tasiRNA and the 5′ end of the target RNA. Both strategies were used to finely modulate the degree of silencing of endogenous and exogenous target genes in *Arabidopsis thaliana* and *Nicotiana benthamiana*. New high-throughput syn-tasiRNA vectors were developed and functionally analyzed, and should facilitate the precise control of gene expression in multiple plant species.

## INTRODUCTION

In most eukaryotes, gene expression can be suppressed through the sequence-specific degradation of target RNA by complementary small RNAs (sRNAs), a process termed RNA interference (RNAi). RNAi pathways are initiated by double-stranded RNA (dsRNA) that is recognized and processed by Dicer ribonucleases into sRNA duplexes (1,2). Typically, one of the strands of the duplex is loaded into an ARGONAUTE (AGO) protein, resulting in an RNA-induced silencing complex (RISC) that recognizes and silences complementary target RNA by diverse mechanisms (3,4). Since the initial observation in *Caenorabtitis elegans* that RNAi can be artificially triggered by exogenous dsRNA (2), targeted RNAi tools were developed and used in multiple organisms to study gene function, in medical therapies and for biotechnological purposes. Because RNAi does not completely abolish the expression of the target gene, major efforts while developing ‘knock-down’ RNAi tools have focused on maximizing RNAi efficacy to achieve high levels of target gene silencing (5).

In plants, early RNAi-based strategies also focused on maximal gene silencing typically by overexpressing dsRNA-generating transgenes with strong constitutive promoters to produce large populations of transgene-derived sRNAs. Despite their popularity, these approaches lack of specificity, as the accidental targeting of cellular transcripts sharing high-sequence complementarity with certain transgene-derived sRNAs is frequent (6). More recently, ‘second-generation RNAi’ strategies based on artificial sRNAs (art-sRNAs), such as artificial microRNAs (amiRNAs) and artificial/synthetic trans-acting small interfering RNA (atasiRNA/syn-tasiRNA, hereafter syn-tasiRNA), have overcome the limited specificity of initial RNAi strategies and are extensively used for highly specific gene silencing in plants (7–9). Art-sRNAs are typically 21-nt sRNAs computationally designed to be both highly effective and highly specific (with no predicted off-targets) (10). Regarding effectiveness, the art-sRNA is required to accumulate *in vivo* to high levels, and to present high sequence complementarity with the target RNA, as observed for endogenous sRNAs (11–13). In particular, a systematic study on miRNA–target RNA sequence complementarity requirements reported that miRNA targeting was unaffected, diminished or completely abolished when 1–3, 4–5 and >6 mismatches, respectively, were present at the 5′ end of the target site, while mismatches to the miRNA 5′ end strongly reduced miRNA efficacy (11). It is also known that mismatches within the sRNA ‘seed’ region (nts 2–13) have a drastic effect on sRNA efficacy, while mismatches at positions 1 or 14–21 have a more moderate effect (12,13). Regarding specificity, preferred art-sRNA web tools such as ‘Web MicroRNA Designer 3’ (WMD3) (7) and the ‘Plant Small RNA Maker Suite’ (P-SAMS) (14) include target specificity modules that use annotated plant transcriptomes to analyze all possible base-pairing interactions between the candidate sRNA and the complete set of cellular transcripts of a given species.

Syn-tasiRNAs possess a unique multiplexing capability that allows the expression of several syn-tasiRNA species from a single precursor. They are produced *in planta* by expressing a functional *TAS* precursor in which endogenous tasiRNA sequence(s) are replaced by syn-tasiRNA sequence(s) (9,15). Syn-tasiRNA biogenesis follows endogenous tasiRNA-generating pathways and is triggered by the cleavage of the syn-tasiRNA precursor by a miRNA/AGO complex. Next, one of the cleavage products is converted to dsRNA by RNA-dependent RNA polymerase 6 (RDR6), and sequentially processed by DCL4 into syn-tasiRNA duplexes in register with the trigger miRNA target site. The guide strand of the syn-tasiRNA duplex is loaded in an AGO protein, usually AGO1, to target and silence complementary RNAs. Syn-tasiRNAs have been generated from *Arabidopsis thaliana* (Arabidopsis) *TAS1a* (*AtTAS1a*), *TAS1c* (*AtTAS1*c) and *TAS3a* (*AtTAS3a*) precursors (16). In the case of *AtTAS1c-*based syn-tasiRNAs, the initial cleavage is mediated by miR173–AGO1 complexes, RDR6-dependent dsRNA is produced from the 3′ cleaved products, and the first DCL4 processing position in *AtTAS1c*, named 3′D1[+], cannot be used for syn-tasiRNA expression as it must contain the sequence of the 3’ half of miR173 target site (17). Syn-tasiRNAs have been mostly used to study tasiRNA biogenesis (17–23). More recently, their unique multiplexing capability combined with the availability of high-throughput methodologies to design and generate syn-tasiRNA constructs (14,24), has boosted their use and syn-tasiRNAs are currently considered a promising tool for crop improvement (9), particularly for enhanced antiviral resistance (25–27).

Despite the diversity of plant RNAi-based tools, efficient approaches for finely regulating the degree of induced silencing are missing. The possibility of obtaining the desired degree of silencing of a particular target gene is attractive in multiple ways, particularly in the study of genes whose complete repression results lethal or in the obtention of an allelic series of individuals displaying a gradient of phenotypes. Here, we describe two different strategies based on art-sRNAs for the fine-tune control of plant gene expression. First, we report that the accumulation and efficacy of *AtTAS1c*-based syn-tasiRNAs progressively decrease as the syn-tasiRNA is expressed from positions more distal to the miR173 target site. And second, we observed that syn-tasiRNA silencing activity can be gradually decreased by increasing the number of consecutive mismatches between the 3′ end of the art-sRNA and the 5′ end of the target RNA. Both strategies were used to modulate the silencing levels of endogenous genes in *A*.*thaliana*, and of exogenous viral RNAs infecting *Nicotiana benthamiana*. At last, a series of high-throughput syn-tasiRNA vectors for the fine-tune control of targeted RNAi in plants was generated and functionally analyzed.

## MATERIALS AND METHODS

### Plant materials and growth conditions

*Nicotiana benthamiana* plants were grown in a growth chamber at 25°C with a 12 h-light/12 h-dark photoperiod. *Arabidopsis thaliana* plants were grown in a growth chamber at 22°C with a 16 h-light/8 h-dark photoperiod. The floral dip method was used to genetically transform Arabidopsis plants with *Agrobacterium tumefaciens* GV3101 strain (28). T1 transgenic Arabidopsis were grown on plates including Murashige and Skoog medium and hygromycin (50 μg/ml) for 10 days before being transferred to soil. Plant photographs were taken with a Nikon D3000 digital camera with AF-S DX NIKKOR 18–55 mm f/3.5–5.6G VR lens.

### Plant phenotyping

All plant phenotypic analyses were conducted in blind. The flowering time of each independent line was determined by the number of days elapsed from seed plating to first bud opening (or ‘days to flowering). The ‘Ft’ phenotype was defined as a higher ‘days to flowering’ value when compared to the average ‘days to flowering’ value of the control set. A line was considered to have a ‘Trich’ phenotype when presenting a visually obvious higher number of trichomes in rosette leaves of 14 days old seedlings when compared to transformants of the *35S:syn-tasiR-GUS-D2&D3* control set. The ‘Ch42’ phenotype was scored in 10 days old seedling and was considered ‘weak’, ‘intermediate’ or ‘severe’ when seedlings had more than two leaves, exactly two leaves or no leaves at all (only two cotyledons), respectively.

### Artificial small RNA design

syn-tasiR-GUS_Ath_ and syn-tasiR-GUS_Nbe_ guide sequences were designed with P-SAMS webtool (14) using the full-length sequence of *Escherichia coli* β-glucuronidase gene (GenBank accession number S69414.1) and the off-targeting filtering in Arabidopsis TAIR10 transcriptome or *N. benthamiana* v.5.1 transcriptome (29), respectively. syn-tasiR-Su guide sequence was also designed with P-SAMS webtool using the full-length sequence of *N. benthamiana Su* gene (GenBank accession number AJ571699) and the off-targeting filtering in *N. benthamiana* v.5.1 transcriptome (29). In both guide sequence designs, off-targeting filter was enabled to avoid undesired off-target effects and increase specificity.

Twenty-one nucleotide guide sequences corresponding to syn-tasiR-CH42, syn-tasiR-GUS_Sly_, syn-tasiR-FT, syn-tasiR-Trich and syn-tasiR-TSWV were described previously (26,30–31).

Up to five consecutive mutations at the 3’end of syn-tasiR-FT or syn-tasiR-TSWV were introduced independently to induce 1–5 mismatches with FT or TSWV target RNAs, respectively. In each case, *TargetFinder* (13) script (https://github.com/carringtonlab/TargetFinder) was run to confirm that selected syn-tasiR-FT or syn-tasiR-TSWV variants do not target Arabidopsis TAIR10 or *N. benthamiana* transcriptome v5.1 (http://sefapps02.qut.edu.au/benWeb/subpages/downloads.php) (29), respectively.

### DNA constructs

*pENTR-AtTAS1c* plasmid (17) was polymerase chain reaction (PCR)-amplified with two divergent oligonucleotides, D2042 and D2043, to introduce two consecutive and inverted BsaI sites following position 3’D1[+] of *AtTAS1c*. Purified PCR product was ligated to a DNA insert including the sequence of the *ccd*B gene to generate *pENTR-AtTAS1c-D2-B/c*. The same PCR product was also re-ligated, and the *AtTAS1c* cassette transferred by recombination into *pMDC32B*, a modified version of *pMDC32* (32) with mutated BsaI sites (24) and a *ccd*B cassette was inserted between the two BsaI sites to generate *pMDC32B-AtTAS1c-D2-B/c*. To generate *pMDC32B-AtTAS1c-D2-B/c-AtMIR173*, first the two BsaI sites included in *AtMIR173* were mutated by PCR with oligos AC-278 and AC-284 and using as a template the *pBSDE-AtMIR173* vector including a *AtMIR173* cassette with 2 × 35S and T-Nos promoter and terminator sequences, respectively. The resulting cassette was PCR-amplified with oligos AC-14 and AC-15, gel purified and assembled into EcoRI-digested and gel-purified *pMDC32B-AtTAS1c-D2-B/c* DNA in the presence of NEBuilder HiFi DNA Assembly Master Mix (New England Biolabs) to generate *pMDC32B-AtTAS1c-D2-B/c-AtMIR173*.

Syn-tasiRNA constructs *35S:syn-tasiR-GUS_Ath_-D2*, *35S:syn-tasiR-GUS_Ath_-D2&D3*, *35S:syn-tasiR-GUS_Nbe_-D2, 35S:syn-tasiR-GUS_Sly_-D2*, *35S:syn-tasiR-FT-D2-Trich-D3*, *35S:syn-tasiR-Trich-D2-FT-D3*, *35S:syn-tasiR-FT-D2*, *35S:syn-tasiR-FT-D3*, *35S:syn-tasiR-FT-D4*, *35S:syn-tasiR-FT-D5*, *35S:syn-tasiR-CH42-D2, 35S:syn-tasiR-CH42-D3, 35S:syn-tasiR-CH42-D4, 35S:syn-tasiR-CH42-D5, 35S:syn-tasiR-TSWV-D2*, *35S:syn-tasiR-TSWV-D3*, *35S:syn-tasiR-TSWV-D4*, *35S:syn-tasiR-TSWV-D5*, *35S:syn-tasiR-FT-D2–1M*, *35S:syn-tasiR-FT-D2–2M*, *35S:syn-tasiR-FT-D2–3M*, *35S:syn-tasiR-FT-D2–4M*, *35S:syn-tasiR-FT-D2–5M*, *35S:syn-tasiR-Su*, *35S:syn-tasiR-Su/MIR173*, *35S:syn-tasiR-TSWV-D2–2M*, *35S:syn-tasiR-TSWV-D2–3M*, *35S:syn-tasiR-TSWV-D2–4M* and *35S:syn-tasiR-TSWV-D2–5M*, were obtained by ligating annealed oligo pairs AC-82/AC-83, AC-213/AC-214, AC-333/AC-334, AC-211/AC-212, AC-98/AC-99, AC-100/AC-101, AC-86/AC-87, AC-114/AC-115, AC-88/AC-89, AC-90/AC-91, AC-92/93, AC-116/AC-117, AC-94/AC-95, AC-96/AC-97, AC-102/AC-103, AC-104/AC-105. AC-106/AC-107, AC-108/AC-109, AC-124/AC-125, AC-126/AC-127, AC-128/AC-129, AC-130/AC-131, AC-132/AC-133, AC-288/AC-289, AC-288/AC-289, AC-138/AC-139, AC-140/AC-141, AC-142/AC-143 and AC-144/AC-145, respectively, into *pMDC32B-AtTAS1c-D2-B/c* as described (24) (Supplementary Protocol S1).

*35S: GUS*, *35S:MIR173a*, *35S:syn-tasiR-FT-D3-Trich-D4* and *35S:syn-tasiR-Trich-D3-FT-D4* were reported previously (17,24).

All DNA oligonucleotides used for generating the constructs described above are listed in Supplementary Table S7. The sequences of all syn-tasiRNA precursors are listed in Supplementary Text S1. The sequences of the *AtTAS1c-D2-B/c*-based syn-tasiRNA vectors are listed in Supplementary Text S2. The following syn-tasiRNA vectors are available from Addgene at http://www.addgene.org/: *pENTR-AtTAS1c-D2-B/c* (Addgene plasmid 137883), *pMDC32B-AtTAS1c-D2-B/c* (Addgene plasmid 137884) and *pMDC32B-AtTAS1c-D2-B/c-AtMIR173* (Addgene plasmid 137885).

### Transient expression of constructs and virus infection assays

Agroinfiltration of constructs in *N. benthamiana* leaves was done as described (33,34), using *A. tumefaciens* GV3101 strain. Virus infection assays with TSWV LL-N.05 isolate (35) were done as previously described (31,36).

### Small RNA gel blot assays

Total RNA from *A. thaliana* inflorescences or *N. benthamiana* leaves was isolated using TRIzol reagent (Thermo Fisher Scientific) followed by chloroform extraction and isopropanol precipitation as described (34). RNA gel blot assays including RNA separation in 17% polyacrylamide gels containing 0.5 × Tris/Borate/EDTA (TBE) and 7 M urea, RNA transfer to positively charged nylon membrane and probe synthesis using [γ-^32^P]ATP (PerkinElmer) and T4 polynucleotide kinase (Thermo Fisher Scientific) were done as described (17,24). A Typhoon Trio Variable Mode Imager System (Amersham Biosciences) was used to produce digital images from radioactive membranes. The ImageQuant version 5.2 software (Molecular Dynamics) was used for band quantification of band densities. DNA oligonucleotides used as probe for small RNA blots are listed in Supplementary Table S7.

### Real-time RT-qPCR

Real-time RT-qPCR was done using the same RNA samples that were used for sRNA-blot analysis, essentially as described (24). Briefly, two micrograms of DNaseI-treated total RNA served to produce first-strand complementary DNA using the SuperScript III system (Life Technologies). RT-qPCR was done on optical 96-well plates in the 7500 Fast Real-Time PCR System (Applied Biosystems) using the following program: 20 s at 95°C, followed by 40 cycles of 95°C for 3 s and 60°C for 30 s, with an additional melt curve stage consisting of 15 s at 95°C, 1 min at 60°C and 15 s at 95°C. The 20-μl reaction mixture contained 10 μl of 2× Fast SYBR Green Master Mix (Applied Biosystems), 2 ml of diluted complementary DNA (1:5) and 300 nM of each gene-specific primer. Primers used for RT-qPCR are listed in Supplementary Table S7. Target mRNA expression levels were calculated relative to four Arabidopsis reference genes (*ACT2*, *CBP20*, *SAND* and *UBQ10*) using the delta delta cycle threshold comparative method (Applied Biosystems) of the 7500 Fast Software (version 2.2.2; Applied Biosystems). Three independent biological replicates, and two technical replicates for each biological replicate were analyzed.

### DAS-ELISA assays

TSWV accumulation in extracts from apical *N. benthamiana* leaves collected at 10 or 20 dpi were analyzed by double antibody sandwich enzyme-linked immunosorbent assay (DAS-ELISA) using the TSWV Complete kit (Bioreba) as described (31). Samples were considered to be infected (DAS-ELISA-positive) when absorbance was higher than three times the average absorbance of the samples from mock-inoculated controls. The absorbance values were used as an indirect estimate of the viral accumulation as reported previously (37).

### Chlorophyll extraction and analysis

Pigments from *N. benthamiana* infiltrated leaf tissues (40 mg of fresh weight) were extracted with 5 ml of 80% (v/v) acetone as described (38). Absorbance was measured at 663 and 647 nm in a Multiskan GO microplate reader (Thermo Scientific, USA) using the SkanIt Software v.3.2 (Thermo Scientific). Content in chlorophyll *a* was calculated with the formula: chlorophyll a (mg/l in extract) = 12.21 * Absorbance_663 nm_ – 2.81 * Absorbance_647 nm_.

### Gene and virus identifiers

Arabidopsis gene identifiers are as follows: *ACT2* (AT3G18780), *CBP20* (AT5G44200), *CH42* (AT4G18480), *CPC* (AT2G46410), *ETC2* (AT2G30420), *FT* (AT1G65480), *SAND* (AT2G28390), *TRY* (AT5G53200) and *UBQ10* (AT4G05320). *Nicotiana benthamiana Su* gene identifier is AJ571699. TSWV LL-N.05 segment L, M and S genome identifiers are KP008128, FM163373 and KP008129, respectively.

## RESULTS

### Enhanced silencing by *AtTAS1c*-based syn-tasiRNAs expressed from positions proximal to the miR173 target site

After a careful examination of our previous work, we noticed that two different syn-tasiRNAs, syn-tasiR-FT and syn-tasiR-Trich targeting *FLOWERING LOCUS T* (*FT*) and three *MYB* transcripts (*TRIPTYCHON* [*TRY*], *CAPRICE* [*CPC*] and *ENHANCER OF TRIPTYCHON AND CAPRICE2* [*ETC2*]), respectively, accumulated to higher levels and induced stronger target silencing in Arabidopsis when expressed from DCL4-processing position 3’D3[+] compared to when expressed from position 3’D4[+] of *AtTAS1c* precursors (24). This observation, not reported at that time, led us to hypothesize that *AtTAS1c*-based syn-tasiRNA accumulation and silencing activity may depend on the position of the precursor from where syn-tasiRNAs are expressed.

**Figure 1.**
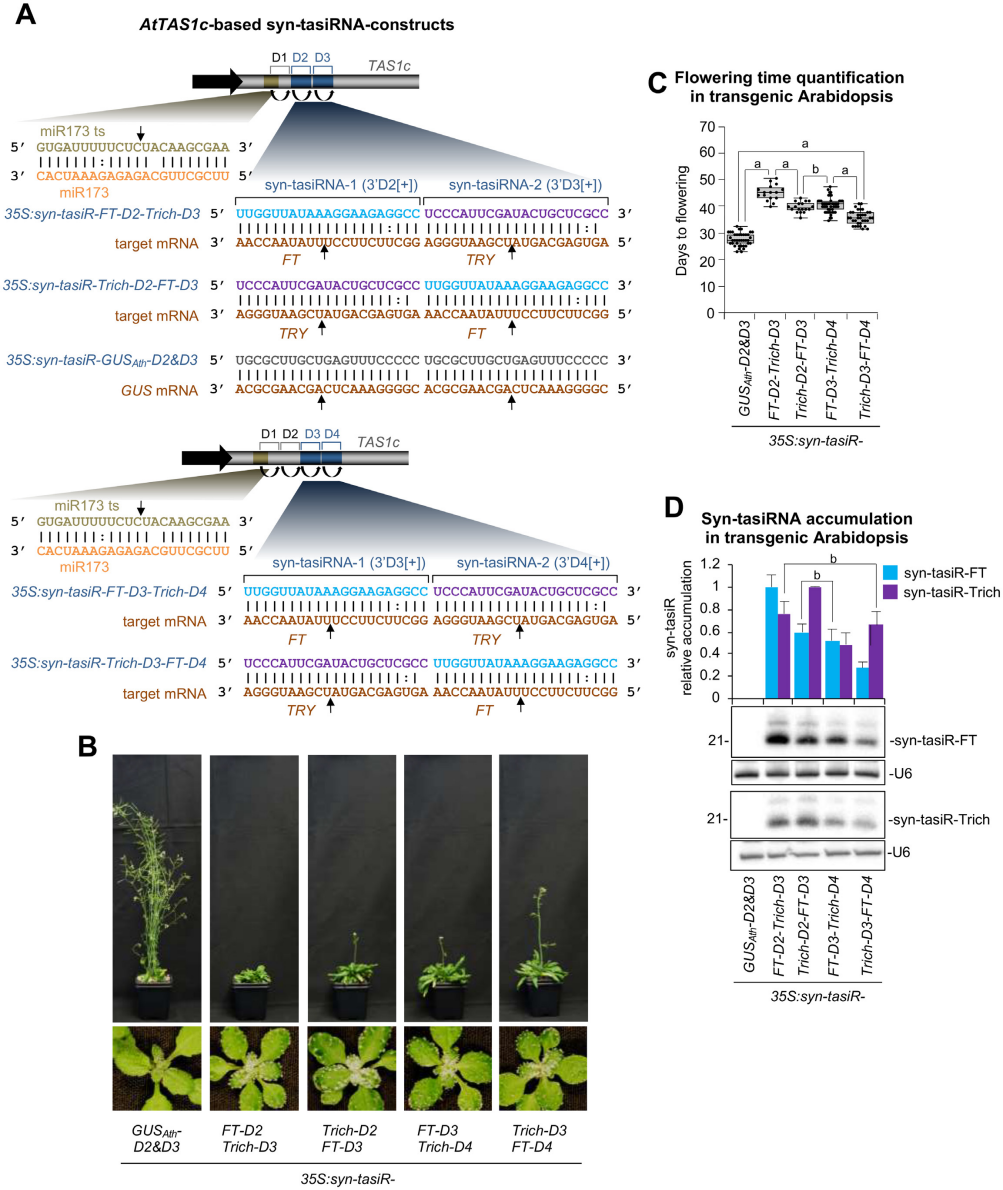
Comparative analyses of *AtTAS1c*-based syn-tasiRNAs expressed from distinct dual configurations in Arabidopsis T1 transgenic plants. (**A**) Organization of syn-tasiRNA constructs. tasiRNA positions 3’D1[+] to 3’D3[+] or 3’D4[+] are indicated by brackets, with positions 3’D2[+]/3’D3[+] and 3’D3[+]/3’D4[+] highlighted in blue in the upper and lower panels, respectively. Curved black arrows indicate DCL4 processing sites. Black linear arrows indicate sRNA-guided cleavage sites. ts refers to target site. (**B**) Representative images of Arabidopsis plants expressing unique syn-tasiRNA constructs. (**C**) Box plot representing the mean flowering time (days to flowering) of Arabidopsis plants expressing syn-tasiRNA constructs. Letter ‘a’ Pairwise Student's *t*-test comparisons are represented with a black line including the letter ‘a’ if significantly different (*P* < 0.05) and the letter ‘b’ if not (*P* > 0.05). (**D**) Northern blot detection of syn-tasiRNAs in Arabidopsis plants. The graph at top shows mean (*n* = 3) relative syn-tasiR-FT (blue) and syn-tasiR-Trich (purple) levels + standard deviation (*35S:syn-tasiR-FT-D2-Trich-D3* and *35S:syn-tasiR-Trich-D2-FT-D3* lanes = 1.0 for syn-tasiR-FT and syn-tasiR-Trich, respectively). One blot from three biological replicates is shown. Each biological replicate is a pool of at least nine independent lines that were randomly selected. The U6 RNA blot is shown as a loading control. Other details are as in C.

To test this hypothesis, we used the Arabidopsis/syn-tasiR-FT/syn-tasiR-Trich system in which non-overlapping silencing effects of syn-tasiR-FT and syn-tasiR-Trich correspond to a delay in flowering time and an increase in trichome number, respectively (24). In particular, we compared the accumulation and efficacy of syn-tasiR-FT and syn-tasiR-Trich when co-expressed in unique dual configurations from positions 3’D2[+] and 3’D3[+] (*35S:AtTA1c-FT-D2-Trich-D3* and *35S:AtTA1c-Trich-D2-FT-D3* constructs), or 3’D3[+] and 3’D4[+] (*35S:AtTA1c-FT-D3-Trich-D4* and *35S:AtTA1c-Trich-D3-FT-D4* constructs) (Figure 1A). For comparative purposes, the *35S:GUS_Ath_-D2&D3* construct aimed to express a control syn-tasiRNA (syn-tasiR-GUS_Ath_, without predicted off-targets in Arabidopsis) in single configuration and from positions 3’D2[+] and 3’D3[+] was also generated (Figure 1A). All these constructs were introduced independently into Arabidopsis Col-0 plants, and all the analyses were done in T1 transformants. Phenotypic analyses included the score of the day of flowering and the trichome number in rosette leaves for each transformant. Molecular analyses included the quantification of syn-tasiRNA accumulation in the different lines by RNA-blot assays.

Regarding the flowering time analysis, all transformants expressing dual configuration syn-tasiRNA constructs had a delay in flowering compared to *35S:GUS_Ath_-D2&D3* control transformants (Supplementary Table S1). In particular, *35S:syn-tasiR-FT-D2-Trich-D3* transformants expressing syn-tasiR-FT from position 3’D2[+] flowered on average 16 days later than controls (Figure 1B and C). Interestingly, this delay in flowering was progressively reduced in transformants expressing syn-tasiR-FT from positions 3’D3[+] and 3’D4[+] (Figure 1B and C). Regarding the trichome number analysis, 82, 76, 71 and 61% of the transformants expressing the dual-configuration syn-tasiRNA constructs *35S:AtTA1c-Trich-D2-FT-D3, 35S:AtTA1c-Trich-D3-FT-D4*, *35S:AtTA1c-FT-D2-Trich-D3* and *35S:AtTA1c-FT-D3-Trich-D4* had increased number of trichomes in rosette leaves, respectively (Figure 1B and Supplementary Table S1).

Next, the accumulation of syn-tasiR-FT and syn-tasiR-Trich in transformants was examined (Figure 1D). In all cases, syn-tasiRNAs accumulated to detectable levels and as a single band at 21 nucleotide (nt), suggesting an accurate processing of *AtTAS1c*-based precursors (Figure 1D). Importantly, both syn-tasiRNAs accumulated at higher levels when expressed from position 3’D2[+], and their accumulation was progressively reduced when expressed from positions more distal to the miR173 target site (Figure 1D). Taken together these results indicate that syn-tasiRNA accumulation and efficacy are higher when expressed from positions proximal to the miR173 target site in *AtTAS1c* precursors. In particular, we observed that syn-tasiRNAs expressed from position 3’D2[+] accumulate to higher levels and induce stronger target silencing.

### New high-throughput vectors for expressing syn-tasiRNAs downstream position 3’D1[+] of *AtTAS1c*

**Figure 2.**
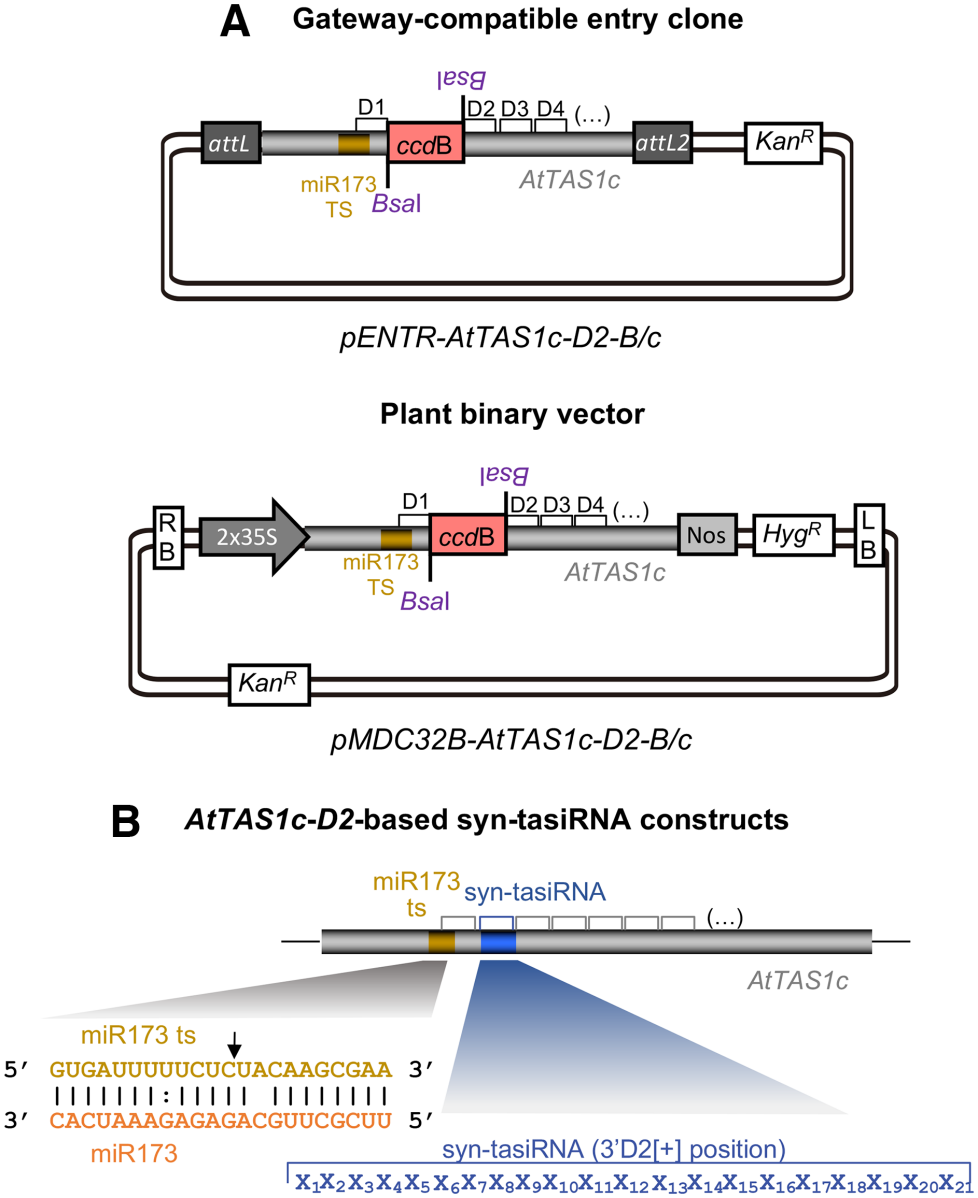
*AtTAS1c-D2-B/c*-based vectors for direct cloning of syn-tasiRNAs downstream position 3′D1[+]. (**A**) Diagrams of *AtTAS1c-D2-B/c*-based vectors. Top, diagram of the Gateway-compatible *pENTR-AtTAS1c-D2-B/c* entry vector. Bottom, diagram of the *pMDC32B-AtTAS1c-D2-B/c* binary vector for *in planta* expression of syn-tasiRNAs. RB: right border; 35S: *Cauliflower mosaic virus* promoter; BsaI: BsaI recognition site, *ccd*B: gene encoding the gyrase toxin; LB: left border; attL1 and attL2: GATEWAY recombination sites. *Kan^R^*: kanamycin resistance gene; *Hyg^R^*: hygromycin resistance gene. (**B**) Organization of *AtTAS1c-D2*-based syn-tasiRNA constructs. In the example diagram, one single 21-nt guide syn-tasiRNA sequence was introduced at the 3’D2[+] position in *AtTAS1c*. Other details are as in Figure 1A.

Previous high-throughput *AtTAS1c*-based syn-tasiRNA ‘B/c’ vectors (24) allowed syn-tasiRNA cloning and expression downstream position 3′D2[+]. Here, we developed two additional ‘B/c’ vectors for the efficient cloning and expression of syn-tasiRNAs downstream position 3′D1[+] (Figure 2A). *pENTR-AtTAS1c-D2-B/c* Gateway-compatible entry vector was generated for direct cloning of syn-tasiRNA inserts and subsequent recombination into the preferred Gateway expression vector including the promoter, terminator or other features of choice. *pMDC32B-AtTAS1c-D2-B/c* vector was generated for the direct cloning of syn-tasiRNA inserts into a binary expression vector, thus avoiding the need for subcloning the syn-tasiRNA cassette from an intermediate plasmid. Both vectors contain a truncated *AtTAS1c* precursor sequence including a 1461-bp DNA cassette with the control of cell death (*ccd*B) gene (39) flanked by two inverted BsaI sites downstream position 3’D1[+] (Figure 2A). The generation of a syn-tasiRNA construct is similar to that described for previous *AtTAS1c-B/c*-based syn-tasiRNA vectors (24). Briefly, the syn-tasiRNA insert is generated by annealing two 46-nt-long overlapping and partially complementary oligonucleotides containing the syn-tasiRNA sequence(s) and with 5’-TTTA and 5’-CCGA overhangs, and ligated directionally into the BsaI-digested *AtTA1c-D2-B/c*-based vector in a simple, fast and cost effective manner (Supplementary Figure S1 and Protocol S1). The configuration of *AtTAS1c-D2*-based syn-tasiRNA constructs expressing one single syn-tasiRNA is shown in Figure 2B. These vectors were used to more systematically test the effect of syn-tasiRNA precursor position on gene silencing efficacy, as described next.

### Fine-tuning silencing of Arabidopsis endogenous transcripts with syn-tasiRNAs expressed from different *AtTAS1c* positions

To more systematically study the effect of the precursor position, we reasoned that both syn-tasiRNA accumulation and silencing activity should be examined when expressing the same syn-tasiRNA sequence in single configuration from distinct positions of *AtTAS1c* precursors. The activity of syn-tasiRNAs was first examined in Arabidopsis by analyzing their effects in silencing *FT* or *CHLORINA42* [*CH42*] endogenous transcripts.

**Figure 3.**
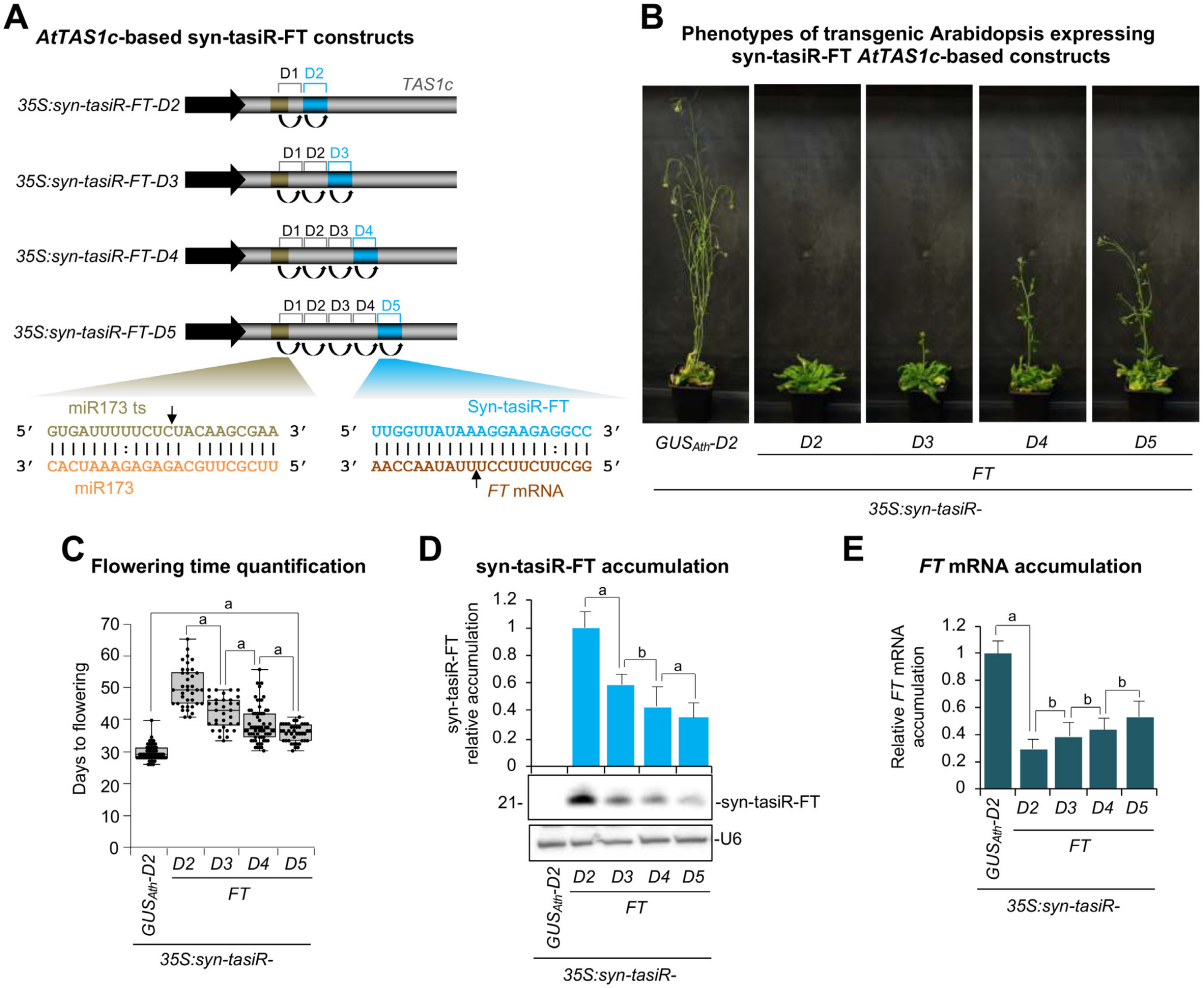
Functional analysis of *AtTAS1c-*based syn-tasiRNA constructs expressing a single syn-tasiRNA against Arabidopsis *FT* (syn-tasiR-FT) from different precursor positions. (**A**) Diagram of the syn-tasiRNA constructs. Positions including syn-tasiR-FT are highlighted in blue. Other details are as in Figure 1A. (**B**) Representative images of Arabidopsis T1 transgenic plants expressing different syn-tasiRNA constructs. (**C**) Box plot representing the mean flowering time of Arabidopsis T1 transgenic plants expressing syn-tasiR-FT from different constructs. Other details are as in Figure 1C. (**D**) Northern blot detection of syn-tasiR-FT in Arabidopsis plants. The graph at top shows mean (*n* = 3) relative syn-tasiR-FT (light blue) levels + standard deviation (*35S:syn-tasiR-FT-D2* = 1.0). Other details are as in Figure 1C and D. (**E**) Accumulation of *FT* mRNA in Arabidopsis plants. Mean relative level + standard error (dark blue) of Arabidopsis FT mRNAs after normalization to *ACTIN2* (*ACT2*, *CAP-BINDING PROTEIN20* (*CBP20*), *SAND* and *POLYUBIQUITIN10* (*UBQ10*), as determined by quantitative RT-PCR (RT-qPCR) (*35S:syn-tasiR-GUS_Ath_-D2* = 1–0 in all comparisons). Other details are as in Figure 1C.

**Figure 4.**
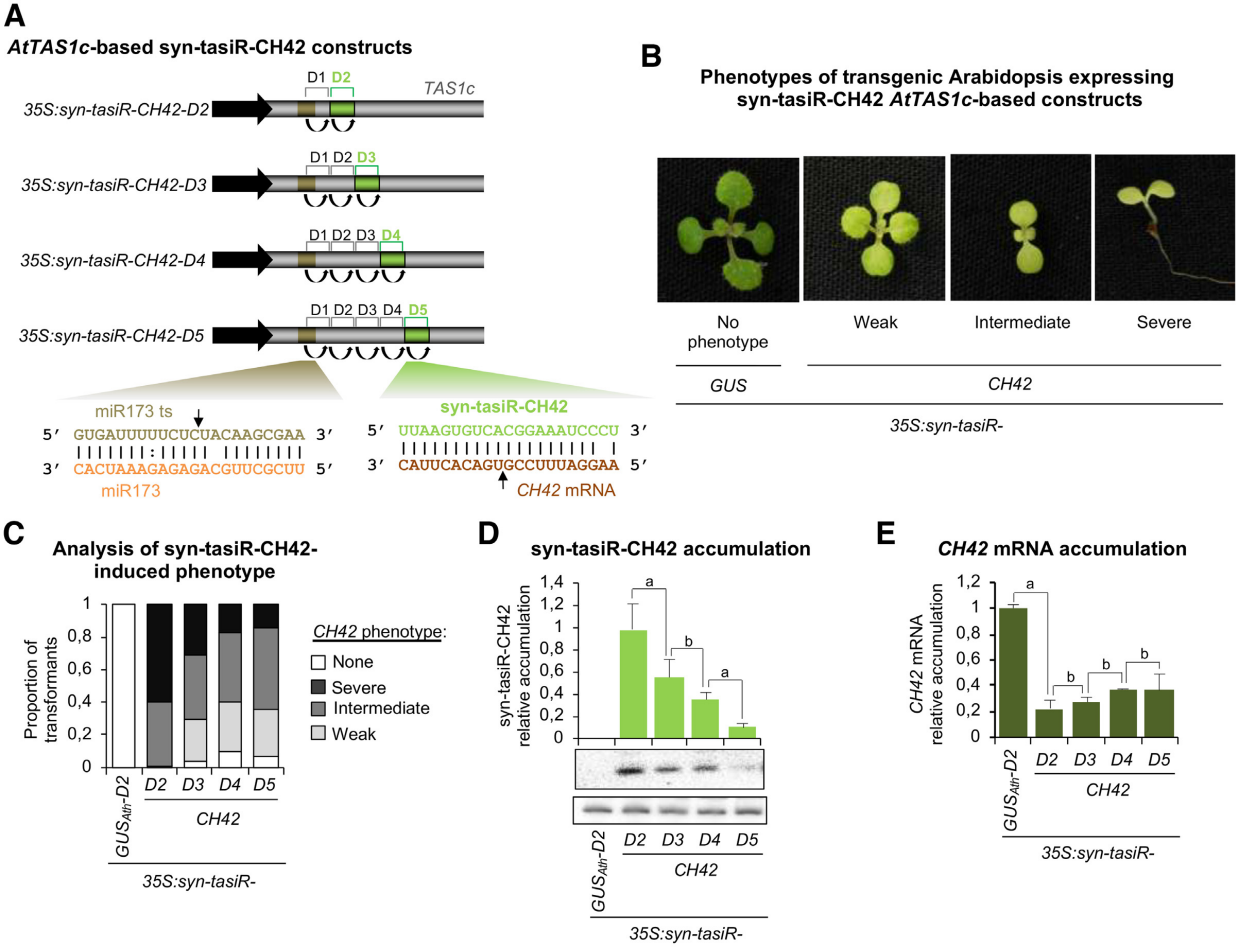
Functional analysis of *AtTAS1c-*based syn-tasiRNA constructs expressing a single syn-tasiRNA against Arabidopsis *CH42* (syn-tasiR-CH42) from different precursor positions. (**A**) Diagram of the syn-tasiRNA constructs. Positions including syn-tasiR-CH42 are highlighted in green. Other details are as in Figure 1A. (**B**) Representative images of 10-day-old T1 seedlings expressing different syn-tasiRNA constructs and showing bleaching phenotypes of diverse degree. (**C**) Bar graph representing, for each line, the proportion of seedlings displaying a severe (black areas), intermediate (dark gray areas), or weak (light gray areas) bleaching phenotype, or with wild-type appearance (white areas). (**D**) Northern blot detection of syn-tasiR-CH42 in Arabidopsis seedlings. The graph at top shows mean (*n* = 3) relative syn-tasiR-CH42 (light green) levels + standard deviation (*35S:syn-tasiR-CH42-D2* = 1.0). Other details are as in Figure 1C and D. (**E**) Accumulation of *CH42* mRNA in Arabidopsis plants. Mean relative level + standard error (dark green) of Arabidopsis *CH42* mRNAs (*35S:syn-tasiR-GUS_Ath_-D2* = 1–0 in all comparisons). Other details are as in Figures 1C and 3E.

The *pMDC32B-AtTAS1c-D2-B/c* vector was used to generate a series of constructs in which the syn-tasiR-FT or syn-tasiR-CH42 sequences were inserted in single configuration either in position 3’D2[+] (*35S:syn-tasiR-FT-D2*, *35S:syn-tasiR-CH42-D2*), 3’D3[+] (*35S:syn-tasiR-FT-D3*, *35S:syn-tasiR-CH42-D3*), 3’D4[+] (*35S:syn-tasiR-FT-D4*, *35S:syn-tasiR-CH42-D4*) or 3’D5[+] (*35S:syn-tasiR-FT-D5*, *35S:syn-tasiR-CH42-D5*) (Figures 3A and 4A). A control construct (*35S:syn-tasiR-GUS_Ath_-D2*) expressing syn-tasiR-GUS_Ath_ from position 3’D2[+] was also generated. All constructs were introduced independently in Arabidopsis Col-0. Plant phenotypes, syn-tasiRNA accumulation and target mRNA accumulation were measured in Arabidopsis T1 transgenic lines.

All lines expressing syn-tasiR-FT flowered later than the average flowering time of the *35S:syn-tasiR-GUS_Ath_-D2* control lines (Supplementary Table S2). *35S:syn-tasiR-FT-D2* transformants expressing syn-tasiR-FT from 3’D2[+] had on average a 19-day delay in flowering compared to control lines (Figure 3B and C). Interestingly, this delay in flowering was progressively reduced when syn-tasiR-FT was expressed from positions 3’D3[+], 3’D4[+] and 3’D5[+] in *35S:syn-tasiR-FT-D3*, *35S:syn-tasiR-FT-D4* and *35S:syn-tasiR-FT-D5* transformants, respectively (Figure 3B and C). Syn-tasiR-FT accumulation analyzed by RNA-blot assays was higher when syn-tasiR-FT was expressed from position 3’D2[+], and progressively decreased when the syn-tasiRNA was expressed from more distal positions (Figure 3D). Conversely, target FT mRNA accumulation analyzed by RT-qPCR was lower when syn-tasiR-FT was expressed from position 3’D2[+], and progressively increased when the syn-tasiRNA was expressed from more distal positions (Figure 3E). Importantly, the silencing effects observed in T1 transgenic lines were essentially maintained in Arabidopsis T2 progeny (Supplementary Figure S3). One-hundred and five of 106 transgenic lines expressing *35S:syn-tasiR-CH42-D2* were smaller than controls and had pale or bleached leaves and cotyledons (Figure 4B and C; Supplementary Table S3), as expected due to deficient chlorophyll biosynthesis with a loss of Ch42 magnesium chelatase (22,40). Sixty-four of these plants had a severe bleached phenotype with absence of visible true leaves at 12 days after plating, while only 40, 30 and 9 plants expressing *35S:syn-tasiR-CH42-D3*, *35S:syn-tasiR-CH42-D4* and *35S:syn-tasiR-CH42-D5* had a similar severe phenotype, respectively (Figure 4B and C; Supplementary Table S3). A similar trend was observed in the number of lines of each construct showing intermediate or weak phenotypes (Figure 4B and C; Supplementary Table S3). The absence of syn-tasiRNA accumulation in lines with no phenotypes was confirmed by northern blot analysis (Supplementary Figure S2). All together, these results suggest that endogenous transcripts are more effectively silenced with syn-tasiRNAs expressed from positions proximal to the miR173 target site. They also suggest that the expression of endogenous genes can be finely regulated with syn-tasiRNAs derived from distinct precursor positions.

### Modulation of antiviral resistance in *Nicotiana benthamiana* with syn-tasiRNAs expressed from different *AtTAS1c* positions

Next, we aimed to test if the same strategy could also be applied to silence exogenous transcripts, such as viral RNAs. For that purpose, we used the *N. benthamiana*/*Tomato spotted wilt virus* (TSWV) pathosystem previously exploited for the identification of effective anti-TSWV art-sRNAs (31,36). In this system, an art-sRNA construct is infiltrated in one whole *N. benthamiana* leaf, which is then inoculated with TSWV 2 days later. The antiviral activity of the artificial sRNA is assessed (i) phenotypically, by monitoring the appearance of typical TSWV-induced symptoms in inoculated tissues (local lesions) and in distant non-inoculated tissues (leaf epinasty and chlorosis) during 20 days, and (ii) molecularly, by analyzing TSWV accumulation in non-inoculated tissues by enzyme-linked immunosorbent assay (ELISA) at two timepoints (10 and 20 days post-inoculation [dpi]).

**Figure 5.**
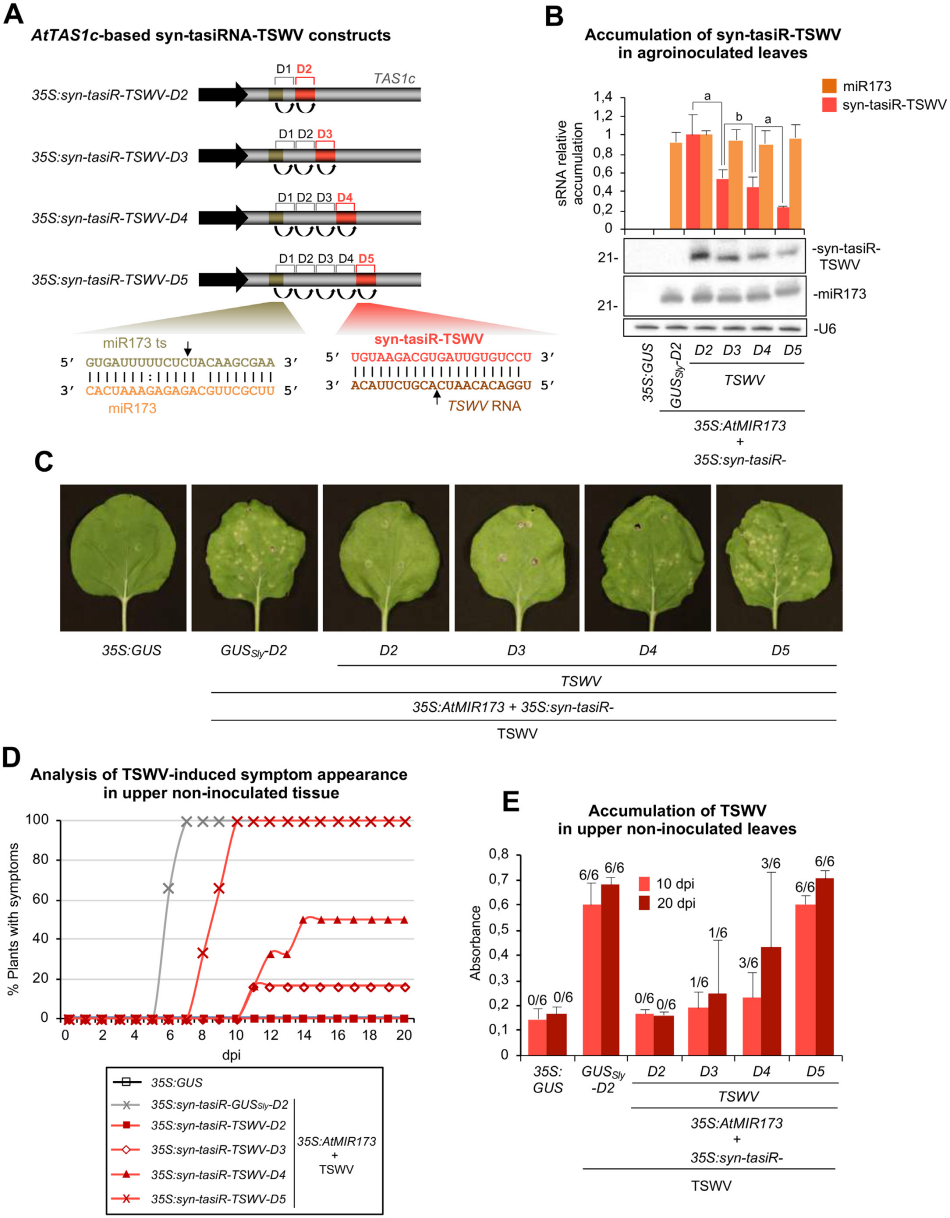
Functional analysis of *AtTAS1c-*based syn-tasiRNA constructs expressing a single syn-tasiRNA (syn-tasiR-TSWV) against TSWV from different precursor positions. (**A**) Diagram of the syn-tasiRNA constructs. Positions including syn-tasiR-TSWV are highlighted in red. Other details are as in Figure 1A. (**B**) Northern blot detection of syn-tasiR-TSWV and miR173 in RNA preparations from agroinfiltrated leaves at 2 days post-agroinfiltration. The graph at top shows the mean (*n* = 3) + standard deviation (SD) syn-tasiR-TSWV (light red) and miR173 (orange) relative accumulation (*35S:syn-tasiR-TSWV* + *35S:AtMIR173* = 1.0). Other details are as in Figure 1C and D. (**C**) Photos at 7 days post-inoculation (dpi) of agroinfiltrated *Nicotiana benthamiana* leaves further inoculated with TSWV. Local lesions typical of TSWV infection are visible in some cases. (**D**) Two-dimensional line graph showing, for each of the six-plant sets listed in the box, the percentage of symptomatic plants per day during 20 dpi. (**E**) Bar graph representing the mean (*n* = 6) + SD absorbance obtained in double antibody enzyme-linked immunosorbent assays on the indicated samples collected at 10 and 20 dpi, as an indirect estimate of TSWV accumulation.

The sequence corresponding to the most efficient anti-TSWV amiRNA identified in previous amiRNA screenings (31) was introduced independently as a single syn-tasiRNA (named syn-tasiR-TSWV) in positions 3’D2[+], 3’D3[+], 3’D4[+] and 3’D5[+] of *AtTAS1c* in *pMDC32B-AtTAS1c-D2-B/c*, to generate the *35S:syn-tasiR-TSWV-D2*, *35S:syn-tasiR-TSWV-D3*, *35S:syn-tasiR-TSWV-D4* and *35S:syn-tasiR-TSWV-D5* constructs, respectively (Figure 5A). The control construct *35S:syn-tasiR-GUS_Sly_-D2* expressing a control syn-tasiRNA [(syn-tasiR-GUS_Sly_, without predicted off-targets in *Solanum lycopersicum* (31)] from position 3’D2[+] was also generated.

To begin, the accumulation of syn-tasiR-TSWV expressed from the different constructs was analyzed. Each syn-tasiRNA construct was independently co-agroinfiltrated together with the *35S:MIR173* construct expressing miR173, required for *AtTAS1c*-based syn-tasiRNA biogenesis in non-Arabidopsis species (17). For comparative purposes, the *35S:GUS* construct was infiltrated alone or in combination with *35S:MIR173*. In all cases, constructs were agroinfiltrated in one leaf of three different *N. benthamiana* plants, and infiltrated leaves were collected 2 days post agroinfiltration (dpa). RNA-blot assays of RNA preparations from agroinfiltrated leaves revealed that syn-tasiR-TSWV accumulated at higher and lower levels when expressed at position 3’D2[+] and 3’D5[+], respectively, while intermediate levels were detected when expressed from positions 3’D3[+] and 3’D4[+] (Figure 5B). Importantly, co-expressed miR173 accumulated to similar levels in all cases thus suggesting that differences in syn-tasiR-TSWV levels were not due to variable miR173 accumulations (Figure 5B).

Next, to functionally analyze anti-TSWV syn-tasiRNA constructs, six *N. benthamiana* plants were first agroinfiltrated with each of the constructs, and 2 days later mechanically inoculated with a well-characterized TSWV infectious extract (31). As negative controls, a set of plants was agroinfiltrated solely with the *35S:GUS* construct, and one other set was agroinfiltrated with *35S:syn-tasiR-GUS_Sly_-D2* and subsequently inoculated with TSWV. Leaves co-agroinfiltrated with *35S:syn-tasiR-TSWV-D2* or *35S:syn-tasiR-TSWV-D3* showed no lesions when compared with leaves expressing the syn-tasiR-GUS_Sly_ control (Figure 5C). In contrast, leaves expressing syn-tasiR-TSWV from positions 3’D4[+] and 3’D5[+] showed few or similar number of necrotic lesions, respectively, compared to control leaves expressing syn-tasiR-GUS_Sly_ (Figure 5C). Interestingly, all the plants expressing *35S:syn-tasiR-TSWV-D2* were symptomless and did not accumulate TSWV during the whole length of the experiment (Supplementary Table S4). In contrast, at 10 dpi, all plants expressing *35S:syn-tasiR-TSWV-D5* already displayed symptoms, although with a small delay compared with *35S:syn-tasiR-GUS_Sly_-D2* control plants, and accumulated high levels of TSWV (Figure 5D and E; Supplementary Table S4). TSWV was also detected at 10 dpi in one and three asymptomatic plants expressing *35S:syn-tasiR-TSWV-D3* and *35S:syn-tasiR-TSWV-D4*, respectively (Figure 5D and E), which turned symptomatic 1–4 days later (Figure 5D). Taken together these results indicate that anti-TSWV resistance can be finely modulated with syn-tasiRNAs expressed from different positions of *AtTAS1c* precursors.

### Fine-tuning *FT* expression in Arabidopsis with syn-tasiRNAs that base-pair with target RNAs to different degrees

Next, we aimed to test if syn-tasiRNA efficacy could also be modulated through a different strategy. Based on previous *in vivo* analyses of the complementarity requirements for plant microRNA targeting (11), we wondered if syn-tasiRNA efficacy could be modulated by altering the degree of base-pairing between the syn-tasiRNA and the target RNA. In particular, we hypothesized that syn-tasiRNA efficacy might be progressively reduced while introducing an increasing number of mismatches at the syn-tasiRNA 3′ end.

**Figure 6.**
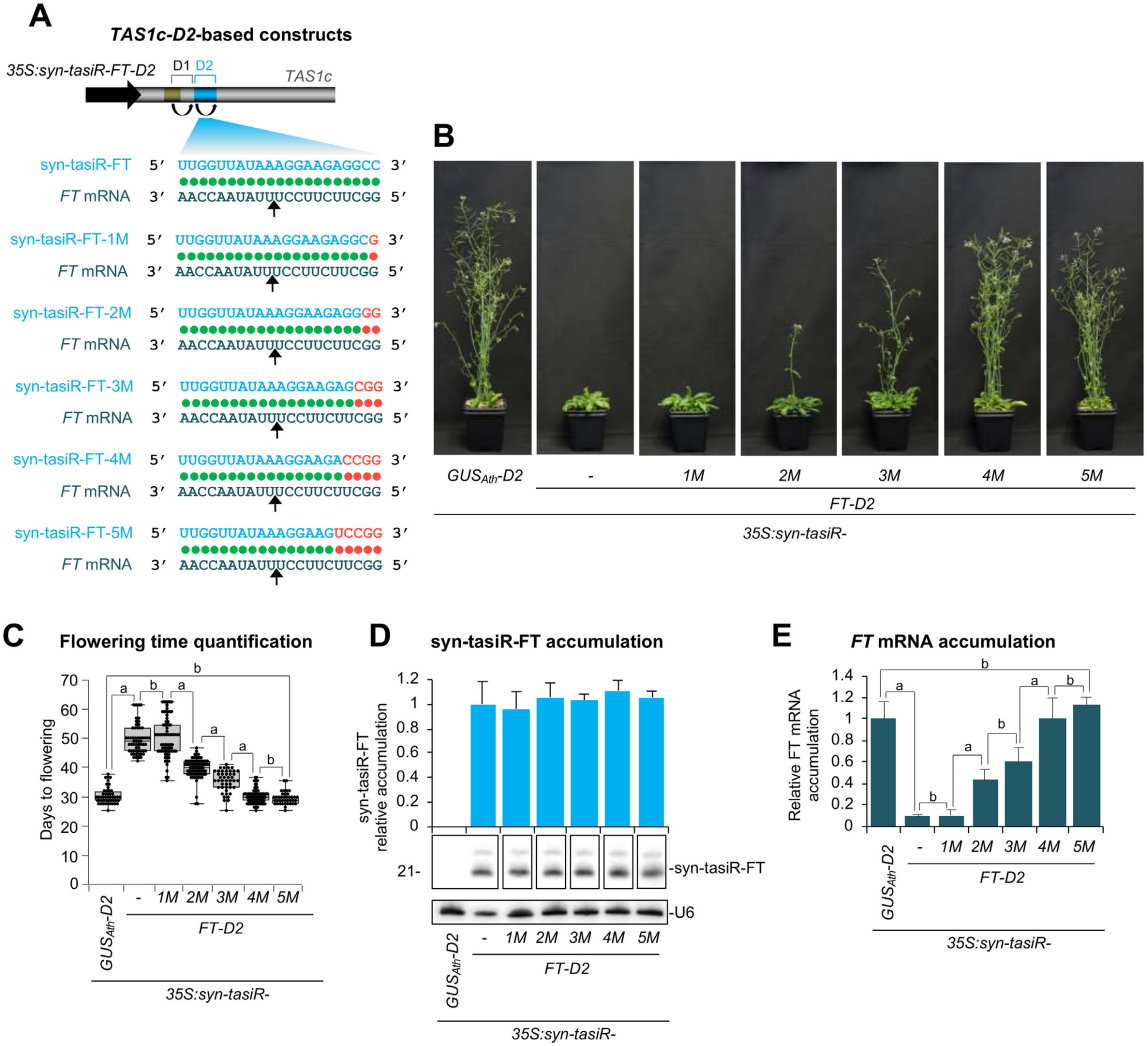
Functional analysis of *AtTAS1c-*based syn-tasiRNA constructs expressing syn-tasiRNAs with different degree of base-pairing with *FT* target mRNAs. (**A**) Diagram of the syn-tasiRNA constructs. Base-pairing and mismatches between each syn-tasiRNA and target RNA nucleotides are shown with green and red circles, respectively. Mutated nucleotides are shown in red. Other details are as in Figures 1A and 3A. (**B**) Representative images of Arabidopsis T1 transgenic plants expressing different syn-tasiRNA constructs. (**C**) Box plot representing the mean flowering time of Arabidopsis T1 transgenic plants expressing syn-tasiR-FT from different constructs. Other details are as in Figure 1C. (**D**) Northern blot detection of syn-tasiR-FT variants in Arabidopsis plants. The graph at top shows mean (*n* = 3) relative syn-tasiR-FT (light blue) levels + standard deviation (*35S:syn-tasiR-FT-D2* = 1.0). Other details are as in Figure 1C and D. (**E**) Accumulation of *FT* mRNA in Arabidopsis plants. Other details are as in Figures 1C, 3A and E.

This hypothesis was first tested using the Arabidopsis/*FT* system described above. Several syn-tasiRNA constructs were generated to express syn-tasiR-FT variants syn-tasiR-FT-1M (*35S:syn-tasiR-FT-D2–1M*), syn-tasiR-FT-2M (*35S:syn-tasiR-FT-D2–2M*), syn-tasiR-FT-3M (*35S:syn-tasiR-FT-D2–3M*), syn-tasiR-FT-4M (*35S:syn-tasiR-FT-D2–4M*) or syn-tasiR-FT-5M (*35S:syn-tasiR-FT-D2–5M*) with mutated 3’ends to induce 1, 2, 3, 4 or 5 consecutive mismatches in syn-tasiRNA/*FT* mRNA interactions (Figure 6A). In all cases syn-tasiR-FT variants were designed to not have significant predicted off-targets and were introduced at the 3’D2[+] position (Figure 6A). All these constructs were introduced into Arabidopsis Col-0, as well as the reference *35S:syn-tasiR-FT-D2* and control *35S:syn-tasiR-GUS_Ath_-D2* constructs. Analyses were done as described above in T1 lines.

All *35S:syn-tasiR-FT-D2* and *35S:syn-tasiR-FT-D2–1M* transformants flowered later than the average flowering time of the *35S:syn-tasiR-GUS_Ath_-D2* control lines, while only 97, 89, 59 and 8% of *35S:syn-tasiR-FT-D2–2M*, *35S:syn-tasiR-FT-D2–3M*, *35S:syn-tasiR-FT-D2–4M* and *35S:syn-tasiR-FT-D2–5M* transformants, respectively, did (Supplementary Table S5). Plants expressing the syn-tasiR-FT or syn-tasiR-FT-1M had the largest delay in flowering (∼17 days of delay) compared to controls (Figure 6B and C). In contrast, plants expressing syn-tasiR-FT-2M and syn-tasiR-FT-3M had a more modest delay in flowering, and plants expressing syn-tasiR-FT-4M or syn-tasiR-FT-5M had an average flowering time similar to that of *35S:syn-tasiR-GUS_Ath_-D2* control plants (Figure 6B and C). Importantly, RNA-blot assays revealed that syn-tasiRNA accumulation was similar in all lines (Figure 6D), thus suggesting that phenotypic differences were not due to differential syn-tasiRNA accumulation among lines. At last, target analysis showed that *FT* mRNA accumulation was drastically reduced in lines expressing syn-tasiR-FT or syn-tasiR-FT-1M, while lines expressing syn-tasiR-FT-2M and syn-tasiR-FT-3M accumulated intermediate levels of *FT* mRNA (Figure 6E). Levels of *FT* mRNA were similar in lines expressing syn-tasiR-FT-4M and syn-tasiR-FT-5M and controls (Figure 6E). These results suggest that silencing of endogenous genes can be finely modulated with syn-tasiRNAs by adjusting the degree of base-pairing between the 3′ end of the syn-tasiRNA and its target RNA. Importantly, the silencing effects were also maintained in Arabidopsis T2 progeny (Supplementary Figure S4).

### Modulation of antiviral resistance in *Nicotiana benthamiana* with syn-tasiRNAs that base-pair with target RNAs to different degrees

**Figure 7.**
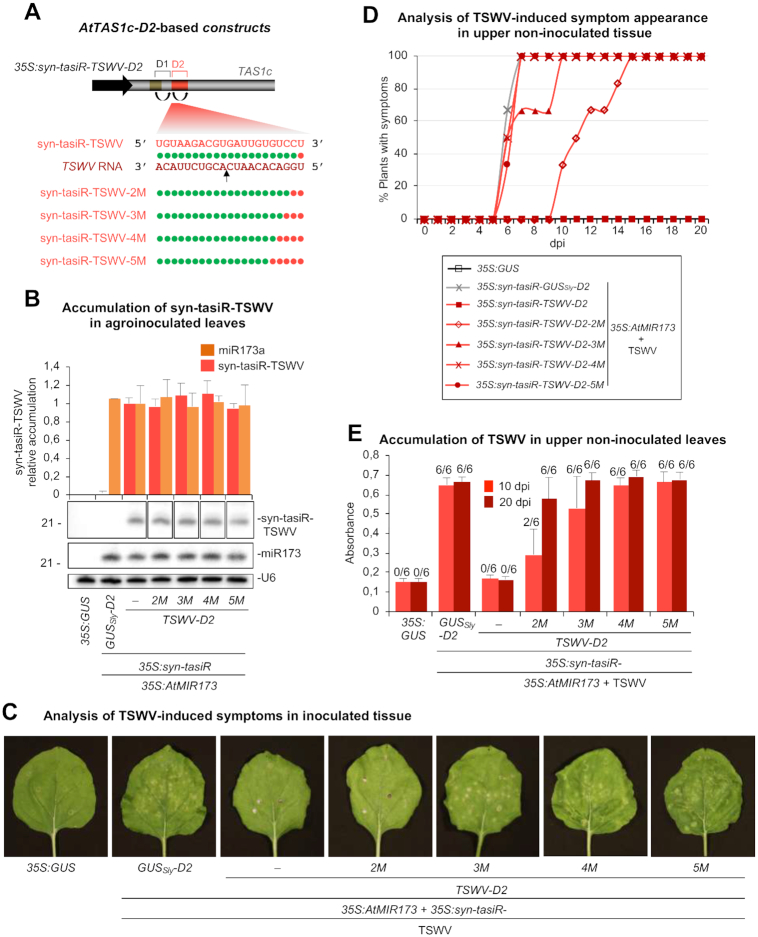
Functional analysis of *AtTAS1c-*based syn-tasiRNA constructs expressing syn-tasiRNAs with different degree of base-pairing with *TSWV* target RNAs. (**A**) Diagram of the syn-tasiRNA constructs. Other details are as in Figures 1A, 5A and 6A. (**B**) Northern blot detection of miR173 and syn-tasiR-TSWV variants in RNA preparations from agroinfiltrated leaves at 2 days post-agroinfiltration. The graph at top shows the mean (*n* = 3) + standard deviation (SD) syn-tasiR-TSWV (light red) and miR173 (orange) relative accumulation (*35S:syn-tasiR-TSWV* + *35S:AtMIR173* = 1.0). Other details are as in Figure 1C and D. (**C**) Photos at 7 days post-inoculation (dpi) of agroinfiltrated *Nicotiana benthamiana* leaves further inoculated with TSWV. Other details are as in Figure 5C. (**D**) Two-dimensional line graph showing, for each of the six-plant sets listed in the box, the percentage of symptomatic plants per day during 20 dpi. (**E**) Bar graph representing the mean (*n* = 6) + SD absorbance obtained in double antibody enzyme-linked immunosorbent assays on the indicated samples collected at 10 and 20 dpi, as an indirect estimate of TSWV accumulation.

Next, we speculated that silencing of exogenous transcripts could also be modulated by modifying the degree of base-pairing between syn-tasiRNAs and target RNAs. To test this, we used the *N. benthamiana*/TSWV system described above. Several syn-tasiRNA constructs were generated to express syn-tasiRNA variants syn-tasiR-TSWV-2M (*35S:syn-tasiR-TSWV-2M*), syn-tasiR-TSWV-3M (*35S:syn-tasiR-TSWV-3M*), syn-tasiR-TSWV-4M (*35S:syn-tasiR-TSWV-4M*) and syn-tasiR-TSWV-5M (*35S:syn-tasiR-TSWV-5M*) with mutated 3′ ends to produce 2, 3, 4 or 5 consecutive mismatches in syn-tasiRNA/TSWV interactions (Figure 7A and Supplementary Figure S5). In all cases, syn-tasiRNA variants were designed without significant predicted off targets in *N. benthamiana* and were also introduced at the 3’D2[+] position (Figure 7A). The experimental setup and analyses were the same as described above.

All syn-tasiRNA variants accumulated to similar levels in agroinfiltrated leaves, as observed also for co-expressed miR173 (Figure 7B). Plants expressing syn-tasiR-TSWV neither showed symptoms nor accumulated TSWV at any time (Figure 7C–E and Supplementary Table S6), as reported before (Figure 5). In contrast, all plants expressing syn-tasiR-TSWV-3M, syn-tasiR-TSWV-4M or syn-tasiR-TSWV-5M showed typical TSWV-induced local lesions, upper symptoms and high TSWV levels as early as 10 dpi (Figure 7C-D-E). At this specific time-point, 33% of the plants expressing syn-tasiR-TSWV-2M displayed symptoms, although delayed compared to controls, and accumulated TSWV, while all six plants were infected at 20 dpi (Figure 7D and E; Supplementary Table S6). All in all, our results suggest that resistance to TSWV can also be finely modulated with syn-tasiRNAs having distinct base-pairing properties.

### Generation and functional analysis of a universal syn-tasiRNA vector for fine-tuning gene silencing in plants

**Figure 8.**
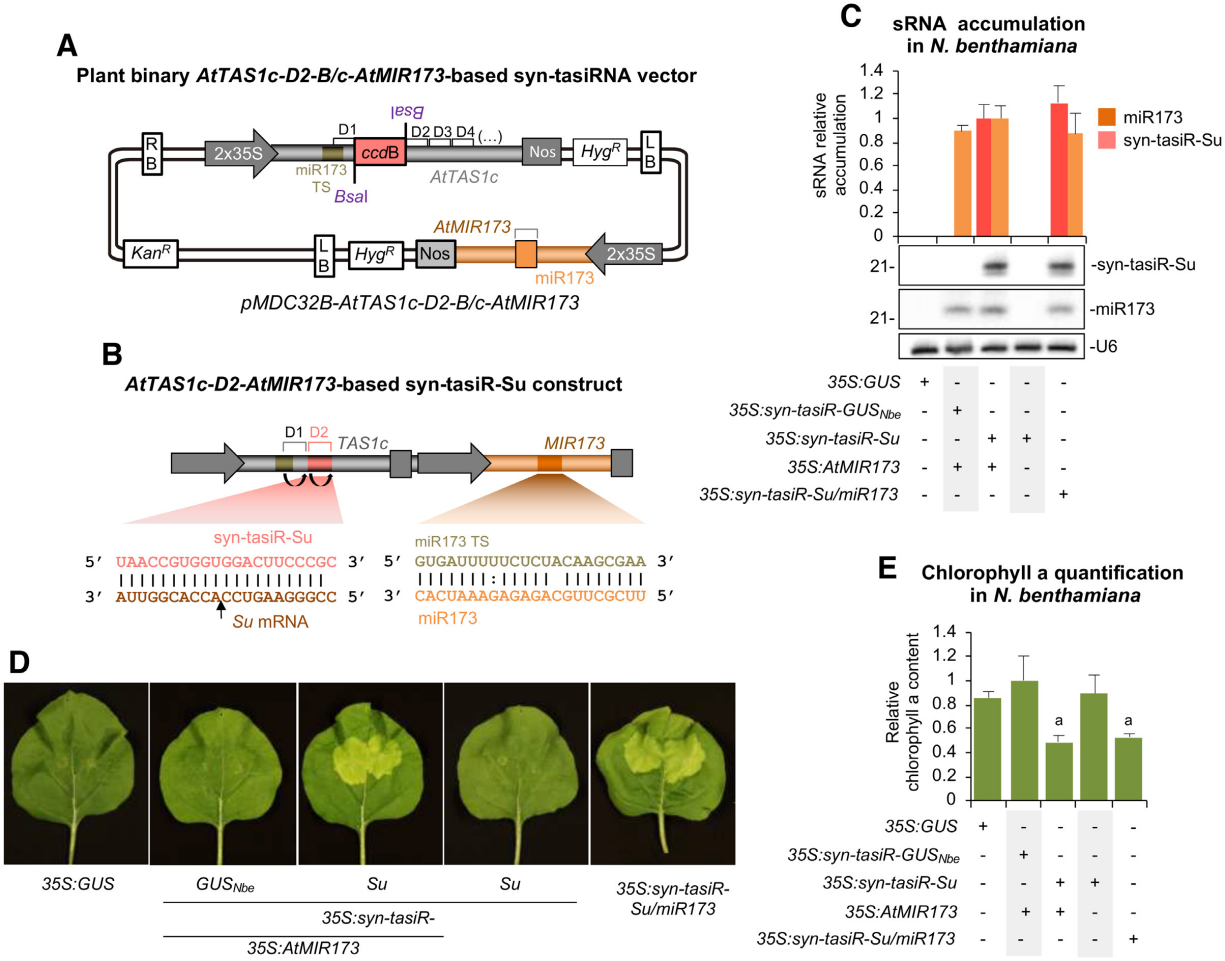
Functional analysis of *pMDC32B-AtTAS1c-D2-B/c-AtMIR173* in *Nicotiana benthamiana*. (**A**) Diagram of the vector. *AtMIR173* and miR173 sequences are shown in light and dark brown, respectively. Other details are as in Figure 2A. (**B**) Organization of the *35S:syn-tasiR-Su/miR173* construct expressing a syn-tasiRNA against *N. benthamiana SULPHUR*. Syn-tasiR-Su sequence introduced at the 3’D2[+] position in *AtTAS1c* is shown in light pink. Other details are as in Figures 1A and 8A. (**C**) Northern blot detection of syn-tasiR-Sy and miR173 in RNA preparations from agroinfiltrated leaves at 2 days post-agroinfiltration. The graph at top shows the mean (*n* = 3) + standard deviation syn-tasiR-Su (light pink) and miR173 (orange) relative accumulation (*35S:syn-tasiR-Su* + *35S:AtMIR173* = 1.0). Other details are as in Figure 1C and D. (**D**) Photos at 6 days post-agroinfiltration of leaves agroinfiltrated with different constructs. (**E**) Bar graph showing the relative content of chlorophyll a in patches agroinfiltrated with different constructs (*35S:syn-tasiR-Su* + *35S:AtMIR173* = 1.0). Bars with the letter ‘a’ are significantly different from that of sample *35S:syn-tasiR-GUS_Nb_* + *35S:AtMIR173* (*P* < 0.01 in pairwise Student's *t*-test comparisons).

As noted above, production of syn-tasiRNAs from *pMDC32B-AtTAS1c-D2-B/c*-derived constructs requires co-expression of miR173 in non-Arabidopsis species. Thus, to broaden the use of *AtTAS1c-D2*-based syn-tasiRNAs in any plant species, a DNA cassette including the Arabidopsis *AtMIR173* sequence flanked by regulatory sequences was introduced into *pMDC32B-AtTAS1c-D2-B/c*, downstream the syn-tasiRNA cassette, to generate the *pMDC32B-AtTAS1c-D2-B/c-AtMIR173* vector (Figure 8A). Importantly, the two BsaI sites present in *AtMIR173* were mutated with 2 nt substitutions aimed to preserve the authentic foldback structure of *AtMIR173* necessary for accurate DCL1-mediated processing (Supplementary Figure S6). Thus, the *pMDC32B-AtTAS1c-D2-B/c-AtMIR173* vector was engineered for the direct cloning of syn-tasiRNA(s) using BsaI (Supplementary Figure S1 and Protocol S1), while assuring the *in vivo* synthesis of syn-tasiRNAs due to the co-expression of the miR173 trigger.

The functionality of *pMDC32B-AtTAS1c-D2-B/c-AtMIR173* was assessed in a transient assay in *N. benthamiana*. A 21-nt guide sequence was designed to target *N. benthamiana SULPHUR* gene, and introduced into *pMDC32B-AtTAS1c-D2-B/c-AtMIR173* to generate the *35S:syn-tasiR-Su/miR173* construct (Figure 8B). For comparative purposes, the same 21-nt sequence was also introduced into *pMDC32B-AtTAS1c-D2-B/c* to produce the *35S:syn-tasiR-Su* construct. As a control, the 3*5S:syn-tasiR-GUS_Nbe_-D2* construct aimed to express a control syn-tasiRNA (syn-tasiR-GUS_Nbe_, without predicted off-targets in *N. benthamiana*) from position 3’D2[+] was also generated. As expected, leaves agroinfiltrated with *35S:syn-tasiR-Su/miR173* accumulated both syn-tasiR-Su and miR173, as revealed by RNA-blot assays from RNA preparations obtained 2 dpa (Figure 8C). Indeed, both types of sRNAs also accumulated to similar levels in leaves co-expressing *35S:syn-tasiR-Su* and *35S:AtMIR173* (Figure 8C), but were absent in control leaves agroinfiltrated with *35S:GUS*. Importantly, syn-tasiR-Su accumulation was dependent on miR173 expression, as samples expressing *35S:syn-tasiR-Su* in the absence of miR173 did not accumulate syn-tasiR-Su (Figure 8C). At 6 dpa, agroinfiltrated patches expressing syn-tasiR-Su from *35S:syn-tasiR-Su/miR173* or *35S:syn-tasiR-Su + 35S:AtMIR173* had a visually obvious bleached phenotype characteristic of *SULPHUR* knock-down (41), while patches expressing *35S:GUS* or *35S:syn-tasiR-GUS_Nbe_* + *35S:AtMIR173* did not (Figure 8D). The pigment analysis of agroinfiltrated patches confirmed a similar decrease in chorophyll content in bleached patches expressing *35S:syn-tasiR-Su/miR173* or *35S:syn-tasiR-Su + 35S:AtMIR173* compared to patches expressing control constructs (Figure 8E). All together, these results show that *pMDC32B-AtTAS1c-D2-B/c-AtMIR173*-based syn-tasiRNA constructs are functional in a plant species unrelated to Arabidopsis.

## DISCUSSION

In this work we describe two independent strategies based on art-sRNAs for fine-tuning gene expression in plants. In particular, we report that the degree of induced silencing can be finely modulated by (i) expressing the art-sRNA from different positions in *AtTAS1c* precursors, and (ii) altering the degree of base-pairing between the art-sRNA and its target RNA. The two strategies were used to modulate the accumulation of endogenous and exogenous targets in Arabidopsis and *N. benthamiana*.

### The DCL4-processing position from which *AtTAS1c*-based syn-tasiRNAs are expressed is a major determinant of syn-tasiRNA efficacy

Our results describing the distinct degrees of silencing of endogenous *FT* and *CH42* in Arabidopsis, and of exogenous TSWV in *N. benthamiana*, with syn-tasiRNAs expressed from different *AtTAS1c* positions indicate that syn-tasiRNA efficacy can be finely controlled. It is not surprising that such positional effect was not reported before, as no systematic study of the accumulation of the same single syn-tasiRNA from different precursor positions was ever done. The majority of previous studies reported the simultaneous co-expression of two–five different syn-tasiRNAs from diverse *AtTAS* precursors. For instance, multiple syn-tasiRNAs were expressed from *AtTAS1a* positions 3’D6[+] (42), from *AtTAS1c* positions 3’D3[+]/3’D4[+] (18, 24), 3’D3[+]-3’D6[+] (27,31) and 3’D3[D3]-3’D7[+] (26), and from *AtTAS3a* positions 5’D2[+]-5’D7[+] (25) and 5’D7[+]/5’D8[+] (18). Only in one study, a single syn-tasiRNA sequence against Arabidopsis *FAD2* was expressed, but as five consecutive copies introduced in *AtTAS1c* positions 3’D2[+]-3’D6[+] (20). Because northern blot analysis revealed a single band corresponding to the same syn-tasiRNA sequence, the accumulation of each syn-tasiRNA species expressed from a particular position could not be assessed. Interestingly, two studies have reported weak silencing by syn-tasiRNAs expressed from positions distal to the trigger miRNA target site, such as 3’D6[+] and 5’D7[+]/5’D8[+] in *AtTAS1a* and *AtTAS3a*, respectively (18,42). These results indicate that the low efficacies of syn-tasiRNAs expressed from distal positions are not exclusive to *AtTAS1c* precursors, and, perhaps, the position from which the syn-tasiRNA is released also determines syn-tasiRNA accumulation and efficacy in several other precursors. In any case, the reasons explaining the gradual decrease in syn-tasiRNA accumulation as syn-tasiRNA is released from more distal positions are unknown. What it is certain is that the release of the correct syn-tasiRNA sequence from a particular precursor position requires the correct processing by DCL4 at the previous positions. However, it is possible that inaccuracy of DCL4 processing compromises the release of distal syn-tasiRNAs of the correct sequence, or that DCL4 processivity decreases over time and, consequently, DCL4 is released form *AtTAS1c* precursors. If any or both of these possibilities occur, the amount of correct syn-tasiRNA sequences is expected to decrease as syn-tasiRNAs are expressed from positions more distal to the trigger miRNA target site. This may limit the total number of effective syn-tasiRNAs that can be produced from a single *AtTAS1c* precursor. Moreover, if DCL4 levels are limiting, the overexpression of DCL4 together with the syn-tasiRNA precursor might enhance overall syn-tasiRNA production and thus increase the number of effective syn-tasiRNAs produced from the same precursor.

At last, one important finding was that *AtTAS1c-*based syn-tasiRNAs accumulated to the highest levels and were most effective when expressed from position 3’D2[+]. This suggests that the resistance against TSWV and PSTVd previously obtained with *AtTAS1c*-based syn-tasiRNA cassettes introduced in 3’D3[+]-3’D6[+]/3’D7[+] (26,27) may be improved if expressing the same syn-tasiRNA cassettes from 3’D2[+]. Also, it is very likely that the weak silencing reported for *AtTAS1a* and *AtTAS3a*-based syn-tasiRNAs expressed from positions 3’D6[+] and 5’D7[+]/5’D8[+], respectively (18,42) is a consequence of expressing the syn-tasiRNAs from distal positions.

### Syn-tasiRNA efficacy depends on the degree of base-pairing between the syn-tasiRNA and its target RNA

Previous studies on the complementarity requirements for productive sRNA–target RNA interactions in plants examined miRNA function *in vivo* through the analysis of mRNA accumulation only (12,43–44), and more recently of both mRNA and protein accumulation (11). These studies reported that target site sensitivity to mismatches is higher at the miRNA 5′ end, with mismatches at the miRNA 3’end being much less deleterious (11–12,44–45). More generally, these studies revealed that plant target sites have a range of intrinsic efficacies determined by the degree of base-pairing between miRNAs and target RNAs, in order to fine-tune miRNA activity. For example, differential efficacies of miR172 target sites due to variations in target site complementarities explain the variation of spike density among different barley cultivars (46).

We sought to exploit this knowledge to design art-sRNAs with distinct efficacies. We found that the presence of one mismatch at the syn-tasiRNA 3′end was tolerated without affecting silencing, while 2–3 and 4–5 consecutive mismatches at this same end significantly diminished or abolished, respectively, syn-tasiRNA activity (Figure 6). Interestingly, a systematic study on miR160 target site sequence complementarity requirements reported that miR160 targeting was unaffected, diminished or completely abolished when 1–3, 4–5 and >6 mismatches, respectively, were present at the 5′ end of the target site (11). Therefore, the optimal efficacies observed here for syn-tasiRNAs producing 0–1 mismatches when base-pairing with target RNAs (syn-tasiR-FT and syn-tasiR-FT-1M, respectively) is not surprising. Indeed, it has been proposed that mismatches at the 5′ end of target sites may allow faster RISC dissociation rates compared to perfectly paired miRNA/target RNA complexes, thus explaining the occurrence of such type of target sites in plants and humans (11,47). In contrast, the different effects of 2–5 consecutive mismatches at the 5’ end of target sites between the work by Liu *et al.* (11) and here may just be explained by the distinct experimental systems used.

### A modular system for fine-tuning gene expression in plants

**Figure 9.**
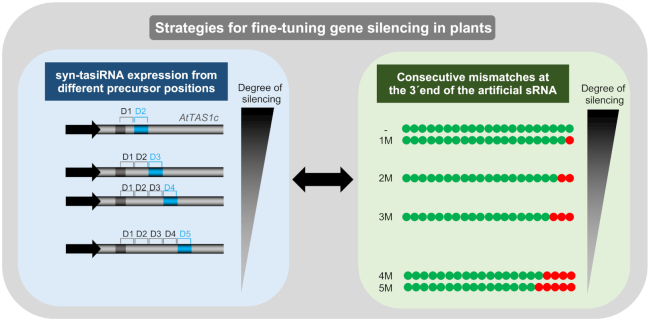
Modular system based on artificial sRNAs for fine-tuning gene silencing in plants. The double arrow indicates that both strategies are complementary. Other details are as in Figure 6A.

The two strategies presented here might be complementary and used in combination to further extend the range of induced silencing. Each strategy can be seen as an independent module of a unique system for fine-tuning plant gene expression through art-sRNAs (Figure 9). This ‘modular’ system can be used in a one-module mode, as reported here when analyzing the effect on silencing of syn-tasiRNA precursor position while maintaining the degree of base-pairing between syn-tasiRNA and target RNA (3′D2[+]-3’D5[+]/0–1 mismatches, (Figures 3–5) or vice versa (3’D2[+]/0–5 mismatches, Figures 6 and 7). Interestingly, our system might also function in a two-module mode to allow for a plethora of silencing levels based on the multiple possible combinations between the four *AtTAS1c* positions 3′D2[+]-3’D5[+] and the three art-sRNA/target RNA base-pairing configurations (0/1–3 mismatches) known to have an effect on silencing efficacy. Interestingly, while the strategy based on the precursor position is exclusive of syn-tasiRNAs, the possibility of adjusting the degree of base-pairing between the art-sRNA and target RNAs can certainly be applied also to amiRNAs. In any case, optimal silencing levels might be obtained by expressing a syn-tasiRNA against a target site with 0–1 mismatches at the 3’end of the syn-tasiRNA and released from *AtTAS1c* position 3’D2[+]. The generation of a new set of ‘B/c’ vectors for introducing syn-tasiRNAs downstream position 3’D1[+] in *AtTAS1c* should facilitate the generation of syn-tasiRNA constructs for both high silencing or fine-tune control of gene expression.

### Control of RNAi efficacy in plants

Control of RNAi efficacy in plants has mainly focused in obtaining the highest silencing levels through the overexpression of RNAi transgenes with strong promoters, or by controlling the place and moment of transgene expression through tissue-specific or inducible promoters, respectively (48,49). However, intrinsic properties related to the plant transformation process such as transgene insertion site and copy number may lead to lines with different levels of transgene expression (50). Alternatively, a number of RNAi phenotypes could be obtained by targeting different regions of the same target RNA (30). However, in all these cases variations on the degree of RNAi are circumstantial and uncontrolled.

The possibility of programming art-sRNAs to induce a particular degree of silencing is particularly interesting in at least two scenarios: (i) in functional analyses of vital genes requiring suboptimal silencing levels to prevent lethality, and (ii) in gene function or crop improvement studies aiming to obtain a wide spectrum of RNAi phenotypes by generating an allelic series of individuals with different degrees of silencing. The ability to specifically downregulate target gene expression as opposed to completely block it makes art-sRNAs a unique tool for fine-tuning gene expression in plants. Still, a better knowledge of the molecular mechanisms dictating art-sRNA biogenesis, mode of action and targeting efficacy is required to further refine these strategies for more efficient fine-tuning of plant gene expression. Last but not least, the optimization of methods for producing and delivering art-sRNA precursors topically, in a GMO-free manner, is also necessary for applying these RNA-based technologies to crops in line with international laws.

## Supplementary Material

gkaa343_Supplemental_File

## Notes

Present address: Maria Spada, Department of Agriculture, Food and Environment, University of Pisa, Italy.
