# Supplementary material for: Fine-tune control of targeted RNAi efficacy by plant artificial small RNAs
Source: Nucleic Acids Res. 2020 May 12;48(11):6234–50. doi: 10.1093/nar/gkaa343 (PMC7293048; doi:10.1093/nar/gkaa343)
Supplement: gkaa343_Supplemental_File [file gkaa343_supplemental_file.pdf]

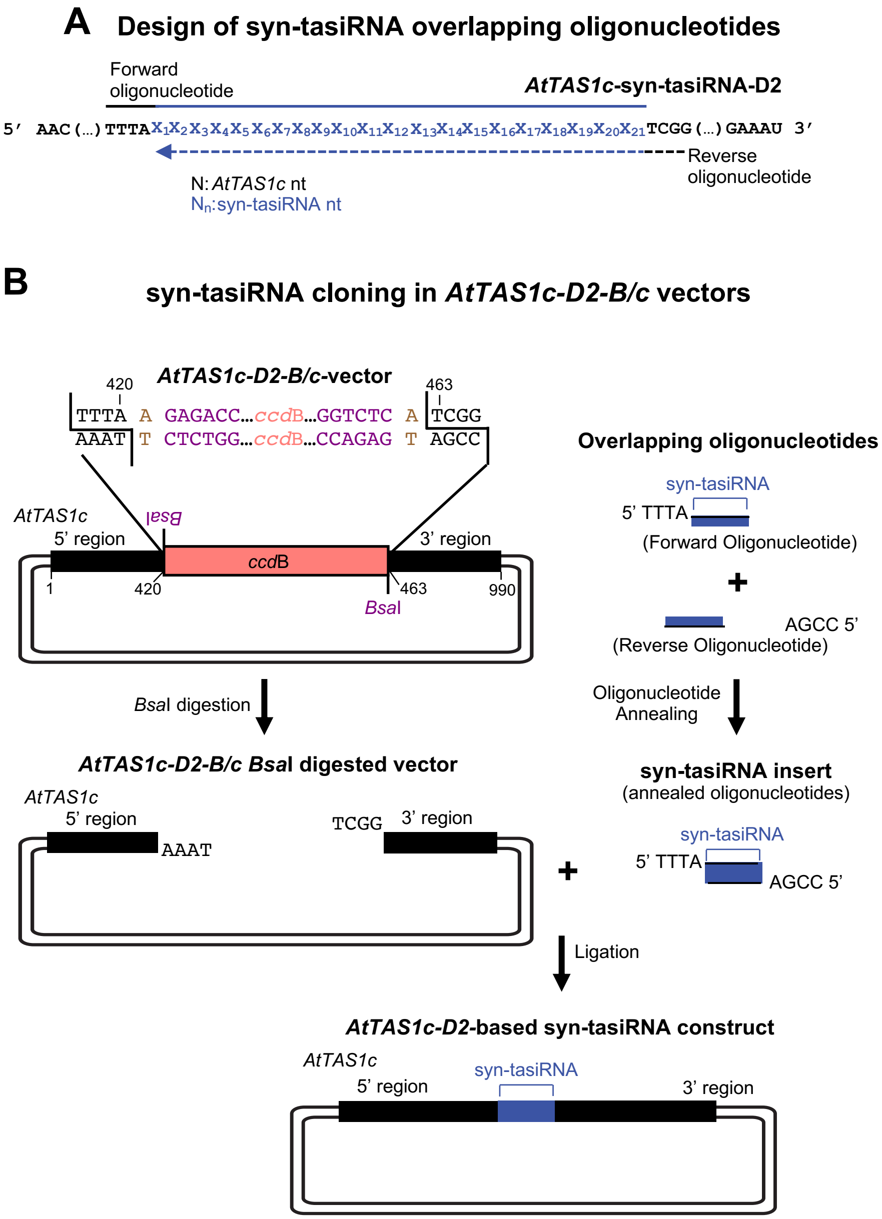

Supplementary Figure S1. Direct syn-tasiRNA cloning in *AtTAS1c-D2-B/c*-based vectors including a modified version of *AtTAS1c* with a *ccd*B cassette flanked by two *Bsa*I sites (*Bsa*I/*ccd*B or "B/c" vectors).
(A) Design of two overlapping oligonucleotides for syn-tasiRNA cloning. Sequence covered by the forward and reverse oligonucleotides are represented with continuous or dotted lines, respectively.
(B) Diagram of the steps for syn-tasiRNA cloning in *AtTAS1c-D2-B/c* vectors. The syn-tasiRNA insert obtained after annealing the two overlapping oligonucleotides has 5' TTTA and 5'-CCGA overhangs and is directly inserted into the *Bsa*I-linearized *AtTAS1c-D2-B/c* vector. Nucleotides of the *Bsa*I sites and arbitrary nucleotides used as spacers between the *Bsa*I recognition site and the *AtTAS1c* sequence are in purple and light brown, respectively. Other details are as in Panel A.

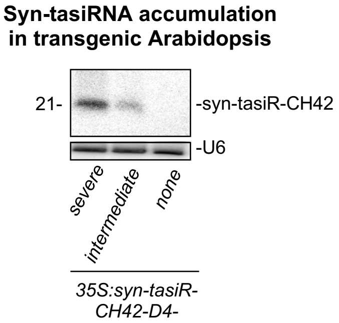

Supplementary Figure S2. Northern blot detection of syn-tasiR-CH42 in Arabidopsis T1 *35S:syn-tasiR-CH42-D4* transgenic lines. Each sample corresponds to a pool of at least 3 independent lines showing either severe, intermediate or no phenotype. The U6 RNA blot is shown as a loading control.

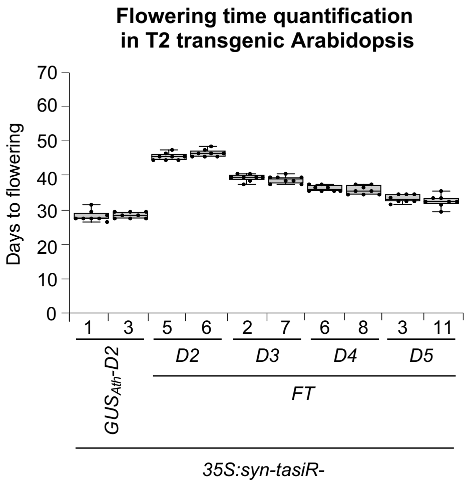

Supplementary Figure S3. Jitter box plot representing the mean flowering time (days to flowering) of Arabidopsis T2 transgenic lines expressing syn-tasiRNA constructs. Selected T2 lines originated from representative T1 lines with a flowering time similar to the mean of the corresponding group.

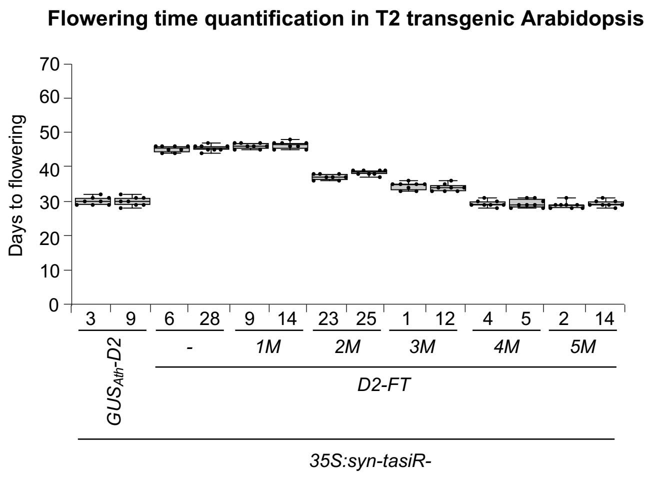

Supplementary Figure S4. Jitter box plot representing the mean flowering time (days to flowering) of Arabidopsis T2 transgenic lines expressing syn-tasiRNA constructs. Selected T2 lines originated from representative T1 lines with a flowering time similar to the mean of the corresponding group.

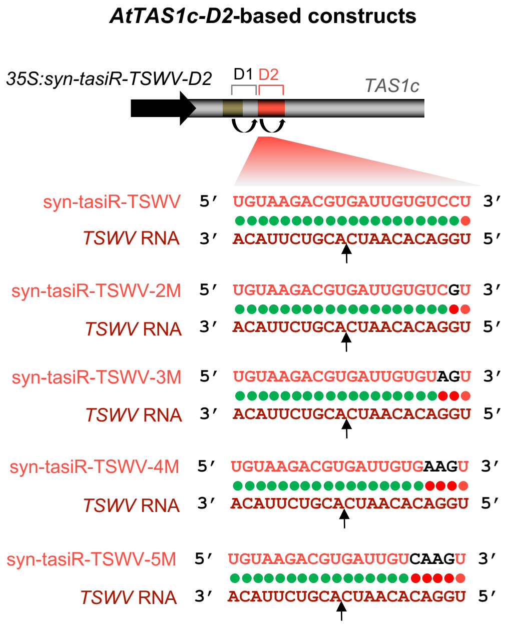

Supplementary Figure S5. Diagram of the syn-tasiRNA constructs. Base-pairing and mismatches between syn-tasiRNA and target RNA nucleotides are shown with green and red circles, respectively. Mutated nucleotides are shown in black. Other details are as in Figures 1A and 3A.

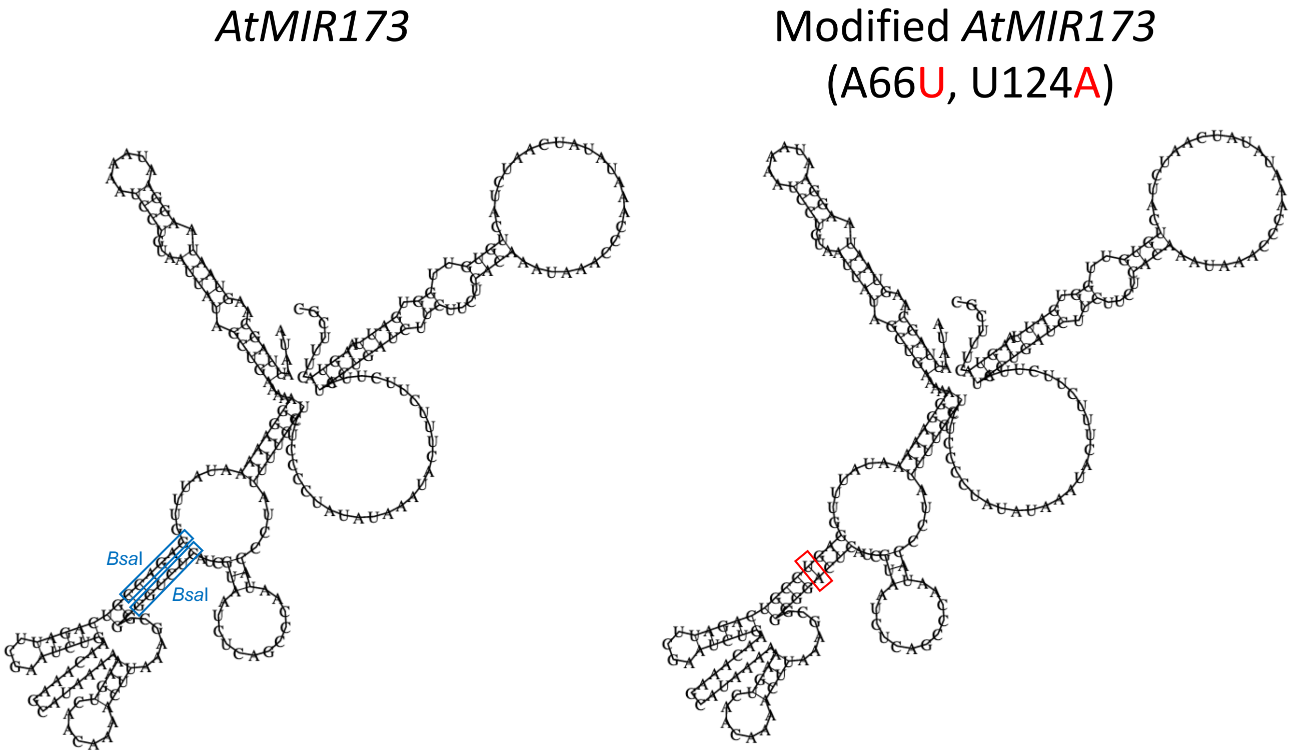

Supplementary Figure S6. M-fold predicted secondary structures of wild-type and modified *AtMIR173* precursors. Functional and disrupted *Bsa*I sites are highlighted in blue and red, respectively.

Alberto Carbonell Lab (https://www.AlbertoCarbonellLab.com)
Instituto de Biología Molecular y Celular de Plantas
Valencia (Spain)
Updated 09/12/2019

Supplementary Protocol S1

Protocol to design and clone syn-tasiRNAs downstream the 3'D1[+] position in *AtTAS1c-D2-Bsa*I/*ccd*B-based ('B/c') vectors containing *AtTAS1c* precursor.

1. Selection of the syn-tasiRNA sequence(s)

Use the Syn-tasiRNA Designer app from the P-SAMS webtool at http://p-sams.carringtonlab.org/syntasi/designer.

2. Design of syn-tasiRNA oligonucleotides

Use the Syn-tasiRNA Designer app from the P-SAMS webtool at http://p-sams.carringtonlab.org/syntasi/designer.

2.2.1 Sequence of the *AtTAS1c* cassette containing the syntasiRNA(s)

The following FASTA sequence includes two syn-tasiRNA sequences inserted in the *AtTAS1c* precursor sequence downstream position 3'D1[+]:

>syn-tasiRNA-1 and syn-tasiRNA-2 in *AtTAS1c*

AAACCTAAACCTAAACGGCTAAGCCCGACGTCAAATACCAAAAAGAGAAAAACAAGAGCGCCGT

CAAGCTCTGCAAATACGATCTGTAAGTCCATCTTAACACAAAAGTGAGATGGGTTCTTAGATCA

TGTTCCGCCGTTAGATCGAGTCATGGTCTTGTCTCATAGAAAGGTACTTTCGTTTACTTCTTTT

GAGTATCGAGTAGAGCGTCGTCTATAGTTAGTTTGAGATTGCGTTTGTCAGAAGTTAGGTTCAA

TGTCCCGGTCCAATTTTCACCAGCCATGTGTCAGTTTCGTTCCTTCCCGTCCTCTTCTTTGATT

TCGTTGGGTTACGGATGTTTTCGAGATGAAACAGCATTGTTTTGTTGTGATTTTTCTCTACAAG

CGAATAGACCA**TTTA**$X_1X_2X_3X_4X_5X_6X_7X_8X_9X_{10}X_{11}X_{12}X_{13}X_{14}X_{15}X_{16}X_{17}X_{18}X_{19}X_{20}X_{21}X_1X_2X_3X_4$

$X_5X_6X_7X_8X_9X_{10}X_{11}X_{12}X_{13}X_{14}X_{15}X_{16}X_{17}X_{18}X_{19}X_{20}X_{21}$**TCGG**TGGATCTTAGAAAATTATTCTAAG

TCCAACATAGCGTATTCTAAGTTCAACATATCGACGAACTAGAAAAGACATTGGACATATTCCA

GGATATGCAAAAGAAAACAATGAATATTGTTTTGAATGTGTTCAAGTAAATGAGATTTTCAAGT

CGTCTAAAGAACAGTTGCTAATACAGTTACTTATTTCAATAAATAATTGGTTCTAATAATACAA

AACATATTCGAGGATATGCAGAAAAAAAGATGTTTGTTATTTTGAAAAGCTTGAGTAGTTTCTC

TCCGAGGTGTAGCGAAGAAGCATCATCTACTTTGTAATGTAATTTTCTTTATGTTTTCACTTTG

TAATTTTATTTGTGTTAATGTACCATGGCCGATATCGGTTTTATTGAAAGAAAATTTATGTTAC

TTCTGTTTTGGCTTTGCAATCAGTTATGCTAGTTTTCTTATACCCTTTCGTAAGCTTCCTAAGG
AATCGTTCATTGATTTCCACTGCTTCATTGTATATTAAAACTTTACAACTGTATCGACCATCAT
ATAATTCTGGGTCAAGAGATGAAAATAGAACACCACATCGTAAAGTGAAAT

Where:

-**X** is a DNA base of the syn-tasiRNA-1 sequence, and the subscript number is the base position in the syn-tasiRNA-1 21-mer

-**X** is a DNA base of the syn-tasiRNA-2 sequence, and the subscript number is the base position in the syn-tasiRNA-2 21-mer

-**X** is a DNA base of the *AtTAS1c* precursor included in the oligonucleotides required to clone the syn-tasiRNA insert in B/c vectors

-X is a DNA base of the *AtTAS1c* precursor

Note that in general, X_1=T and X_1=T for syn-tasiRNA association with AGO1.

In the sequence above, replace the sequences $X_1X_2X_3X_4X_5X_6X_7X_8X_9X_{10}X_{11}X_{12}X_{13}X_{14}X_{15}X_{16}X_{17}X_{18}X_{19}X_{20}X_{21}$ and $X_1X_2X_3X_4X_5X_6X_7X_8X_9X_{10}X_{11}X_{12}X_{13}X_{14}X_{15}X_{16}X_{17}X_{18}X_{19}X_{20}X_{21}$ by the sequences of syn-tasiRNA_1 and syn-tasiRNA_2, respectively.

2.2.2. Sequence of the syn-tasiRNA oligonucleotides

The sequences of the two syn-tasiRNA oligonucleotides are:

-Sense oligonucleotide (46 b):

TTTA$X_1X_2X_3X_4X_5X_6X_7X_8X_9X_{10}X_{11}X_{12}X_{13}X_{14}X_{15}X_{16}X_{17}X_{18}X_{19}X_{20}X_{21}X_1X_2X_3X_4X_5X_6X_7X_8X_9X_{10}X_{11}X_{12}X_{13}X_{14}X_{15}X_{16}X_{17}X_{18}X_{19}X_{20}X_{21}$

-Antisense oligonucleotide (46 b):

CCGA$Y_{21}Y_{20}Y_{19}Y_{18}Y_{17}Y_{16}Y_{15}Y_{14}Y_{13}Y_{12}Y_{11}Y_{10}Y_9Y_8Y_7Y_6Y_5Y_4Y_3Y_2Y_1Y_{21}Y_{20}Y_{19}Y_{18}Y_{17}Y_{16}Y_{15}Y_{14}Y_{13}Y_{12}Y_{11}Y_{10}Y_9Y_8Y_7Y_6Y_5Y_4Y_3Y_2Y_1$

Where:

-$X_1X_2X_3X_4X_5X_6X_7X_8X_9X_{10}X_{11}X_{12}X_{13}X_{14}X_{15}X_{16}X_{17}X_{18}X_{19}X_{20}X_{21}$=syn-tasiRNA-1 sequence

-$X_1X_2X_3X_4X_5X_6X_7X_8X_9X_{10}X_{11}X_{12}X_{13}X_{14}X_{15}X_{16}X_{17}X_{18}X_{19}X_{20}X_{21}$=syn-tasiRNA-2 sequence

-$Y_{21}Y_{20}Y_{19}Y_{18}Y_{17}Y_{16}Y_{15}Y_{14}Y_{13}Y_{12}Y_{11}Y_{10}Y_{9}Y_{8}Y_{7}Y_{6}Y_{5}Y_{4}Y_{3}Y_{2}Y_{1}$=syn-tasiRNA-1 reverse-complement sequence

-$Y_{21}Y_{20}Y_{19}Y_{18}Y_{17}Y_{16}Y_{15}Y_{14}Y_{13}Y_{12}Y_{11}Y_{10}Y_{9}Y_{8}Y_{7}Y_{6}Y_{5}Y_{4}Y_{3}Y_{2}Y_{1}$=syn-tasiRNA-2 reverse-complement sequence

Example

The sequences of the two oligonucleotides to clone syn-tasiRNAs 'syn-tasiR-Trich' (**TCCCATTCGATACTGCTCGCC**) and 'syn-tasiR-Ft' (**TTGGTTATAAAGGAAGAGGCC**) in positions 3'D3[+] and 3'D4[+] of *AtTAS1c,* respectively, are:

-Sense oligonucleotide (46 b):

TTTATCCCATTCGATACTGCTCGCCTTGGTTATAAAGGAAGAGGCC

-Antisense oligonucleotide (46 b):

CCGAGGCCTCTTCCTTTATAACCAAGGCGAGCAGTATCGAATGGGA

3. Cloning of the syn-tasiRNA sequence(s) in AtTAS1c-D2-B/c-based vectors

Notes:

-Available AtTAS1c-D2-B/c vectors are listed in Table I at the end of the section.

-AtTAS1c-D2-B/c-*based vectors must be propagated in a* ccd*B resistant* E. coli *strain such as DB3.1.*

-Alternatively, Bsa*I digestion of the B/c vector and subsequent ligation of the amiRNA oligonucleotide insert can be done in separate reactions*

3.1. Oligonucleotide annealing

-Dilute sense oligonucleotide and antisense oligonucleotide in sterile H_2O to a final concentration of 100 µM.

-Prepare Oligo Annealing Buffer:

60 mM Tris-HCl (pH 7.5)

500 mM NaCl

60 mM $MgCl_2$

10 mM DTT

Note: *Prepare 1 ml aliquots of Oligo Annealing Buffer and store at -20*$_0$*C.*

-Assemble the annealing reaction in a PCR tube as described below:

Forward oligonucleotide (100 µM)	2 µL
Reverse oligonucleotide (100 µM)	2 µL
Oligo Annealing Buffer	46 µL
Total volume	50 µL

The final concentration of each oligonucleotide is 4 µM.

-Use a thermocycler to heat the annealing reaction 5 min at 94°C and then cool down (0.05ºC/sec) to 20°C.

-Dilute the annealed oligonucleotides just prior to assembling the digestion-ligation reaction as described below:

Annealed oligonucleotides	3 µL
dH_2O	37 µL
Total volume	40 µL

The final concentration of each oligonucleotide is 0.15 µM.

Note*: Do not store the diluted oligonucleotides.*

3.2. Digestion-ligation reaction

- Assemble the digestion-ligation reaction as described below:

B/c vector (x ug/uL)	Y µL (50 ng)
Diluted annealed oligonucleotides	1 µL
10x T4 DNA ligase buffer	1 µL
T4 DNA ligase (400 U/µL)	1 µL
*Bsa*I (10U/ µL, NEB)	1 µL
dH_2O	to 10 µL

Total volume 10 µL

Prepare a negative control reaction lacking *Bsa*I.

-Mix the reactions by pipetting. Incubate the reactions at room temperature for 5 minutes at 37°C.

3.3. *E.coli* transformation and analysis of transformants

-Transform 1-5 ul of the digestion-ligation reaction into an *E. coli* strain that doesn't have *ccd*B resistance (e.g. DH10B, TOP10, …) to do counter-selection.

-Pick two colonies/construct, grow LB-Kan (100 mg/ml) cultures and purify plasmids.

-Sequence with appropriate primers: M13-F (CCCAGTCACGACGTTGTAAAACGACGG) and M13-R (CAGAGCTGCCAGGAAACAGCTATGACC) for *pENTR*-based vectors; attB1 (ACAAGTTTGTACAAAAAAGCAGGCT) and attB2 (ACCACTTTGTACAAGAAAGCTGGGT) primers for *pMDC32B*-based vectors).

Table I: *Bsa*I/*ccd*B-based ('B/c') vectors for direct cloning of syn-tasiRNAs downstream position 3'D1[+] in *AtTAS1c* precursor.

Vector	Small RNA expressed	Bacterial antibiotic resistance	Plant antibiotic resistance	GATEWAY use	Backbone	Promoter of syn-tasiRNA cassette	Terminator of syn-tasiRNA cassette	Plant species tested
pENTR-AtTAS1c-D2-B/c	–	Kanamycin	–	Donor	*pENTR*	–	–	–
pMDC32B-AtTAS1c-D2-B/c	syn-tasiRNA(s)	Kanamycin<br>Hygromycin	Hygromycin	–	*pMDC32*	*CaMV* 2x35S	*Nos*	*A. thaliana*<br>*N. benthamiana*
pMDC32B-AtTAS1c-D2-B/c-AtMIR173	syn-tasiRNA(s)<br>miR173	Kanamycin<br>Hygromycin	Hygromycin	–	*pMDC32*	*CaMV* 2x35S	*nos*	*A. thaliana*<br>*N. benthamiana*

Supplementary Table S1: Phenotypic penetrance of syn-tasiRNAs expressed in Arabidopsis Col-0 T1 transgenic plants

Construct	T1 analyzed	Phenotypic penetrance[a]
35S:syn-tasiR-GUS-D2&D3	39	0% FT<br>0% Trich
35S:syn-tasiR-FT-D2-Trich-D3	16	100% FT<br>71% Trich
35S:syn-tasiR-Trich-D2-FT-D3	18	100% FT<br>82% Trich
35S:syn-tasiR-FT-D3-Trich-D4	48	100% FT<br>61% Trich
35S:syn-tasiR-Trich-D3-FT-D4	34	100% FT<br>76% Trich

[a] The Ft phenotype was defined as a higher 'days to flowering' value when compared to the average 'days to flowering' value of the *35S:syn-tasiR-GUS-D2&D3* control set.
The Trich phenotype was defined as a higher number of trichomes when compared to transformants of the 3*5S:syn-tasiR-GUS-D2&D3* control set.

Supplementary Table S2: Phenotypic penetrance of syn-tasiRNAs expressed in *A. thaliana* Col-0 T1 transgenic plants

Construct	T1 analyzed	Phenotypic penetrance[a]
35S:AtTAS1c-GUS$_{Ath}$-D2	77	0%
35S:AtTAS1c-FT-D2	38	100%
35S:AtTAS1c-FT-D3	33	100%
35S:TAS1c-FT-D4	66	100%
35S:TAS1c FT-D5	40	100%

[a] The Ft phenotype was defined as a higher 'days to flowering' value when compared to the average 'days to flowering' value of the *35S:AtTAS1c-GUS$_{Ath}$-D2* control set.

Supplementary Table S3: Phenotypic penetrance of syn-tasiRNAs expressed in Arabidopsis Col-0 T1 transgenic seedlings.

Construct	T1 analyzed	Phenotypic penetrance[a]
35S:AtTAS1c-GUS$_{Ath}$-D2	147	0%
35S:AtTAS1c-CH42-D2	106	100%<br>1% weak<br>39% intermediate<br>60 % severe
35S:AtTAS1c-CH42-D3	126	96%<br>26% weak<br>38% intermediate<br>32% severe
35S:TAS1c-CH42-D4	171	90%<br>30% weak<br>43% intermediate<br>17% severe
35S:TAS1c CH42-D5	65	94%<br>29% weak<br>51% intermediate<br>14% severe

[a] Ch42 phenotype is scored in 10 days-old seedling and is considered 'weak', 'intermediate' or 'severe' if seedlings have >2 leaves, exactly 2 leaves or no leaves (only 2 cotyledons), respectively.

Supplementary Table S4. Summary of results obtained from symptom and DAS-ELISA analyses in *Nicotiana benthamiana* bioassays.

	Analysis at 10 dpi		Analysis at 20 dpi	
**Sample**	**Symptomatic plants/Total**	**DAS-ELISA positive/Total**	**Symptomatic plants/Total**	**DAS-ELISA positive/Total**
35S:GUS	0/6	0/6	0/6	0/6
35S:syn-tasiR-GUS + *35S:MIR173a* + TSWV	6/6	6/6	6/6	6/6
35S:syn-tasiR-TSWV-D2 + *35S:MIR173a* + TSWV	0/6	0/6	06	0/6
35S:syn-tasiR-TSWV-D3 + *35S:MIR173a* + TSWV	0/6	1/6	1/6	1/6
35S:syn-tasiR-TSWV-D4 + *35S:MIR173a* + TSWV	0/6	1/6	3/6	3/6
35S:syn-tasiR-TSWV-D5 + *35S:MIR173a* + TSWV	6/6	6/6	6/6	6/6

Supplementary Table S5: Phenotypic penetrance of syn-tasiRNAs expressed in Arabidopsis Col-0 T1 transgenic plants

Construct	T1 analyzed	Phenotypic penetrance[a]
35S:AtTAS1c-GUS$_{Ath}$-D2	48	0%
35S:AtTAS1c-FT-D2	59	100%
35S:AtTAS1c-FT-D2-1M	77	100%
35S:AtTAS1c-FT-D2-2M	79	97%
35S:AtTAS1c FT-D2-3M	45	89%
35S:AtTAS1c-FT-D2-4M	92	59%
35S:AtTAS1c FT-D2-5M	39	8%

[a] The Ft phenotype was defined as a higher 'days to flowering' value when compared to the average 'days to flowering' value of the *35S:AtTAS1c-GUS$_{Ath}$-D2* control set.

Supplementary Table S6. Summary of results obtained from symptom and DAS-ELISA analyses in *Nicotiana benthamiana* bioassays.

	Analysis at 10 dpi		Analysis at 20 dpi	
**Sample**	**Symptomatic plants/Total**	**DAS-ELISA positive/Total**	**Symptomatic plants/Total**	**DAS-ELISA positive/Total**
35S:GUS	0/6	0/6	0/6	0/6
35S:syn-tasiR-GUS + *35S:MIR173a* + TSWV	6/6	6/6	6/6	6/6
35S:syn-tasiR-TSWV-D2 + *35S:MIR173a* + TSWV	0/6	0/6	0/6	0/6
35S:syn-tasiR-TSWV-D2-2M + *35S:MIR173a* + TSWV	2/6	3/6	6/6	6/6
35S:syn-tasiR-TSWV-D2-3M + *35S:MIR173a* + TSWV	6/6	6/6	6/6	6/6
35S:syn-tasiR-TSWV-D2-4M + *35S:MIR173a* + TSWV	6/6	6/6	6/6	6/6
35S:syn-tasiR-TSWV-D2-5M + *35S:MIR173a* + TSWV	6/6	6/6	6/6	6/6

Supplementary Table S7. Name, sequence and use of DNA oligonucleotides used in this study.

Oligonucleotide	Sequence	Construct/Aim
AC-14	ATCGCGCGCGGTGTCATCTATGTTACTGAATTCAAGCTTGGCGTGCCTGCA	*pMDC32B-AtTAS1c-D2-B/c-MIR173* (Gibsoon assembly)
AC-15	GGAAACAGCTATGACCATGATTACGAATTCGAATTCAGTAACATAGATGACACCGCG	
AC-49	AGGACACAATCACGTCTTACA	Probe for syn-tasiR-TSWV/syn-tasiR-TSWV-1M detection
AC-55	AGGGGCCATGCTAATCTTCTC	Probe for snoU6 detection
AC-82	TTTATGCGCTTGCTGAGTTTCCCCC	*35S:syn-tasiR-GUS$_{Ath}$-D2*
AC-83	CCGAGGGGGAAACTCAGCAAGCGCA	
AC-86	TTTATTGGTTATAAAGGAAGAGGCC	*35S:syn-tasiR-FT-D2*
AC-87	CCGAGGCCTCTTCCTTTATAACCAA	
AC-88	TTTATCGGTGGATCTTAGAAAATTATTCTAAGTCCAACATAGCGTATTGGTTATAAAGGAAGAGGCC	*35S:syn-tasiR-FT-D4*
AC-89	CCGAGGCCTCTTCCTTTATAACCAATACGCTATGTTGGACTTAGAATAATTTTCTAAGATCCACCGA	
AC-90	TTTATCGGTGGATCTTAGAAAATTATTCTAAGTCCAACATAGCGTATTCTAAGTTCAACATATCGACTTGGTTATAAAGGAAGAGGCC	*35S:syn-tasiR-FT-D5*
AC-91	CCGAGGCCTCTTCCTTTATAACCAAGTCGATATGTTGAACTTAGAATACGCTATGTTGGACTTAGAATAATTTTCTAAGATCCACCGA	
AC-92	TTTATTAAGTGTCACGGAAATCCCT	*35S:syn-tasiR-CH42-D2*
AC-93	CCGAAGGGATTTCCGTGACACTTAA	
AC-94	TTTATCGGTGGATCTTAGAAAATTATTCTAAGTCCAACATAGCGTATTAAGTGTCACGGAAATCCCT	*35S:syn-tasiR-CH42-D4*
AC-95	CCGAAGGGATTTCCGTGACACTTAATACGCTATGTTGGACTTAGAATAATTTTCTAAGATCCACCGA	
AC-96	TTTATCGGTGGATCTTAGAAAATTATTCTAAGTCCAACATAGCGTATTCTAAGTTCAACATATCGACTTAAGTGTCACGGAAATCCCT	*35S:syn-tasiR-CH42-D5*
AC-97	CCGAAGGGATTTCCGTGACACTTAAGTCGATATGTTGAACTTAGAATACGCTATGTTGGACTTAGAATAATTTTCTAAGATCCACCGA	
AC-98	TTTATTGGTTATAAAGGAAGAGGCCTCCCATTCGATACTGCTCGCC	*35S:syn-tasiR-FT-D2-Trich-D3*
AC-99	CCGAGGCGAGCAGTATCGAATGGGAGGCCTCTTCCTTTATAACCAA	
AC-100	TTTATCCCATTCGATACTGCTCGCCTTGGTTATAAAGGAAGAGGCC	*35S:syn-tasiR-Trich-D2-FT-D3*
AC-101	CCGAGGCCTCTTCCTTTATAACCAAGGCGAGCAGTATCGAATGGGA	
AC-102	TTTATGTAAGACGTGATTGTGTCCT	*35S:syn-tasiR-TSWV-D2*
AC-103	CCGAAGGACACAATCACGTCTTACA	
AC-104	TTTATCGGTGGATCTTAGAAAATTATGTAAGACGTGATTGTGTCCT	*35S:syn-tasiR-TSWV-D3*
AC-105	CCGAAGGACACAATCACGTCTTACATAATTTTCTAAGATCCACCGA	
AC-106	TTTATCGGTGGATCTTAGAAAATTATTCTAAGTCCAACATAGCGTATGTAAGACGTGATTGTGTCCT	*35S:syn-tasiR-TSWV-D4*
AC-107	CCGAAGGACACAATCACGTCTTACATACGCTATGTTGGACTTAGAATAATTTTCTAAGATCCACCGA	

AC-108	TTTATCGGTGGATCTTAGAAAATTATTCTAAGTCCAACATAGCGTATTCTAAGTTCAACATATCGACTGTAAGACGTGATTGTGTCCT	*35S:syn-tasiR-TSWV-D5*
AC-109	CCGAAGGACACAATCACGTCTTACAGTCGATATGTTGAACTTAGAATACGCTATGTTGGACTTAGAATAATTTTCTAAGATCCACCGA	
AC-114	TTTATCGGTGGATCTTAGAAAATTATTGGTTATAAAGGAAGAGGCC	*35S:syn-tasiR-FT-D3*
AC-115	CCGAGGCCTCTTCCTTTATAACCAATAATTTTCTAAGATCCACCGA	
AC-116	TTTATCGGTGGATCTTAGAAAATTATTAAGTGTCACGGAAATCCCT	*35S:syn-tasiR-CH42-D3*
AC-117	CCGAAGGGATTTCCGTGACACTTAATAATTTTCTAAGATCCACCGA	
AC-124	TTTATTGGTTATAAAGGAAGAGGCG	*35S:syn-tasiR-FT-D2-1M*
AC-125	CCGACGCCTCTTCCTTTATAACCAA	
AC-126	TTTATTGGTTATAAAGGAAGAGGGG	*35S:syn-tasiR-FT-D2-2M*
AC-127	CCGACCCCTCTTCCTTTATAACCAA	
AC-128	TTTATTGGTTATAAAGGAAGAGCGG	*35S:syn-tasiR-FT-D2-3M*
AC-129	CCGACCGCTCTTCCTTTATAACCAA	
AC-130	TTTATTGGTTATAAAGGAAGACCGG	*35S:syn-tasiR-FT-D2-4M*
AC-131	CCGACCGGTCTTCCTTTATAACCAA	
AC-132	TTTATTGGTTATAAAGGAAGTCCGG	*35S:syn-tasiR-FT-D2-5M*
AC-133	CCGACCGGACTTCCTTTATAACCAA	
AC-138	TTTATGTAAGACGTGATTGTGTCGT	*35S:syn-tasiR-TSWV-D2-2M*
AC-139	CCGAACGACACAATCACGTCTTACA	
AC-140	TTTATGTAAGACGTGATTGTGTAGT	*35S:syn-tasiR-TSWV-D2-3M*
AC-141	CCGAACTACACAATCACGTCTTACA	
AC-142	TTTATGTAAGACGTGATTGTGAAGT	*35S:syn-tasiR-TSWV-D2-4M*
AC-143	CCGAACTTCACAATCACGTCTTACA	
AC-144	TTTATGTAAGACGTGATTGTCAAGT	*35S:syn-tasiR-TSWV-D2-5M*
AC-145	CCGAACTTGACAATCACGTCTTACA	
AC-156	GGCGAGCAGTATCGAATGGGA	syn-tasiR-Trich probe
AC-158	AGGGATTTCCGTGACACTTAA	Probe for syn-tasiR-Ch42 detection
AC-157	GGCCTCTTCCTTTATAACCAA	Probe for syn-tasiR-FT detection
AC-190	CGCCTCTTCCTTTATAACCAA	Probe for syn-tasiR-FT-1M detection
AC-191	CCCCTCTTCCTTTATAACCAA	Probe for syn-tasiR-FT-2M detection
AC-192	CCGCTCTTCCTTTATAACCAA	Probe for syn-tasiR-FT-3M detection
AC-193	CCGGTCTTCCTTTATAACCAA	Probe for syn-tasiR-FT-4M detection
AC-194	CCGGACTTCCTTTATAACCAA	Probe for syn-tasiR-FT-5M detection
AC-197	ACGACACAATCACGTCTTACA	Probe for syn-tasiR-TSWV-2M detection
AC-198	ACTACACAATCACGTCTTACA	Probe for syn-tasiR-TSWV-3M detection
AC-199	ACTTCACAATCACGTCTTACA	Probe for syn-tasiR-TSWV-4M detection
AC-200	ACTTGACAATCACGTCTTACA	Probe for syn-tasiR-TSWV-5M detection
AC-211	TTTATATTGACCCACACTTTGCCGA	*35S:syn-tasiR-GUS$_{Sly}$-D2*
AC-212	CCGATCGGCAAAGTGTGGGTCAATA	
AC-213	TTTATGCGCTTGCTGAGTTTCCCCCTGCGCTTGCTGAGTTTCCCCC	

AC-214	CCGAGGGGGAAACTCAGCAAGCGCAGGGGGAAACTCAGCAAGCGCA	*35S:syn-tasiR-GUS*$_{Ath}$*-D2&D3*
AC-267	GCGGGAAGTCCACCACGGTTA	Probe for syn-tasiR-Su detection
AC-278	AAAAAGTCAACAAAACTTAAAGCGGCGGACTCATCGTAATCTCA	*Bsa*I mutagenesis in *MIR173*
AC-284	TATGCTTTGTTCAGATTCGAATCTGACGGACTCCAAATATTTTTC	
AC-288	TTTATAACCGTGGTGGACTTCCCGC	*35S:syn-tasiR-Su, 35S:syn-tasiR-Su/MIR173*
AC-289	CCGAGCGGGAAGTCCACCACGGTTA	
AC-333	TTTATCTTGTAACGCGCTTTCCCAG	*35S:syn-tasiR-GUS*$_{Nbe}$*-D2*
AC-334	CCGACTGGGAAAGCGCGTTACAAGA	
D-2042	GGCGGGTCTCATCGGTGGATCTTAGAAAATTATTCT	*pENTR-AtTAS1c-B/c-D2*
D-2043	GGCGGGTCTCTTAAATGGTCTATTCGCTTGTAGAGA	
D-2698	GTGATTTCTCTCTGCAAGCGAA	Probe for miR173 detection

Supplementary Text S1. DNA sequence in FASTA format of all *AtTAS1c*-based constructs used to express and analyze syn-tasiRNAs. Sequence corresponding to syn-tasiRNA-1 (position 3'D3[+]) and syn-tasiRNA-2 (position 3'D4[+]) is highlighted in blue and green, respectively. Sequence corresponding miR173 target site, and to tasiRNA D2, D3 and D4 are highlighted in blue, green, dark red and light pink, respectively. All the other sequences from Arabidopsis *TAS1c* gene are highlighted in black.

>AtTAS1c

```
AAACCTAAACCTAAACGGCTAAGCCCGACGTCAAATACCAAAAAGAGAAAAACAAGAGCGCCGTCAAGCTC
TGCAAATACGATCTGTAAGTCCATCTTAACACAAAAGTGAGATGGGTTCTTAGATCATGTTCCGCCGTTAG
ATCGAGTCATGGTCTTGTCTCATAGAAAGGTACTTTCGTTTACTTCTTTTGAGTATCGAGTAGAGCGTCGT
CTATAGTTAGTTTGAGATTGCGTTTGTCAGAAGTTAGGTTCAATGTCCCGGTCCAATTTTCACCAGCCATG
TGTCAGTTTCGTTCCTTCCCGTCCTCTTCTTTGATTTCGTTGGGTTACGGATGTTTTCGAGATGAAACAGC
ATTGTTTTGTTGTGATTTTTCTCTACAAGCGAATAGACCATTTATCGGTGGATCTTAGAAAATTATTCTAA
GTCCAACATAGCGTATTCTAAGTTCAACATATCGACGAACTAGAAAAGACATTGGACATATTCCAGGATAT
GCAAAAGAAAACAATGAATATTGTTTTGAATGTGTTCAAGTAAATGAGATTTTCAAGTCGTCTAAAGAACA
GTTGCTAATACAGTTACTTATTTCAATAAATAATTGGTTCTAATAATACAAAACATATTCGAGGATATGCA
GAAAAAAAGATGTTTGTTATTTTGAAAAGCTTGAGTAGTTTCTCTCCGAGGTGTAGCGAAGAAGCATCATC
TACTTTGTAATGTAATTTTCTTTATGTTTTCACTTTGTAATTTTATTTGTGTTAATGTACCATGGCCGATA
TCGGTTTTATTGAAAGAAAATTTATGTTACTTCTGTTTTGGCTTTGCAATCAGTTATGCTAGTTTTCTTAT
ACCCTTTCGTAAGCTTCCTAAGGAATCGTTCATTGATTTCCACTGCTTCATTGTATATTAAAACTTTACAA
CTGTATCGACCATCATATAATTCTGGGTCAAGAGATGAAAATAGAACACCACATCGTAAAGTGAAAT
GTGATTTTTCTCTACAAGCGAA miR173 target site
TCGGTGGATCTTAGAAAATTA tasiRNA D2
TTCTAAGTCCAACATAGCGTA tasiRNA D3
TTCTAAGTTCAACATATCGAC tasiRNA D4
```

>AtTAS1c-FT-D2-Trich-D3

```
AAACCTAAACCTAAACGGCTAAGCCCGACGTCAAATACCAAAAAGAGAAAAACAAGAGCGCCGTCAAGCTC
TGCAAATACGATCTGTAAGTCCATCTTAACACAAAAGTGAGATGGGTTCTTAGATCATGTTCCGCCGTTAG
ATCGAGTCATGGTCTTGTCTCATAGAAAGGTACTTTCGTTTACTTCTTTTGAGTATCGAGTAGAGCGTCGT
CTATAGTTAGTTTGAGATTGCGTTTGTCAGAAGTTAGGTTCAATGTCCCGGTCCAATTTTCACCAGCCATG
TGTCAGTTTCGTTCCTTCCCGTCCTCTTCTTTGATTTCGTTGGGTTACGGATGTTTTCGAGATGAAACAGC
ATTGTTTTGTTGTGATTTTTCTCTACAAGCGAATAGACCATTTATTGGTTATAAAGGAAGAGGCCTCCCAT
TCGATACTGCTCGCCTCGGTGGATCTTAGAAAATTATTCTAAGTCCAACATAGCGTATTCTAAGTTCAACA
TATCGACGAACTAGAAAAGACATTGGACATATTCCAGGATATGCAAAAGAAAACAATGAATATTGTTTTGA
ATGTGTTCAAGTAAATGAGATTTTCAAGTCGTCTAAAGAACAGTTGCTAATACAGTTACTTATTTCAATAA
ATAATTGGTTCTAATAATACAAAACATATTCGAGGATATGCAGAAAAAAAGATGTTTGTTATTTTGAAAAG
CTTGAGTAGTTTCTCTCCGAGGTGTAGCGAAGAAGCATCATCTACTTTGTAATGTAATTTTCTTTATGTTT
TCACTTTGTAATTTTATTTGTGTTAATGTACCATGGCCGATATCGGTTTTATTGAAAGAAAATTTATGTTA
CTTCTGTTTTGGCTTTGCAATCAGTTATGCTAGTTTTCTTATACCCTTTCGTAAGCTTCCTAAGGAATCGT
TCATTGATTTCCACTGCTTCATTGTATATTAAAACTTTACAACTGTATCGACCATCATATAATTCTGGGTC
AAGAGATGAAAATAGAACACCACATCGTAAAGTGAAAT
Syn-tasiR-FT: TTGGTTATAAAGGAAGAGGCC
Syn-tasiR-Trich: TCCCATTCGATACTGCTCGCC
```

>AtTAS1c-Trich-D2-FT-D3

AAACCTAAACCTAAACGGCTAAGCCCGACGTCAAATACCAAAAAGAGAAAAACAAGAGCGCCGTCAAGCTC
TGCAAATACGATCTGTAAGTCCATCTTAACACAAAAGTGAGATGGGTTCTTAGATCATGTTCCGCCGTTAG
ATCGAGTCATGGTCTTGTCTCATAGAAAGGTACTTTCGTTTACTTCTTTTGAGTATCGAGTAGAGCGTCGT
CTATAGTTAGTTTGAGATTGCGTTTGTCAGAAGTTAGGTTCAATGTCCCGGTCCAATTTTCACCAGCCATG
TGTCAGTTTCGTTCCTTCCCGTCCTCTTCTTTGATTTCGTTGGGTTACGGATGTTTTCGAGATGAAACAGC
ATTGTTTTGTTGTGATTTTTCTCTACAAGCGAATAGACCATTTATCCCATTCGATACTGCTCGCCTTGGTT
ATAAAGGAAGAGGCCTCGGTGGATCTTAGAAAATTATTCTAAGTCCAACATAGCGTATTCTAAGTTCAACA
TATCGACGAACTAGAAAAGACATTGGACATATTCCAGGATATGCAAAAGAAAACAATGAATATTGTTTTGA
ATGTGTTCAAGTAAATGAGATTTTCAAGTCGTCTAAAGAACAGTTGCTAATACAGTTACTTATTTCAATAA
ATAATTGGTTCTAATAATACAAAACATATTCGAGGATATGCAGAAAAAAAGATGTTTGTTATTTTGAAAAG
CTTGAGTAGTTTCTCTCCGAGGTGTAGCGAAGAAGCATCATCTACTTTGTAATGTAATTTTCTTTATGTTT
TCACTTTGTAATTTTATTTGTGTTAATGTACCATGGCCGATATCGGTTTTATTGAAAGAAAATTTATGTTA
CTTCTGTTTTGGCTTTGCAATCAGTTATGCTAGTTTTCTTATACCCTTTCGTAAGCTTCCTAAGGAATCGT
TCATTGATTTCCACTGCTTCATTGTATATTAAAACTTTACAACTGTATCGACCATCATATAATTCTGGGTC
AAGAGATGAAAATAGAACACCACATCGTAAAGTGAAAT

Syn-tasiR-Trich: TCCCATTCGATACTGCTCGCC
Syn-tasiR-FT: TTGGTTATAAAGGAAGAGGCC

>AtTAS1c-Trich-D3-FT-D4

AAACCTAAACCTAAACGGCTAAGCCCGACGTCAAATACCAAAAAGAGAAAAACAAGAGCGCCGTCAAGCTC
TGCAAATACGATCTGTAAGTCCATCTTAACACAAAAGTGAGATGGGTTCTTAGATCATGTTCCGCCGTTAG
ATCGAGTCATGGTCTTGTCTCATAGAAAGGTACTTTCGTTTACTTCTTTTGAGTATCGAGTAGAGCGTCGT
CTATAGTTAGTTTGAGATTGCGTTTGTCAGAAGTTAGGTTCAATGTCCCGGTCCAATTTTCACCAGCCATG
TGTCAGTTTCGTTCCTTCCCGTCCTCTTCTTTGATTTCGTTGGGTTACGGATGTTTTCGAGATGAAACAGC
ATTGTTTTGTTGTGATTTTTCTCTACAAGCGAATAGACCATTTATCGGTGGATCTTAGAAAATTATCCCAT
TCGATACTGCTCGCCTTGGTTATAAAGGAAGAGGCCGAACTAGAAAAGACATTGGACATATTCCAGGATAT
GCAAAAGAAAACAATGAATATTGTTTTGAATGTGTTCAAGTAAATGAGATTTTCAAGTCGTCTAAAGAACA
GTTGCTAATACAGTTACTTATTTCAATAAATAATTGGTTCTAATAATACAAAACATATTCGAGGATATGCA
GAAAAAAAGATGTTTGTTATTTTGAAAAGCTTGAGTAGTTTCTCTCCGAGGTGTAGCGAAGAAGCATCATC
TACTTTGTAATGTAATTTTCTTTATGTTTTCACTTTGTAATTTTATTTGTGTTAATGTACCATGGCCGATA
TCGGTTTTATTGAAAGAAAATTTATGTTACTTCTGTTTTGGCTTTGCAATCAGTTATGCTAGTTTTCTTAT
ACCCTTTCGTAAGCTTCCTAAGGAATCGTTCATTGATTTCCACTGCTTCATTGTATATTAAAACTTTACAA
CTGTATCGACCATCATATAATTCTGGGTCAAGAGATGAAAATAGAACACCACATCGTAAAGTGAAAT

Syn-tasiR-Trich: TCCCATTCGATACTGCTCGCC
Syn-tasiR-FT: TTGGTTATAAAGGAAGAGGCC

>AtTAS1c-Ft-D3-Trich-D4

AAACCTAAACCTAAACGGCTAAGCCCGACGTCAAATACCAAAAAGAGAAAAACAAGAGCGCCGTCAAGCTC
TGCAAATACGATCTGTAAGTCCATCTTAACACAAAAGTGAGATGGGTTCTTAGATCATGTTCCGCCGTTAG
ATCGAGTCATGGTCTTGTCTCATAGAAAGGTACTTTCGTTTACTTCTTTTGAGTATCGAGTAGAGCGTCGT
CTATAGTTAGTTTGAGATTGCGTTTGTCAGAAGTTAGGTTCAATGTCCCGGTCCAATTTTCACCAGCCATG
TGTCAGTTTCGTTCCTTCCCGTCCTCTTCTTTGATTTCGTTGGGTTACGGATGTTTTCGAGATGAAACAGC
ATTGTTTTGTTGTGATTTTTCTCTACAAGCGAATAGACCATTTATCGGTGGATCTTAGAAAATTATTGGTT
ATAAAGGAAGAGGCCTCCCATTCGATACTGCTCGCCGAACTAGAAAAGACATTGGACATATTCCAGGATAT
GCAAAAGAAAACAATGAATATTGTTTTGAATGTGTTCAAGTAAATGAGATTTTCAAGTCGTCTAAAGAACA
GTTGCTAATACAGTTACTTATTTCAATAAATAATTGGTTCTAATAATACAAAACATATTCGAGGATATGCA
GAAAAAAAGATGTTTGTTATTTTGAAAAGCTTGAGTAGTTTCTCTCCGAGGTGTAGCGAAGAAGCATCATC
TACTTTGTAATGTAATTTTCTTTATGTTTTCACTTTGTAATTTTATTTGTGTTAATGTACCATGGCCGATA
TCGGTTTTATTGAAAGAAAATTTATGTTACTTCTGTTTTGGCTTTGCAATCAGTTATGCTAGTTTTCTTAT
ACCCTTTCGTAAGCTTCCTAAGGAATCGTTCATTGATTTCCACTGCTTCATTGTATATTAAAACTTTACAA
CTGTATCGACCATCATATAATTCTGGGTCAAGAGATGAAAATAGAACACCACATCGTAAAGTGAAAT

Syn-tasiR-Trich: TCCCATTCGATACTGCTCGCC

Syn-tasiR-FT: TTGGTTATAAAGGAAGAGGCC

>*AtTAS1c-GUS*$_{Ath}$*-D2&D3*

```
AAACCTAAACCTAAACGGCTAAGCCCGACGTCAAATACCAAAAAGAGAAAAACAAGAGCGCCGTCAAGCTC
TGCAAATACGATCTGTAAGTCCATCTTAACACAAAAGTGAGATGGGTTCTTAGATCATGTTCCGCCGTTAG
ATCGAGTCATGGTCTTGTCTCATAGAAAGGTACTTTCGTTTACTTCTTTTGAGTATCGAGTAGAGCGTCGT
CTATAGTTAGTTTGAGATTGCGTTTGTCAGAAGTTAGGTTCAATGTCCCGGTCCAATTTTCACCAGCCATG
TGTCAGTTTCGTTCCTTCCCGTCCTCTTCTTTGATTTCGTTGGGTTACGGATGTTTTCGAGATGAAACAGC
ATTGTTTTGTTGTGATTTTTCTCTACAAGCGAATAGACCATTTATGCGCTTGCTGAGTTTCCCCCTGCGCT
TGCTGAGTTTCCCCCTCGGTGGATCTTAGAAAATTATTCTAAGTCCAACATAGCGTATTCTAAGTTCAACA
TATCGACGAACTAGAAAAGACATTGGACATATTCCAGGATATGCAAAAGAAAACAATGAATATTGTTTTGA
ATGTGTTCAAGTAAATGAGATTTTCAAGTCGTCTAAAGAACAGTTGCTAATACAGTTACTTATTTCAATAA
ATAATTGGTTCTAATAATACAAAACATATTCGAGGATATGCAGAAAAAAAGATGTTTGTTATTTTGAAAAG
CTTGAGTAGTTTCTCTCCGAGGTGTAGCGAAGAAGCATCATCTACTTTGTAATGTAATTTTCTTTATGTTT
TCACTTTGTAATTTTATTTGTGTTAATGTACCATGGCCGATATCGGTTTTATTGAAAGAAAATTTATGTTA
CTTCTGTTTTGGCTTTGCAATCAGTTATGCTAGTTTTCTTATACCCTTTCGTAAGCTTCCTAAGGAATCGT
TCATTGATTTCCACTGCTTCATTGTATATTAAAACTTTACAACTGTATCGACCATCATATAATTCTGGGTC
AAGAGATGAAAATAGAACACCACATCGTAAAGTGAAAT
```

Syn-tasiR-GUS$_{Ath}$: TGCGCTTGCTGAGTTTCCCCC

>*AtTAS1c-FT-D2*

```
AAACCTAAACCTAAACGGCTAAGCCCGACGTCAAATACCAAAAAGAGAAAAACAAGAGCGCCGTCAAGCTC
TGCAAATACGATCTGTAAGTCCATCTTAACACAAAAGTGAGATGGGTTCTTAGATCATGTTCCGCCGTTAG
ATCGAGTCATGGTCTTGTCTCATAGAAAGGTACTTTCGTTTACTTCTTTTGAGTATCGAGTAGAGCGTCGT
CTATAGTTAGTTTGAGATTGCGTTTGTCAGAAGTTAGGTTCAATGTCCCGGTCCAATTTTCACCAGCCATG
TGTCAGTTTCGTTCCTTCCCGTCCTCTTCTTTGATTTCGTTGGGTTACGGATGTTTTCGAGATGAAACAGC
ATTGTTTTGTTGTGATTTTTCTCTACAAGCGAATAGACCATTTATTGGTTATAAAGGAAGAGGCCTCGGTG
GATCTTAGAAAATTATTCTAAGTCCAACATAGCGTATTCTAAGTTCAACATATCGACGAACTAGAAAAGAC
ATTGGACATATTCCAGGATATGCAAAAGAAAACAATGAATATTGTTTTGAATGTGTTCAAGTAAATGAGAT
TTTCAAGTCGTCTAAAGAACAGTTGCTAATACAGTTACTTATTTCAATAAATAATTGGTTCTAATAATACA
AAACATATTCGAGGATATGCAGAAAAAAAGATGTTTGTTATTTTGAAAAGCTTGAGTAGTTTCTCTCCGAG
GTGTAGCGAAGAAGCATCATCTACTTTGTAATGTAATTTTCTTTATGTTTTCACTTTGTAATTTTATTTGT
GTTAATGTACCATGGCCGATATCGGTTTTATTGAAAGAAAATTTATGTTACTTCTGTTTTGGCTTTGCAAT
CAGTTATGCTAGTTTTCTTATACCCTTTCGTAAGCTTCCTAAGGAATCGTTCATTGATTTCCACTGCTTCA
TTGTATATTAAAACTTTACAACTGTATCGACCATCATATAATTCTGGGTCAAGAGATGAAAATAGAACACC
ACATCGTAAAGTGAAAT
```

Syn-tasiR-FT: TTGGTTATAAAGGAAGAGGCC

>*AtTAS1c-FT-D3*

```
AAACCTAAACCTAAACGGCTAAGCCCGACGTCAAATACCAAAAAGAGAAAAACAAGAGCGCCGTCAAGCTC
TGCAAATACGATCTGTAAGTCCATCTTAACACAAAAGTGAGATGGGTTCTTAGATCATGTTCCGCCGTTAG
ATCGAGTCATGGTCTTGTCTCATAGAAAGGTACTTTCGTTTACTTCTTTTGAGTATCGAGTAGAGCGTCGT
CTATAGTTAGTTTGAGATTGCGTTTGTCAGAAGTTAGGTTCAATGTCCCGGTCCAATTTTCACCAGCCATG
TGTCAGTTTCGTTCCTTCCCGTCCTCTTCTTTGATTTCGTTGGGTTACGGATGTTTTCGAGATGAAACAGC
ATTGTTTTGTTGTGATTTTTCTCTACAAGCGAATAGACCATTTATCGGTGGATCTTAGAAAATTATTGGTT
ATAAAGGAAGAGGCCTCGGTGGATCTTAGAAAATTATTCTAAGTCCAACATAGCGTATTCTAAGTTCAACA
TATCGACGAACTAGAAAAGACATTGGACATATTCCAGGATATGCAAAAGAAAACAATGAATATTGTTTTGA
ATGTGTTCAAGTAAATGAGATTTTCAAGTCGTCTAAAGAACAGTTGCTAATACAGTTACTTATTTCAATAA
ATAATTGGTTCTAATAATACAAAACATATTCGAGGATATGCAGAAAAAAAGATGTTTGTTATTTTGAAAAG
CTTGAGTAGTTTCTCTCCGAGGTGTAGCGAAGAAGCATCATCTACTTTGTAATGTAATTTTCTTTATGTTT
TCACTTTGTAATTTTATTTGTGTTAATGTACCATGGCCGATATCGGTTTTATTGAAAGAAAATTTATGTTA
CTTCTGTTTTGGCTTTGCAATCAGTTATGCTAGTTTTCTTATACCCTTTCGTAAGCTTCCTAAGGAATCGT
```

TCATTGATTTCCACTGCTTCATTGTATATTAAAACTTTACAACTGTATCGACCATCATATAATTCTGGGTC
AAGAGATGAAAATAGAACACCACATCGTAAAGTGAAAT

Syn-tasiR-FT: TTGGTTATAAAGGAAGAGGCC

>AtTAS1c-FT-D4

AAACCTAAACCTAAACGGCTAAGCCCGACGTCAAATACCAAAAAGAGAAAAACAAGAGCGCCGTCAAGCTC
TGCAAATACGATCTGTAAGTCCATCTTAACACAAAAGTGAGATGGGTTCTTAGATCATGTTCCGCCGTTAG
ATCGAGTCATGGTCTTGTCTCATAGAAAGGTACTTTCGTTTACTTCTTTTGAGTATCGAGTAGAGCGTCGT
CTATAGTTAGTTTGAGATTGCGTTTGTCAGAAGTTAGGTTCAATGTCCCGGTCCAATTTTCACCAGCCATG
TGTCAGTTTCGTTCCTTCCCGTCCTCTTCTTTGATTTCGTTGGGTTACGGATGTTTTCGAGATGAAACAGC
ATTGTTTTGTTGTGATTTTTCTCTACAAGCGAATAGACCATTTATCGGTGGATCTTAGAAAATTATTCTAA
GTCCAACATAGCGTATTGGTTATAAAGGAAGAGGCCTCGGTGGATCTTAGAAAATTATTCTAAGTCCAACA
TAGCGTATTCTAAGTTCAACATATCGACGAACTAGAAAAGACATTGGACATATTCCAGGATATGCAAAAGA
AAACAATGAATATTGTTTTGAATGTGTTCAAGTAAATGAGATTTTCAAGTCGTCTAAAGAACAGTTGCTAA
TACAGTTACTTATTTCAATAAATAATTGGTTCTAATAATACAAAACATATTCGAGGATATGCAGAAAAAAA
GATGTTTGTTATTTTGAAAAGCTTGAGTAGTTTCTCTCCGAGGTGTAGCGAAGAAGCATCATCTACTTTGT
AATGTAATTTTCTTTATGTTTTCACTTTGTAATTTTATTTGTGTTAATGTACCATGGCCGATATCGGTTTT
ATTGAAAGAAAATTTATGTTACTTCTGTTTTGGCTTTGCAATCAGTTATGCTAGTTTTCTTATACCCTTTC
GTAAGCTTCCTAAGGAATCGTTCATTGATTTCCACTGCTTCATTGTATATTAAAACTTTACAACTGTATCG
ACCATCATATAATTCTGGGTCAAGAGATGAAAATAGAACACCACATCGTAAAGTGAAAT

Syn-tasiR-FT: TTGGTTATAAAGGAAGAGGCC

>AtTAS1c-FT-D5

AAACCTAAACCTAAACGGCTAAGCCCGACGTCAAATACCAAAAAGAGAAAAACAAGAGCGCCGTCAAGCTC
TGCAAATACGATCTGTAAGTCCATCTTAACACAAAAGTGAGATGGGTTCTTAGATCATGTTCCGCCGTTAG
ATCGAGTCATGGTCTTGTCTCATAGAAAGGTACTTTCGTTTACTTCTTTTGAGTATCGAGTAGAGCGTCGT
CTATAGTTAGTTTGAGATTGCGTTTGTCAGAAGTTAGGTTCAATGTCCCGGTCCAATTTTCACCAGCCATG
TGTCAGTTTCGTTCCTTCCCGTCCTCTTCTTTGATTTCGTTGGGTTACGGATGTTTTCGAGATGAAACAGC
ATTGTTTTGTTGTGATTTTTCTCTACAAGCGAATAGACCATTTATCGGTGGATCTTAGAAAATTATTCTAA
GTCCAACATAGCGTATTCTAAGTTCAACATATCGACTTGGTTATAAAGGAAGAGGCCTCGGTGGATCTTAG
AAAATTATTCTAAGTCCAACATAGCGTATTCTAAGTTCAACATATCGACGAACTAGAAAAGACATTGGACA
TATTCCAGGATATGCAAAAGAAAACAATGAATATTGTTTTGAATGTGTTCAAGTAAATGAGATTTTCAAGT
CGTCTAAAGAACAGTTGCTAATACAGTTACTTATTTCAATAAATAATTGGTTCTAATAATACAAAACATAT
TCGAGGATATGCAGAAAAAAAGATGTTTGTTATTTTGAAAAGCTTGAGTAGTTTCTCTCCGAGGTGTAGCG
AAGAAGCATCATCTACTTTGTAATGTAATTTTCTTTATGTTTTCACTTTGTAATTTTATTTGTGTTAATGT
ACCATGGCCGATATCGGTTTTATTGAAAGAAAATTTATGTTACTTCTGTTTTGGCTTTGCAATCAGTTATG
CTAGTTTTCTTATACCCTTTCGTAAGCTTCCTAAGGAATCGTTCATTGATTTCCACTGCTTCATTGTATAT
TAAAACTTTACAACTGTATCGACCATCATATAATTCTGGGTCAAGAGATGAAAATAGAACACCACATCGTA
AAGTGAAAT

Syn-tasiR-FT: TTGGTTATAAAGGAAGAGGCC

>AtTAS1c-CH42-D2

AAACCTAAACCTAAACGGCTAAGCCCGACGTCAAATACCAAAAAGAGAAAAACAAGAGCGCCGTCAAGCTC
TGCAAATACGATCTGTAAGTCCATCTTAACACAAAAGTGAGATGGGTTCTTAGATCATGTTCCGCCGTTAG
ATCGAGTCATGGTCTTGTCTCATAGAAAGGTACTTTCGTTTACTTCTTTTGAGTATCGAGTAGAGCGTCGT
CTATAGTTAGTTTGAGATTGCGTTTGTCAGAAGTTAGGTTCAATGTCCCGGTCCAATTTTCACCAGCCATG
TGTCAGTTTCGTTCCTTCCCGTCCTCTTCTTTGATTTCGTTGGGTTACGGATGTTTTCGAGATGAAACAGC
ATTGTTTTGTTGTGATTTTTCTCTACAAGCGAATAGACCATTTATTAAGTGTCACGGAAATCCCTTCGGTG
GATCTTAGAAAATTATTCTAAGTCCAACATAGCGTATTCTAAGTTCAACATATCGACGAACTAGAAAAGAC
ATTGGACATATTCCAGGATATGCAAAAGAAAACAATGAATATTGTTTTGAATGTGTTCAAGTAAATGAGAT
TTTCAAGTCGTCTAAAGAACAGTTGCTAATACAGTTACTTATTTCAATAAATAATTGGTTCTAATAATACA
AAACATATTCGAGGATATGCAGAAAAAAAGATGTTTGTTATTTTGAAAAGCTTGAGTAGTTTCTCTCCGAG

GTGTAGCGAAGAAGCATCATCTACTTTGTAATGTAATTTTCTTTATGTTTTCACTTTGTAATTTTATTTGT
GTTAATGTACCATGGCCGATATCGGTTTTATTGAAAGAAAATTTATGTTACTTCTGTTTTGGCTTTGCAAT
CAGTTATGCTAGTTTTCTTATACCCTTTCGTAAGCTTCCTAAGGAATCGTTCATTGATTTCCACTGCTTCA
TTGTATATTAAAACTTTACAACTGTATCGACCATCATATAATTCTGGGTCAAGAGATGAAAATAGAACACC
ACATCGTAAAGTGAAAT

Syn-tasiR-AtCH42: TTAAGTGTCACGGAAATCCCT

>AtTAS1c-CH42-D3

AAACCTAAACCTAAACGGCTAAGCCCGACGTCAAATACCAAAAAGAGAAAAACAAGAGCGCCGTCAAGCTC
TGCAAATACGATCTGTAAGTCCATCTTAACACAAAAGTGAGATGGGTTCTTAGATCATGTTCCGCCGTTAG
ATCGAGTCATGGTCTTGTCTCATAGAAAGGTACTTTCGTTTACTTCTTTTGAGTATCGAGTAGAGCGTCGT
CTATAGTTAGTTTGAGATTGCGTTTGTCAGAAGTTAGGTTCAATGTCCCGGTCCAATTTTCACCAGCCATG
TGTCAGTTTCGTTCCTTCCCGTCCTCTTCTTTGATTTCGTTGGGTTACGGATGTTTTCGAGATGAAACAGC
ATTGTTTTGTTGTGATTTTTCTCTACAAGCGAATAGACCATTTATCGGTGGATCTTAGAAAATTATTAAGT
GTCACGGAAATCCCTTCGGTGGATCTTAGAAAATTATTCTAAGTCCAACATAGCGTATTCTAAGTTCAACA
TATCGACGAACTAGAAAAGACATTGGACATATTCCAGGATATGCAAAAGAAAACAATGAATATTGTTTTGA
ATGTGTTCAAGTAAATGAGATTTTCAAGTCGTCTAAAGAACAGTTGCTAATACAGTTACTTATTTCAATAA
ATAATTGGTTCTAATAATACAAAACATATTCGAGGATATGCAGAAAAAAAGATGTTTGTTATTTTGAAAAG
CTTGAGTAGTTTCTCTCCGAGGTGTAGCGAAGAAGCATCATCTACTTTGTAATGTAATTTTCTTTATGTTT
TCACTTTGTAATTTTATTTGTGTTAATGTACCATGGCCGATATCGGTTTTATTGAAAGAAAATTTATGTTA
CTTCTGTTTTGGCTTTGCAATCAGTTATGCTAGTTTTCTTATACCCTTTCGTAAGCTTCCTAAGGAATCGT
TCATTGATTTCCACTGCTTCATTGTATATTAAAACTTTACAACTGTATCGACCATCATATAATTCTGGGTC
AAGAGATGAAAATAGAACACCACATCGTAAAGTGAAAT

Syn-tasiR-CH42: TTAAGTGTCACGGAAATCCCT

>AtTAS1c-CH42-D4

AAACCTAAACCTAAACGGCTAAGCCCGACGTCAAATACCAAAAAGAGAAAAACAAGAGCGCCGTCAAGCTC
TGCAAATACGATCTGTAAGTCCATCTTAACACAAAAGTGAGATGGGTTCTTAGATCATGTTCCGCCGTTAG
ATCGAGTCATGGTCTTGTCTCATAGAAAGGTACTTTCGTTTACTTCTTTTGAGTATCGAGTAGAGCGTCGT
CTATAGTTAGTTTGAGATTGCGTTTGTCAGAAGTTAGGTTCAATGTCCCGGTCCAATTTTCACCAGCCATG
TGTCAGTTTCGTTCCTTCCCGTCCTCTTCTTTGATTTCGTTGGGTTACGGATGTTTTCGAGATGAAACAGC
ATTGTTTTGTTGTGATTTTTCTCTACAAGCGAATAGACCATTTATCGGTGGATCTTAGAAAATTATTCTAA
GTCCAACATAGCGTATTAAGTGTCACGGAAATCCCTTCGGTGGATCTTAGAAAATTATTCTAAGTCCAACA
TAGCGTATTCTAAGTTCAACATATCGACGAACTAGAAAAGACATTGGACATATTCCAGGATATGCAAAAGA
AAACAATGAATATTGTTTTGAATGTGTTCAAGTAAATGAGATTTTCAAGTCGTCTAAAGAACAGTTGCTAA
TACAGTTACTTATTTCAATAAATAATTGGTTCTAATAATACAAAACATATTCGAGGATATGCAGAAAAAAA
GATGTTTGTTATTTTGAAAAGCTTGAGTAGTTTCTCTCCGAGGTGTAGCGAAGAAGCATCATCTACTTTGT
AATGTAATTTTCTTTATGTTTTCACTTTGTAATTTTATTTGTGTTAATGTACCATGGCCGATATCGGTTTT
ATTGAAAGAAAATTTATGTTACTTCTGTTTTGGCTTTGCAATCAGTTATGCTAGTTTTCTTATACCCTTTC
GTAAGCTTCCTAAGGAATCGTTCATTGATTTCCACTGCTTCATTGTATATTAAAACTTTACAACTGTATCG
ACCATCATATAATTCTGGGTCAAGAGATGAAAATAGAACACCACATCGTAAAGTGAAAT

Syn-tasiR-CH42: TTAAGTGTCACGGAAATCCCT

>AtTAS1c-CH42-D5

AAACCTAAACCTAAACGGCTAAGCCCGACGTCAAATACCAAAAAGAGAAAAACAAGAGCGCCGTCAAGCTC
TGCAAATACGATCTGTAAGTCCATCTTAACACAAAAGTGAGATGGGTTCTTAGATCATGTTCCGCCGTTAG
ATCGAGTCATGGTCTTGTCTCATAGAAAGGTACTTTCGTTTACTTCTTTTGAGTATCGAGTAGAGCGTCGT
CTATAGTTAGTTTGAGATTGCGTTTGTCAGAAGTTAGGTTCAATGTCCCGGTCCAATTTTCACCAGCCATG
TGTCAGTTTCGTTCCTTCCCGTCCTCTTCTTTGATTTCGTTGGGTTACGGATGTTTTCGAGATGAAACAGC
ATTGTTTTGTTGTGATTTTTCTCTACAAGCGAATAGACCATTTATCGGTGGATCTTAGAAAATTATTCTAA
GTCCAACATAGCGTATTCTAAGTTCAACATATCGACTTAAGTGTCACGGAAATCCCTTCGGTGGATCTTAG
AAAATTATTCTAAGTCCAACATAGCGTATTCTAAGTTCAACATATCGACGAACTAGAAAAGACATTGGACA

TATTCCAGGATATGCAAAAGAAAACAATGAATATTGTTTTGAATGTGTTCAAGTAAATGAGATTTTCAAGT
CGTCTAAAGAACAGTTGCTAATACAGTTACTTATTTCAATAAATAATTGGTTCTAATAATACAAAACATAT
TCGAGGATATGCAGAAAAAAAGATGTTTGTTATTTTGAAAAGCTTGAGTAGTTTCTCTCCGAGGTGTAGCG
AAGAAGCATCATCTACTTTGTAATGTAATTTTCTTTATGTTTTCACTTTGTAATTTTATTTGTGTTAATGT
ACCATGGCCGATATCGGTTTTATTGAAAGAAAATTTATGTTACTTCTGTTTTGGCTTTGCAATCAGTTATG
CTAGTTTTCTTATACCCTTTCGTAAGCTTCCTAAGGAATCGTTCATTGATTTCCACTGCTTCATTGTATAT
TAAAACTTTACAACTGTATCGACCATCATATAATTCTGGGTCAAGAGATGAAAATAGAACACCACATCGTA
AAGTGAAAT

Syn-tasiR-CH42: TTAAGTGTCACGGAAATCCCT

>AtTAS1c-GUS$_{Sly}$-D2

AAACCTAAACCTAAACGGCTAAGCCCGACGTCAAATACCAAAAAGAGAAAAACAAGAGCGCCGTCAAGCTC
TGCAAATACGATCTGTAAGTCCATCTTAACACAAAAGTGAGATGGGTTCTTAGATCATGTTCCGCCGTTAG
ATCGAGTCATGGTCTTGTCTCATAGAAAGGTACTTTCGTTTACTTCTTTTGAGTATCGAGTAGAGCGTCGT
CTATAGTTAGTTTGAGATTGCGTTTGTCAGAAGTTAGGTTCAATGTCCCGGTCCAATTTTCACCAGCCATG
TGTCAGTTTCGTTCCTTCCCGTCCTCTTCTTTGATTTCGTTGGGTTACGGATGTTTTCGAGATGAAACAGC
ATTGTTTTGTTGTGATTTTTCTCTACAAGCGAATAGACCATTTATATTGACCCACACTTTGCCGATCGGTG
GATCTTAGAAAATTATTCTAAGTCCAACATAGCGTATTCTAAGTTCAACATATCGACGAACTAGAAAAGAC
ATTGGACATATTCCAGGATATGCAAAAGAAAACAATGAATATTGTTTTGAATGTGTTCAAGTAAATGAGAT
TTTCAAGTCGTCTAAAGAACAGTTGCTAATACAGTTACTTATTTCAATAAATAATTGGTTCTAATAATACA
AAACATATTCGAGGATATGCAGAAAAAAAGATGTTTGTTATTTTGAAAAGCTTGAGTAGTTTCTCTCCGAG
GTGTAGCGAAGAAGCATCATCTACTTTGTAATGTAATTTTCTTTATGTTTTCACTTTGTAATTTTATTTGT
GTTAATGTACCATGGCCGATATCGGTTTTATTGAAAGAAAATTTATGTTACTTCTGTTTTGGCTTTGCAAT
CAGTTATGCTAGTTTTCTTATACCCTTTCGTAAGCTTCCTAAGGAATCGTTCATTGATTTCCACTGCTTCA
TTGTATATTAAAACTTTACAACTGTATCGACCATCATATAATTCTGGGTCAAGAGATGAAAATAGAACACC
ACATCGTAAAGTGAAAT

Syn-tasiR-GUS$_{Sly}$: TATTGACCCACACTTTGCCGA

>AtTAS1c-TSWV-D2

AAACCTAAACCTAAACGGCTAAGCCCGACGTCAAATACCAAAAAGAGAAAAACAAGAGCGCCGTCAAGCTC
TGCAAATACGATCTGTAAGTCCATCTTAACACAAAAGTGAGATGGGTTCTTAGATCATGTTCCGCCGTTAG
ATCGAGTCATGGTCTTGTCTCATAGAAAGGTACTTTCGTTTACTTCTTTTGAGTATCGAGTAGAGCGTCGT
CTATAGTTAGTTTGAGATTGCGTTTGTCAGAAGTTAGGTTCAATGTCCCGGTCCAATTTTCACCAGCCATG
TGTCAGTTTCGTTCCTTCCCGTCCTCTTCTTTGATTTCGTTGGGTTACGGATGTTTTCGAGATGAAACAGC
ATTGTTTTGTTGTGATTTTTCTCTACAAGCGAATAGACCATTTATGTAAGACGTGATTGTGTCCTTCGGTG
GATCTTAGAAAATTATTCTAAGTCCAACATAGCGTATTCTAAGTTCAACATATCGACGAACTAGAAAAGAC
ATTGGACATATTCCAGGATATGCAAAAGAAAACAATGAATATTGTTTTGAATGTGTTCAAGTAAATGAGAT
TTTCAAGTCGTCTAAAGAACAGTTGCTAATACAGTTACTTATTTCAATAAATAATTGGTTCTAATAATACA
AAACATATTCGAGGATATGCAGAAAAAAAGATGTTTGTTATTTTGAAAAGCTTGAGTAGTTTCTCTCCGAG
GTGTAGCGAAGAAGCATCATCTACTTTGTAATGTAATTTTCTTTATGTTTTCACTTTGTAATTTTATTTGT
GTTAATGTACCATGGCCGATATCGGTTTTATTGAAAGAAAATTTATGTTACTTCTGTTTTGGCTTTGCAAT
CAGTTATGCTAGTTTTCTTATACCCTTTCGTAAGCTTCCTAAGGAATCGTTCATTGATTTCCACTGCTTCA
TTGTATATTAAAACTTTACAACTGTATCGACCATCATATAATTCTGGGTCAAGAGATGAAAATAGAACACC
ACATCGTAAAGTGAAAT

Syn-tasiR-TSWV: TGTAAGACGTGATTGTGTCCT

>AtTAS1c-TSWV-D3

AAACCTAAACCTAAACGGCTAAGCCCGACGTCAAATACCAAAAAGAGAAAAACAAGAGCGCCGTCAAGCTC
TGCAAATACGATCTGTAAGTCCATCTTAACACAAAAGTGAGATGGGTTCTTAGATCATGTTCCGCCGTTAG
ATCGAGTCATGGTCTTGTCTCATAGAAAGGTACTTTCGTTTACTTCTTTTGAGTATCGAGTAGAGCGTCGT
CTATAGTTAGTTTGAGATTGCGTTTGTCAGAAGTTAGGTTCAATGTCCCGGTCCAATTTTCACCAGCCATG
TGTCAGTTTCGTTCCTTCCCGTCCTCTTCTTTGATTTCGTTGGGTTACGGATGTTTTCGAGATGAAACAGC

ATTGTTTTGTTGTGATTTTTCTCTACAAGCGAATAGACCATTTATCGGTGGATCTTAGAAAATTATGTAAG
ACGTGATTGTGTCCTTCGGTGGATCTTAGAAAATTATTCTAAGTCCAACATAGCGTATTCTAAGTTCAACA
TATCGACGAACTAGAAAAGACATTGGACATATTCCAGGATATGCAAAAGAAAACAATGAATATTGTTTTGA
ATGTGTTCAAGTAAATGAGATTTTCAAGTCGTCTAAAGAACAGTTGCTAATACAGTTACTTATTTCAATAA
ATAATTGGTTCTAATAATACAAAACATATTCGAGGATATGCAGAAAAAAAGATGTTTGTTATTTTGAAAAG
CTTGAGTAGTTTCTCTCCGAGGTGTAGCGAAGAAGCATCATCTACTTTGTAATGTAATTTTCTTTATGTTT
TCACTTTGTAATTTTATTTGTGTTAATGTACCATGGCCGATATCGGTTTTATTGAAAGAAAATTTATGTTA
CTTCTGTTTTGGCTTTGCAATCAGTTATGCTAGTTTTCTTATACCCTTTCGTAAGCTTCCTAAGGAATCGT
TCATTGATTTCCACTGCTTCATTGTATATTAAAACTTTACAACTGTATCGACCATCATATAATTCTGGGTC
AAGAGATGAAAATAGAACACCACATCGTAAAGTGAAAT

Syn-tasiR-TSWV: TGTAAGACGTGATTGTGTCCT

>AtTAS1c-D4-TSWV

AAACCTAAACCTAAACGGCTAAGCCCGACGTCAAATACCAAAAAGAGAAAAACAAGAGCGCCGTCAAGCTC
TGCAAATACGATCTGTAAGTCCATCTTAACACAAAAGTGAGATGGGTTCTTAGATCATGTTCCGCCGTTAG
ATCGAGTCATGGTCTTGTCTCATAGAAAGGTACTTTCGTTTACTTCTTTTGAGTATCGAGTAGAGCGTCGT
CTATAGTTAGTTTGAGATTGCGTTTGTCAGAAGTTAGGTTCAATGTCCCGGTCCAATTTTCACCAGCCATG
TGTCAGTTTCGTTCCTTCCCGTCCTCTTCTTTGATTTCGTTGGGTTACGGATGTTTTCGAGATGAAACAGC
ATTGTTTTGTTGTGATTTTTCTCTACAAGCGAATAGACCATTTATCGGTGGATCTTAGAAAATTATTCTAA
GTCCAACATAGCGTATGTAAGACGTGATTGTGTCCTTCGGTGGATCTTAGAAAATTATTCTAAGTCCAACA
TAGCGTATTCTAAGTTCAACATATCGACGAACTAGAAAAGACATTGGACATATTCCAGGATATGCAAAAGA
AAACAATGAATATTGTTTTGAATGTGTTCAAGTAAATGAGATTTTCAAGTCGTCTAAAGAACAGTTGCTAA
TACAGTTACTTATTTCAATAAATAATTGGTTCTAATAATACAAAACATATTCGAGGATATGCAGAAAAAAA
GATGTTTGTTATTTTGAAAAGCTTGAGTAGTTTCTCTCCGAGGTGTAGCGAAGAAGCATCATCTACTTTGT
AATGTAATTTTCTTTATGTTTTCACTTTGTAATTTTATTTGTGTTAATGTACCATGGCCGATATCGGTTTT
ATTGAAAGAAAATTTATGTTACTTCTGTTTTGGCTTTGCAATCAGTTATGCTAGTTTTCTTATACCCTTTC
GTAAGCTTCCTAAGGAATCGTTCATTGATTTCCACTGCTTCATTGTATATTAAAACTTTACAACTGTATCG
ACCATCATATAATTCTGGGTCAAGAGATGAAAATAGAACACCACATCGTAAAGTGAAAT

Syn-tasiR-TSWV: TGTAAGACGTGATTGTGTCCT

>AtTAS1c-TSWV-D5

AAACCTAAACCTAAACGGCTAAGCCCGACGTCAAATACCAAAAAGAGAAAAACAAGAGCGCCGTCAAGCTC
TGCAAATACGATCTGTAAGTCCATCTTAACACAAAAGTGAGATGGGTTCTTAGATCATGTTCCGCCGTTAG
ATCGAGTCATGGTCTTGTCTCATAGAAAGGTACTTTCGTTTACTTCTTTTGAGTATCGAGTAGAGCGTCGT
CTATAGTTAGTTTGAGATTGCGTTTGTCAGAAGTTAGGTTCAATGTCCCGGTCCAATTTTCACCAGCCATG
TGTCAGTTTCGTTCCTTCCCGTCCTCTTCTTTGATTTCGTTGGGTTACGGATGTTTTCGAGATGAAACAGC
ATTGTTTTGTTGTGATTTTTCTCTACAAGCGAATAGACCATTTATCGGTGGATCTTAGAAAATTATTCTAA
GTCCAACATAGCGTATTCTAAGTTCAACATATCGACTGTAAGACGTGATTGTGTCCTTCGGTGGATCTTAG
AAAATTATTCTAAGTCCAACATAGCGTATTCTAAGTTCAACATATCGACGAACTAGAAAAGACATTGGACA
TATTCCAGGATATGCAAAAGAAAACAATGAATATTGTTTTGAATGTGTTCAAGTAAATGAGATTTTCAAGT
CGTCTAAAGAACAGTTGCTAATACAGTTACTTATTTCAATAAATAATTGGTTCTAATAATACAAAACATAT
TCGAGGATATGCAGAAAAAAAGATGTTTGTTATTTTGAAAAGCTTGAGTAGTTTCTCTCCGAGGTGTAGCG
AAGAAGCATCATCTACTTTGTAATGTAATTTTCTTTATGTTTTCACTTTGTAATTTTATTTGTGTTAATGT
ACCATGGCCGATATCGGTTTTATTGAAAGAAAATTTATGTTACTTCTGTTTTGGCTTTGCAATCAGTTATG
CTAGTTTTCTTATACCCTTTCGTAAGCTTCCTAAGGAATCGTTCATTGATTTCCACTGCTTCATTGTATAT
TAAAACTTTACAACTGTATCGACCATCATATAATTCTGGGTCAAGAGATGAAAATAGAACACCACATCGTA
AAGTGAAAT

Syn-tasiR- TSWV: TGTAAGACGTGATTGTGTCCT

>AtTAS1c-FT-D2-1M

AAACCTAAACCTAAACGGCTAAGCCCGACGTCAAATACCAAAAAGAGAAAAACAAGAGCGCCGTCAAGCTC
TGCAAATACGATCTGTAAGTCCATCTTAACACAAAAGTGAGATGGGTTCTTAGATCATGTTCCGCCGTTAG

ATCGAGTCATGGTCTTGTCTCATAGAAAGGTACTTTCGTTTACTTCTTTTGAGTATCGAGTAGAGCGTCGT
CTATAGTTAGTTTGAGATTGCGTTTGTCAGAAGTTAGGTTCAATGTCCCGGTCCAATTTTCACCAGCCATG
TGTCAGTTTCGTTCCTTCCCGTCCTCTTCTTTGATTTCGTTGGGTTACGGATGTTTTCGAGATGAAACAGC
ATTGTTTTGTTGTGATTTTTCTCTACAAGCGAATAGACCATTTATTGGTTATAAAGGAAGAGGCGTCGGTG
GATCTTAGAAAATTATTCTAAGTCCAACATAGCGTATTCTAAGTTCAACATATCGACGAACTAGAAAAGAC
ATTGGACATATTCCAGGATATGCAAAAGAAAACAATGAATATTGTTTTGAATGTGTTCAAGTAAATGAGAT
TTTCAAGTCGTCTAAAGAACAGTTGCTAATACAGTTACTTATTTCAATAAATAATTGGTTCTAATAATACA
AAACATATTCGAGGATATGCAGAAAAAAAGATGTTTGTTATTTTGAAAAGCTTGAGTAGTTTCTCTCCGAG
GTGTAGCGAAGAAGCATCATCTACTTTGTAATGTAATTTTCTTTATGTTTTCACTTTGTAATTTTATTTGT
GTTAATGTACCATGGCCGATATCGGTTTTATTGAAAGAAAATTTATGTTACTTCTGTTTTGGCTTTGCAAT
CAGTTATGCTAGTTTTCTTATACCCTTTCGTAAGCTTCCTAAGGAATCGTTCATTGATTTCCACTGCTTCA
TTGTATATTAAAACTTTACAACTGTATCGACCATCATATAATTCTGGGTCAAGAGATGAAAATAGAACACC
ACATCGTAAAGTGAAAT

Syn-tasiR-FT-1M: TTGGTTATAAAGGAAGAGGCG

>AtTAS1c-FT-D2-2M

AAACCTAAACCTAAACGGCTAAGCCCGACGTCAAATACCAAAAAGAGAAAAACAAGAGCGCCGTCAAGCTC
TGCAAATACGATCTGTAAGTCCATCTTAACACAAAAGTGAGATGGGTTCTTAGATCATGTTCCGCCGTTAG
ATCGAGTCATGGTCTTGTCTCATAGAAAGGTACTTTCGTTTACTTCTTTTGAGTATCGAGTAGAGCGTCGT
CTATAGTTAGTTTGAGATTGCGTTTGTCAGAAGTTAGGTTCAATGTCCCGGTCCAATTTTCACCAGCCATG
TGTCAGTTTCGTTCCTTCCCGTCCTCTTCTTTGATTTCGTTGGGTTACGGATGTTTTCGAGATGAAACAGC
ATTGTTTTGTTGTGATTTTTCTCTACAAGCGAATAGACCATTTATTGGTTATAAAGGAAGAGGGGTCGGTG
GATCTTAGAAAATTATTCTAAGTCCAACATAGCGTATTCTAAGTTCAACATATCGACGAACTAGAAAAGAC
ATTGGACATATTCCAGGATATGCAAAAGAAAACAATGAATATTGTTTTGAATGTGTTCAAGTAAATGAGAT
TTTCAAGTCGTCTAAAGAACAGTTGCTAATACAGTTACTTATTTCAATAAATAATTGGTTCTAATAATACA
AAACATATTCGAGGATATGCAGAAAAAAAGATGTTTGTTATTTTGAAAAGCTTGAGTAGTTTCTCTCCGAG
GTGTAGCGAAGAAGCATCATCTACTTTGTAATGTAATTTTCTTTATGTTTTCACTTTGTAATTTTATTTGT
GTTAATGTACCATGGCCGATATCGGTTTTATTGAAAGAAAATTTATGTTACTTCTGTTTTGGCTTTGCAAT
CAGTTATGCTAGTTTTCTTATACCCTTTCGTAAGCTTCCTAAGGAATCGTTCATTGATTTCCACTGCTTCA
TTGTATATTAAAACTTTACAACTGTATCGACCATCATATAATTCTGGGTCAAGAGATGAAAATAGAACACC
ACATCGTAAAGTGAAAT

Syn-tasiR-FT-2M: TTGGTTATAAAGGAAGAGGGG

>AtTAS1c-FT-D2-3M

AAACCTAAACCTAAACGGCTAAGCCCGACGTCAAATACCAAAAAGAGAAAAACAAGAGCGCCGTCAAGCTC
TGCAAATACGATCTGTAAGTCCATCTTAACACAAAAGTGAGATGGGTTCTTAGATCATGTTCCGCCGTTAG
ATCGAGTCATGGTCTTGTCTCATAGAAAGGTACTTTCGTTTACTTCTTTTGAGTATCGAGTAGAGCGTCGT
CTATAGTTAGTTTGAGATTGCGTTTGTCAGAAGTTAGGTTCAATGTCCCGGTCCAATTTTCACCAGCCATG
TGTCAGTTTCGTTCCTTCCCGTCCTCTTCTTTGATTTCGTTGGGTTACGGATGTTTTCGAGATGAAACAGC
ATTGTTTTGTTGTGATTTTTCTCTACAAGCGAATAGACCATTTATTGGTTATAAAGGAAGAGCGGTCGGTG
GATCTTAGAAAATTATTCTAAGTCCAACATAGCGTATTCTAAGTTCAACATATCGACGAACTAGAAAAGAC
ATTGGACATATTCCAGGATATGCAAAAGAAAACAATGAATATTGTTTTGAATGTGTTCAAGTAAATGAGAT
TTTCAAGTCGTCTAAAGAACAGTTGCTAATACAGTTACTTATTTCAATAAATAATTGGTTCTAATAATACA
AAACATATTCGAGGATATGCAGAAAAAAAGATGTTTGTTATTTTGAAAAGCTTGAGTAGTTTCTCTCCGAG
GTGTAGCGAAGAAGCATCATCTACTTTGTAATGTAATTTTCTTTATGTTTTCACTTTGTAATTTTATTTGT
GTTAATGTACCATGGCCGATATCGGTTTTATTGAAAGAAAATTTATGTTACTTCTGTTTTGGCTTTGCAAT
CAGTTATGCTAGTTTTCTTATACCCTTTCGTAAGCTTCCTAAGGAATCGTTCATTGATTTCCACTGCTTCA
TTGTATATTAAAACTTTACAACTGTATCGACCATCATATAATTCTGGGTCAAGAGATGAAAATAGAACACC
ACATCGTAAAGTGAAAT

Syn-tasiR-FT-3M: TTGGTTATAAAGGAAGAGCGG

>AtTAS1c-FT-D2-4M

AAACCTAAACCTAAACGGCTAAGCCCGACGTCAAATACCAAAAAGAGAAAAACAAGAGCGCCGTCAAGCTC
TGCAAATACGATCTGTAAGTCCATCTTAACACAAAAGTGAGATGGGTTCTTAGATCATGTTCCGCCGTTAG
ATCGAGTCATGGTCTTGTCTCATAGAAAGGTACTTTCGTTTACTTCTTTTGAGTATCGAGTAGAGCGTCGT
CTATAGTTAGTTTGAGATTGCGTTTGTCAGAAGTTAGGTTCAATGTCCCGGTCCAATTTTCACCAGCCATG
TGTCAGTTTCGTTCCTTCCCGTCCTCTTCTTTGATTTCGTTGGGTTACGGATGTTTTCGAGATGAAACAGC
ATTGTTTTGTTGTGATTTTTCTCTACAAGCGAATAGACCATTTATTGGTTATAAAGGAAGACCGGTCGGTG
GATCTTAGAAAATTATTCTAAGTCCAACATAGCGTATTCTAAGTTCAACATATCGACGAACTAGAAAAGAC
ATTGGACATATTCCAGGATATGCAAAAGAAAACAATGAATATTGTTTTGAATGTGTTCAAGTAAATGAGAT
TTTCAAGTCGTCTAAAGAACAGTTGCTAATACAGTTACTTATTTCAATAAATAATTGGTTCTAATAATACA
AAACATATTCGAGGATATGCAGAAAAAAAGATGTTTGTTATTTTGAAAAGCTTGAGTAGTTTCTCTCCGAG
GTGTAGCGAAGAAGCATCATCTACTTTGTAATGTAATTTTCTTTATGTTTTCACTTTGTAATTTTATTTGT
GTTAATGTACCATGGCCGATATCGGTTTTATTGAAAGAAAATTTATGTTACTTCTGTTTTGGCTTTGCAAT
CAGTTATGCTAGTTTTCTTATACCCTTTCGTAAGCTTCCTAAGGAATCGTTCATTGATTTCCACTGCTTCA
TTGTATATTAAAACTTTACAACTGTATCGACCATCATATAATTCTGGGTCAAGAGATGAAAATAGAACACC
ACATCGTAAAGTGAAAT
Syn-tasiR-FT-4M: TTGGTTATAAAGGAAGACCGG

>AtTAS1c-FT-D2-5M

AAACCTAAACCTAAACGGCTAAGCCCGACGTCAAATACCAAAAAGAGAAAAACAAGAGCGCCGTCAAGCTC
TGCAAATACGATCTGTAAGTCCATCTTAACACAAAAGTGAGATGGGTTCTTAGATCATGTTCCGCCGTTAG
ATCGAGTCATGGTCTTGTCTCATAGAAAGGTACTTTCGTTTACTTCTTTTGAGTATCGAGTAGAGCGTCGT
CTATAGTTAGTTTGAGATTGCGTTTGTCAGAAGTTAGGTTCAATGTCCCGGTCCAATTTTCACCAGCCATG
TGTCAGTTTCGTTCCTTCCCGTCCTCTTCTTTGATTTCGTTGGGTTACGGATGTTTTCGAGATGAAACAGC
ATTGTTTTGTTGTGATTTTTCTCTACAAGCGAATAGACCATTTATTGGTTATAAAGGAAGTCCGGTCGGTG
GATCTTAGAAAATTATTCTAAGTCCAACATAGCGTATTCTAAGTTCAACATATCGACGAACTAGAAAAGAC
ATTGGACATATTCCAGGATATGCAAAAGAAAACAATGAATATTGTTTTGAATGTGTTCAAGTAAATGAGAT
TTTCAAGTCGTCTAAAGAACAGTTGCTAATACAGTTACTTATTTCAATAAATAATTGGTTCTAATAATACA
AAACATATTCGAGGATATGCAGAAAAAAAGATGTTTGTTATTTTGAAAAGCTTGAGTAGTTTCTCTCCGAG
GTGTAGCGAAGAAGCATCATCTACTTTGTAATGTAATTTTCTTTATGTTTTCACTTTGTAATTTTATTTGT
GTTAATGTACCATGGCCGATATCGGTTTTATTGAAAGAAAATTTATGTTACTTCTGTTTTGGCTTTGCAAT
CAGTTATGCTAGTTTTCTTATACCCTTTCGTAAGCTTCCTAAGGAATCGTTCATTGATTTCCACTGCTTCA
TTGTATATTAAAACTTTACAACTGTATCGACCATCATATAATTCTGGGTCAAGAGATGAAAATAGAACACC
ACATCGTAAAGTGAAAT
Syn-tasiR-FT-5M: TTGGTTATAAAGGAAGTCCGG

>AtTAS1c-TSWV-D2-2M

AAACCTAAACCTAAACGGCTAAGCCCGACGTCAAATACCAAAAAGAGAAAAACAAGAGCGCCGTCAAGCTC
TGCAAATACGATCTGTAAGTCCATCTTAACACAAAAGTGAGATGGGTTCTTAGATCATGTTCCGCCGTTAG
ATCGAGTCATGGTCTTGTCTCATAGAAAGGTACTTTCGTTTACTTCTTTTGAGTATCGAGTAGAGCGTCGT
CTATAGTTAGTTTGAGATTGCGTTTGTCAGAAGTTAGGTTCAATGTCCCGGTCCAATTTTCACCAGCCATG
TGTCAGTTTCGTTCCTTCCCGTCCTCTTCTTTGATTTCGTTGGGTTACGGATGTTTTCGAGATGAAACAGC
ATTGTTTTGTTGTGATTTTTCTCTACAAGCGAATAGACCATTTATGTAAGACGTGATTGTGTCGTTCGGTG
GATCTTAGAAAATTATTCTAAGTCCAACATAGCGTATTCTAAGTTCAACATATCGACGAACTAGAAAAGAC
ATTGGACATATTCCAGGATATGCAAAAGAAAACAATGAATATTGTTTTGAATGTGTTCAAGTAAATGAGAT
TTTCAAGTCGTCTAAAGAACAGTTGCTAATACAGTTACTTATTTCAATAAATAATTGGTTCTAATAATACA
AAACATATTCGAGGATATGCAGAAAAAAAGATGTTTGTTATTTTGAAAAGCTTGAGTAGTTTCTCTCCGAG
GTGTAGCGAAGAAGCATCATCTACTTTGTAATGTAATTTTCTTTATGTTTTCACTTTGTAATTTTATTTGT
GTTAATGTACCATGGCCGATATCGGTTTTATTGAAAGAAAATTTATGTTACTTCTGTTTTGGCTTTGCAAT
CAGTTATGCTAGTTTTCTTATACCCTTTCGTAAGCTTCCTAAGGAATCGTTCATTGATTTCCACTGCTTCA
TTGTATATTAAAACTTTACAACTGTATCGACCATCATATAATTCTGGGTCAAGAGATGAAAATAGAACACC
ACATCGTAAAGTGAAAT
Syn-tasiR-TSWV-2M: TGTAAGACGTGATTGTGTCGT

>AtTAS1c-TSWV-D2-3M

```
AAACCTAAACCTAAACGGCTAAGCCCGACGTCAAATACCAAAAAGAGAAAAACAAGAGCGCCGTCAAGCTC
TGCAAATACGATCTGTAAGTCCATCTTAACACAAAAGTGAGATGGGTTCTTAGATCATGTTCCGCCGTTAG
ATCGAGTCATGGTCTTGTCTCATAGAAAGGTACTTTCGTTTACTTCTTTTGAGTATCGAGTAGAGCGTCGT
CTATAGTTAGTTTGAGATTGCGTTTGTCAGAAGTTAGGTTCAATGTCCCGGTCCAATTTTCACCAGCCATG
TGTCAGTTTCGTTCCTTCCCGTCCTCTTCTTTGATTTCGTTGGGTTACGGATGTTTTCGAGATGAAACAGC
ATTGTTTTGTTGTGATTTTTCTCTACAAGCGAATAGACCATTTATGTAAGACGTGATTGTGTAGTTCGGTG
GATCTTAGAAAATTATTCTAAGTCCAACATAGCGTATTCTAAGTTCAACATATCGACGAACTAGAAAAGAC
ATTGGACATATTCCAGGATATGCAAAAGAAAACAATGAATATTGTTTTGAATGTGTTCAAGTAAATGAGAT
TTTCAAGTCGTCTAAAGAACAGTTGCTAATACAGTTACTTATTTCAATAAATAATTGGTTCTAATAATACA
AAACATATTCGAGGATATGCAGAAAAAAAGATGTTTGTTATTTTGAAAAGCTTGAGTAGTTTCTCTCCGAG
GTGTAGCGAAGAAGCATCATCTACTTTGTAATGTAATTTTCTTTATGTTTTCACTTTGTAATTTTATTTGT
GTTAATGTACCATGGCCGATATCGGTTTTATTGAAAGAAAATTTATGTTACTTCTGTTTTGGCTTTGCAAT
CAGTTATGCTAGTTTTCTTATACCCTTTCGTAAGCTTCCTAAGGAATCGTTCATTGATTTCCACTGCTTCA
TTGTATATTAAAACTTTACAACTGTATCGACCATCATATAATTCTGGGTCAAGAGATGAAAATAGAACACC
ACATCGTAAAGTGAAAT
```

Syn-tasiR-TSWV-3M: TGTAAGACGTGATTGTGTAGT

>*AtTAS1c-TSWV-D2-4M*

```
AAACCTAAACCTAAACGGCTAAGCCCGACGTCAAATACCAAAAAGAGAAAAACAAGAGCGCCGTCAAGCTC
TGCAAATACGATCTGTAAGTCCATCTTAACACAAAAGTGAGATGGGTTCTTAGATCATGTTCCGCCGTTAG
ATCGAGTCATGGTCTTGTCTCATAGAAAGGTACTTTCGTTTACTTCTTTTGAGTATCGAGTAGAGCGTCGT
CTATAGTTAGTTTGAGATTGCGTTTGTCAGAAGTTAGGTTCAATGTCCCGGTCCAATTTTCACCAGCCATG
TGTCAGTTTCGTTCCTTCCCGTCCTCTTCTTTGATTTCGTTGGGTTACGGATGTTTTCGAGATGAAACAGC
ATTGTTTTGTTGTGATTTTTCTCTACAAGCGAATAGACCATTTATGTAAGACGTGATTGTGAAGTTCGGTG
GATCTTAGAAAATTATTCTAAGTCCAACATAGCGTATTCTAAGTTCAACATATCGACGAACTAGAAAAGAC
ATTGGACATATTCCAGGATATGCAAAAGAAAACAATGAATATTGTTTTGAATGTGTTCAAGTAAATGAGAT
TTTCAAGTCGTCTAAAGAACAGTTGCTAATACAGTTACTTATTTCAATAAATAATTGGTTCTAATAATACA
AAACATATTCGAGGATATGCAGAAAAAAAGATGTTTGTTATTTTGAAAAGCTTGAGTAGTTTCTCTCCGAG
GTGTAGCGAAGAAGCATCATCTACTTTGTAATGTAATTTTCTTTATGTTTTCACTTTGTAATTTTATTTGT
GTTAATGTACCATGGCCGATATCGGTTTTATTGAAAGAAAATTTATGTTACTTCTGTTTTGGCTTTGCAAT
CAGTTATGCTAGTTTTCTTATACCCTTTCGTAAGCTTCCTAAGGAATCGTTCATTGATTTCCACTGCTTCA
TTGTATATTAAAACTTTACAACTGTATCGACCATCATATAATTCTGGGTCAAGAGATGAAAATAGAACACC
ACATCGTAAAGTGAAAT
```

Syn-tasiR-TSWV-4M: TGTAAGACGTGATTGTGAAGT

>*AtTAS1c-TSWV-D2-5M*

```
AAACCTAAACCTAAACGGCTAAGCCCGACGTCAAATACCAAAAAGAGAAAAACAAGAGCGCCGTCAAGCTC
TGCAAATACGATCTGTAAGTCCATCTTAACACAAAAGTGAGATGGGTTCTTAGATCATGTTCCGCCGTTAG
ATCGAGTCATGGTCTTGTCTCATAGAAAGGTACTTTCGTTTACTTCTTTTGAGTATCGAGTAGAGCGTCGT
CTATAGTTAGTTTGAGATTGCGTTTGTCAGAAGTTAGGTTCAATGTCCCGGTCCAATTTTCACCAGCCATG
TGTCAGTTTCGTTCCTTCCCGTCCTCTTCTTTGATTTCGTTGGGTTACGGATGTTTTCGAGATGAAACAGC
ATTGTTTTGTTGTGATTTTTCTCTACAAGCGAATAGACCATTTATGTAAGACGTGATTGTCAAGTTCGGTG
GATCTTAGAAAATTATTCTAAGTCCAACATAGCGTATTCTAAGTTCAACATATCGACGAACTAGAAAAGAC
ATTGGACATATTCCAGGATATGCAAAAGAAAACAATGAATATTGTTTTGAATGTGTTCAAGTAAATGAGAT
TTTCAAGTCGTCTAAAGAACAGTTGCTAATACAGTTACTTATTTCAATAAATAATTGGTTCTAATAATACA
AAACATATTCGAGGATATGCAGAAAAAAAGATGTTTGTTATTTTGAAAAGCTTGAGTAGTTTCTCTCCGAG
GTGTAGCGAAGAAGCATCATCTACTTTGTAATGTAATTTTCTTTATGTTTTCACTTTGTAATTTTATTTGT
GTTAATGTACCATGGCCGATATCGGTTTTATTGAAAGAAAATTTATGTTACTTCTGTTTTGGCTTTGCAAT
CAGTTATGCTAGTTTTCTTATACCCTTTCGTAAGCTTCCTAAGGAATCGTTCATTGATTTCCACTGCTTCA
TTGTATATTAAAACTTTACAACTGTATCGACCATCATATAATTCTGGGTCAAGAGATGAAAATAGAACACC
ACATCGTAAAGTGAAAT
```

Syn-tasiR-TSWV-5M: TGTAAGACGTGATTGTCAAGT

>AtTAS1c-GUS$_{Nb}$-D2

AAACCTAAACCTAAACGGCTAAGCCCGACGTCAAATACCAAAAAGAGAAAAACAAGAGCGCCGTCAAGCTC
TGCAAATACGATCTGTAAGTCCATCTTAACACAAAAGTGAGATGGGTTCTTAGATCATGTTCCGCCGTTAG
ATCGAGTCATGGTCTTGTCTCATAGAAAGGTACTTTCGTTTACTTCTTTTGAGTATCGAGTAGAGCGTCGT
CTATAGTTAGTTTGAGATTGCGTTTGTCAGAAGTTAGGTTCAATGTCCCGGTCCAATTTTCACCAGCCATG
TGTCAGTTTCGTTCCTTCCCGTCCTCTTCTTTGATTTCGTTGGGTTACGGATGTTTTCGAGATGAAACAGC
ATTGTTTTGTTGTGATTTTTCTCTACAAGCGAATAGACCATTTATCTTGTAACGCGCTTTCCCAGTCGGTG
GATCTTAGAAAATTATTCTAAGTCCAACATAGCGTATTCTAAGTTCAACATATCGACGAACTAGAAAAGAC
ATTGGACATATTCCAGGATATGCAAAAGAAAACAATGAATATTGTTTTGAATGTGTTCAAGTAAATGAGAT
TTTCAAGTCGTCTAAAGAACAGTTGCTAATACAGTTACTTATTTCAATAAATAATTGGTTCTAATAATACA
AAACATATTCGAGGATATGCAGAAAAAAAGATGTTTGTTATTTTGAAAAGCTTGAGTAGTTTCTCTCCGAG
GTGTAGCGAAGAAGCATCATCTACTTTGTAATGTAATTTTCTTTATGTTTTCACTTTGTAATTTTATTTGT
GTTAATGTACCATGGCCGATATCGGTTTTATTGAAAGAAAATTTATGTTACTTCTGTTTTGGCTTTGCAAT
CAGTTATGCTAGTTTTCTTATACCCTTTCGTAAGCTTCCTAAGGAATCGTTCATTGATTTCCACTGCTTCA
TTGTATATTAAAACTTTACAACTGTATCGACCATCATATAATTCTGGGTCAAGAGATGAAAATAGAACACC
ACATCGTAAAGTGAAAT

Syn-tasiR-GUS$_{Nb}$: TCTTGTAACGCGCTTTCCCAG

>AtTAS1c-Su-D2

AAACCTAAACCTAAACGGCTAAGCCCGACGTCAAATACCAAAAAGAGAAAAACAAGAGCGCCGTCAAGCTC
TGCAAATACGATCTGTAAGTCCATCTTAACACAAAAGTGAGATGGGTTCTTAGATCATGTTCCGCCGTTAG
ATCGAGTCATGGTCTTGTCTCATAGAAAGGTACTTTCGTTTACTTCTTTTGAGTATCGAGTAGAGCGTCGT
CTATAGTTAGTTTGAGATTGCGTTTGTCAGAAGTTAGGTTCAATGTCCCGGTCCAATTTTCACCAGCCATG
TGTCAGTTTCGTTCCTTCCCGTCCTCTTCTTTGATTTCGTTGGGTTACGGATGTTTTCGAGATGAAACAGC
ATTGTTTTGTTGTGATTTTTCTCTACAAGCGAATAGACCATTTATAACCGTGGTGGACTTCCCGCTCGGTG
GATCTTAGAAAATTATTCTAAGTCCAACATAGCGTATTCTAAGTTCAACATATCGACGAACTAGAAAAGAC
ATTGGACATATTCCAGGATATGCAAAAGAAAACAATGAATATTGTTTTGAATGTGTTCAAGTAAATGAGAT
TTTCAAGTCGTCTAAAGAACAGTTGCTAATACAGTTACTTATTTCAATAAATAATTGGTTCTAATAATACA
AAACATATTCGAGGATATGCAGAAAAAAAGATGTTTGTTATTTTGAAAAGCTTGAGTAGTTTCTCTCCGAG
GTGTAGCGAAGAAGCATCATCTACTTTGTAATGTAATTTTCTTTATGTTTTCACTTTGTAATTTTATTTGT
GTTAATGTACCATGGCCGATATCGGTTTTATTGAAAGAAAATTTATGTTACTTCTGTTTTGGCTTTGCAAT
CAGTTATGCTAGTTTTCTTATACCCTTTCGTAAGCTTCCTAAGGAATCGTTCATTGATTTCCACTGCTTCA
TTGTATATTAAAACTTTACAACTGTATCGACCATCATATAATTCTGGGTCAAGAGATGAAAATAGAACACC
ACATCGTAAAGTGAAAT

Syn-tasiR-Su: TAACCGTGGTGGACTTCCCGC

Supplementary Text S2. DNA sequence of *Bsa*I-*ccd*B-based (B/c) vectors used for direct cloning of syn-tasiRNAs downstream of 3'D1[+] position in *AtTAS1c*.

***>pENTR-AtTAS1c-D2-B/c* (5031 bp)**

CTTTCCTGCGTTATCCCCTGATTCTGTGGATAACCGTATTACCGCCTTTGAGTGAGCTGATACCGCTCGC
CGCAGCCGAACGACCGAGCGCAGCGAGTCAGTGAGCGAGGAAGCGGAAGAGCGCCCAATACGCAAACCGC
CTCTCCCCGCGCGTTGGCCGATTCATTAATGCAGCTGGCACGACAGGTTTCCCGACTGGAAAGCGGGCAG
TGAGCGCAACGCAATTAATACGCGTACCGCTAGCCAGGAAGAGTTTGTAGAAACGCAAAAAGGCCATCCG
TCAGGATGGCCTTCTGCTTAGTTTGATGCCTGGCAGTTTATGGCGGGCGTCCTGCCCGCCACCCTCCGGG
CCGTTGCTTCACAACGTTCAAATCCGCTCCCGGCGGATTTGTCCTACTCAGGAGAGCGTTCACCGACAAA
CAACAGATAAAACGAAAGGCCCAGTCTTCCGACTGAGCCTTTCGTTTTATTTGATGCCTGGCAGTTCCCT
ACTCTCGCGTTAACGCTAGCATGGATGTTTTCCCAGTCACGACGTTGTAAAACGACGGCCAGTCTTAAGC
TCGGGCCCCAAATAATGATTTTATTTTGACTGATAGTGACCTGTTCGTTGCAACAAATTGATGAGCAATG
CTTTTTTATAATGCCAACTTTGTACAAAAAAGCAGGCTCCGCGGCCGCCCCCTTCACCAAACCTAAACCT
AAACGGCTAAGCCCGACGTCAAATACCAAAAAGAGAAAAACAAGAGCGCCGTCAAGCTCTGCAAATACGA
TCTGTAAGTCCATCTTAACACAAAAGTGAGATGGGTTCTTAGATCATGTTCCGCCGTTAGATCGAGTCAT
GGTCTTGTCTCATAGAAAGGTACTTTCGTTTACTTCTTTTGAGTATCGAGTAGAGCGTCGTCTATAGTTA
GTTTGAGATTGCGTTTGTCAGAAGTTAGGTTCAATGTCCCGGTCCAATTTTCACCAGCCATGTGTCAGTT
TCGTTCCTTCCCGTCCTCTTCTTTGATTTCGTTGGGTTACGGATGTTTTCGAGATGAAACAGCATTGTTT
TGTTGTGATTTTTCTCTACAAGCGAATAGACCATTTAAGAGACCATTAGGCACCCCAGGCTTTACACTTT
ATGCTTCCGGCTCGTATAATGTGTGGATTTTGAGTTAGGAGCCGTCGAGATTTTCAGGAGCTAAGGAAGC
TAAAatggagaaaaaaatcactggatataccaccgttgatatatcccaatggcatcgtaaagaacatttt
gaggcatttcagtcagttgctcaatgtacctataaccagaccgttcagctggatattacggcctttttaa
agaccgtaaagaaaaataagcacaagttttatccggcctttattcacattcttgcccgcctgatgaatgc
tcatccggagttccgtatggcaatgaaagacggtgagctggtgatatgggatagtgttcacccttgttac
accgttttccatgagcaaactgaaacgttttcatcgctctggagtgaataccacgacgatttccggcagt
ttctacacatatattcgcaagatgtggcgtgttacggtgaaaacctggcctatttccctaaagggtttat
tgagaatatgtttttcgtctcagccaatccctgggtgagtttcaccagttttgatttaaacgtggccaat
atggacaacttcttcgcccccgttttcaccatgggcaaatattatacgcaaggcgacaaggtgctgatgc
cgctggcgattcaggttcatcatgccgtttgtgatggcttccatgtcggcagaatgcttaatgaattaca
acagtactgcgatgagtggcagggcggggcgtaaACGCGTGGAGCCGGCTTACTAAAAGCCAGATAACAG
TATGCGTATTTGCGCGCTGATTTTTGCGGTATAAGAATATATACTGATATGTATACCCGAAGTATGTCAA

AAAGAGGTATGCTATGAAGCAGCGTATTACAGTGACAGTTGACAGCGACAGCTATCAGTTGCTCAAGGCA
TATATGATGTCAATATCTCCGGTCTGGTAAGCACAACCATGCAGAATGAAGCCCGTCGTCTGCGTGCCGA
ACGCTGGAAAGCGGAAAATCAGGAAGGGATGGCTGAGGTCGCCCGGTTTATTGAAATGAACGGCTCTTTT
GCTGACGAGAACAGGGGCTGGTGAAATGCAGTTTAAGGTTTACACCTATAAAAGAGAGAGCCGTTATCGT
CTGTTTGTGGATGTACAGAGTGATATTATTGACACGCCCGGCCGACGGATGGTGATCCCCCTGGCCAGTG
CACGTCTGCTGTCAGATAAAGTCTCCCGTGAACTTTACCCGGTGGTGCATATCGGGGATGAAAGCTGGCG
CATGATGACCACCGATATGGCCAGTGTGCCGGTTTCCGTTATCGGGGAAGAAGTGGCTGATCTCAGCCAC
CGCGAAAATGACATCAAAAACGCCATTAACCTGATGTTCTGGGGAATATAAATGTCAGGCTCCCTTATAC
ACAGCCAGTCTGCACCTCGACggtctcAtcggtggatcttagaaaattattctaagtccaacatagcgta
ttctaagttcaacatatcgacgaactagaaaagacattggacatattccaggatatgcaaaagaaaacaa
tgaatattgttttgaatgtgttcaagtaaatgagattttcaagtcgtctaaagaacagttgctaatacag
ttacttatttcaataaataattggttctaataatacaaaacatattcgaggatatgcagaaaaaaagatg
tttgttattttgaaaagcttgagtagtttctctccgaggtgtagcgaagaagcatcatctactttgtaat
gtaattttctttatgttttcactttgtaattttatttgtgttaatgtaccatggccgatatcggttttat
tgaaagaaaatttatgttacttctgttttggctttgcaatcagttatgctagttttcttataccctttcg
taagcttcctaaggaatcgttcattgatttccactgcttcattgtatattaaaactttacaactgtatcg
accatcatataattctgggtcaagagatgaaaatagaacaccacatcgtaaagtgaaatAAGGGTGGGCG
CGCCGACCCAGCTTTCTTGTACAAAGTTGGCATTATAAGAAAGCATTGCTTATCAATTTGTTGCAACGAA
CAGGTCACTATCAGTCAAAATAAAATCATTATTTGCCATCCAGCTGATATCCCCTATAGTGAGTCGTATT
ACATGGTCATAGCTGTTTCCTGGCAGCTCTGGCCCGTGTCTCAAAATCTCTGATGTTACATTGCACAAGA
TAAAAATATATCATCATGAACAATAAAACTGTCTGCTTACATAAACAGTAATACAAGGGGTGTTatgagc
catattcaacgggaaacgtcgaggccgcgattaaattccaacatggatgctgatttatatgggtataaat
gggctcgcgataatgtcgggcaatcaggtgcgacaatctatcgcttgtatgggaagcccgatgcgccaga
gttgtttctgaaacatggcaaaggtagcgttgccaatgatgttacagatgagatggtcagactaaactgg
ctgacggaatttatgcctcttccgaccatcaagcattttatccgtactcctgatgatgcatggttactca
ccactgcgatccccggaaaaacagcattccaggtattagaagaatatcctgattcaggtgaaaatattgt
tgatgcgctggcagtgttcctgcgccggttgcattcgattcctgtttgtaattgtccttttaacagcgat
cgcgtatttcgtctcgctcaggcgcaatcacgaatgaataacggtttggttgatgcgagtgattttgatg
acgagcgtaatggctggcctgttgaacaagtctggaaagaaatgcataaacttttgccattctcaccgga
ttcagtcgtcactcatggtgatttctcacttgataaccttatttttgacgaggggaaattaataggttgt
attgatgttggacgagtcggaatcgcagaccgataccaggatcttgccatcctatggaactgcctcggtg
agttttctccttcattacagaaacggctttttcaaaaatatggtattgataatcctgatatgaataaatt
gcagtttcatttgatgctcgatgagtttttcTAATCAGAATTGGTTAATTGGTTGTAACACTGGCAGAGC

ATTACGCTGACTTGACGGGACGGCGCAAGCTCATGACCAAAATCCCTTAACGTGAGTTACGCGTCGTTCC

ACTGAGCGTCAGACCCCGTAGAAAAGATCAAAGGATCTTCTTGAGATCCTTTTTTTCTGCGCGTAATCTG

CTGCTTGCAAACAAAAAAACCACCGCTACCAGCGGTGGTTTGTTTGCCGGATCAAGAGCTACCAACTCTT

TTTCCGAAGGTAACTGGCTTCAGCAGAGCGCAGATACCAAATACTGTCCTTCTAGTGTAGCCGTAGTTAG

GCCACCACTTCAAGAACTCTGTAGCACCGCCTACATACCTCGCTCTGCTAATCCTGTTACCAGTGGCTGC

TGCCAGTGGCGATAAGTCGTGTCTTACCGGGTTGGACTCAAGACGATAGTTACCGGATAAGGCGCAGCGG

TCGGGCTGAACGGGGGGTTCGTGCACACAGCCCAGCTTGGAGCGAACGACCTACACCGAACTGAGATACC

TACAGCGTGAGCATTGAGAAAGCGCCACGCTTCCCGAAGGGAGAAAGGCGGACAGGTATCCGGTAAGCGG

CAGGGTCGGAACAGGAGAGCGCACGAGGGAGCTTCCAGGGGGAAACGCCTGGTATCTTTATAGTCCTGTC

GGGTTTCGCCACCTCTGACTTGAGCGTCGATTTTTGTGATGCTCGTCAGGGGGGCGGAGCCTATGGAAAA

ACGCCAGCAACGCGGCCTTTTTACGGTTCCTGGCCTTTTGCTGGCCTTTTGCTCACATGTT

PURPLE/UPPERCASE: M13-F binding site

orange/lowercase: attL1

BLUE/UPPERCASE: *AtTAS1c 5'* region

RED/UPPERCASE: *Bsa*I site

red/lowercase: inverted *Bsa*I site

magenta/lowercase: Chloramphenicol resistance gene

MAGENTA/UPPERCASE: *ccd*B gene

blue/lowercase: *AtTAS1c* 3' region

orange/lowercase/underlined: attL2

PURPLE/UPPERCASE/UNDERLINED: M13-R binding site

brown/lowercase: Kanamycin resistance gene

>*pMDC32B-AtTAS1c-D2-B/c* (12592 bp)

CCAGCCAGCCAACAGCTCCCCGACCGGCAGCTCGGCACAAAATCACCACTCGATACAGGCAGCCCATCAG
TCCGGGACGGCGTCAGCGGGAGAGCCGTTGTAAGGCGGCAGACTTTGCTCATGTTACCGATGCTATTCGG
AAGAACGGCAACTAAGCTGCCGGGTTTGAAACACGGATGATCTCGCGGAGGGTAGCATGTTGATTGTAAC
GATGACAGAGCGTTGCTGCCTGTGATCACCGCGGTTTCAAAATCGGCTCCGTCGATACTATGTTATACGC
CAACTTTGAAAACAACTTTGAAAAAGCTGTTTTCTGGTATTTAAGGTTTTAGAATGCAAGGAACAGTGAA
TTGGAGTTCGTCTTGTTATAATTAGCTTCTTGGGGTATCTTTAAATACTGTAGAAAAGAGGAAGGAAATA
ATAAatggctaaaatgagaatatcaccggaattgaaaaaactgatcgaaaaataccgctgcgtaaaagat
acggaaggaatgtctcctgctaaggtatataagctggtgggagaaaatgaaaacctatatttaaaaatga
cggacagccggtataaagggaccacctatgatgtggaacgggaaaaggacatgatgctatggctggaagg
aaagctgcctgttccaaaggtcctgcactttgaacggcatgatggctggagcaatctgctcatgagtgag
gccgatggcgtcctttgctcggaagagtatgaagatgaacaaagccctgaaaagattatcgagctgtatg
cggagtgcatcaggctctttcactccatcgacatatcggattgtccctatacgaatagcttagacagccg
cttagccgaattggattacttactgaataacgatctggccgatgtggattgcgaaaactgggaagaagac
actccatttaaagatccgcgcgagctgtatgatttttttaaagacggaaaagcccgaagaggaacttgtct
tttcccacggcgacctgggagacagcaacatctttgtgaaagatggcaaagtaagtggctttattgatct
tgggagaagcggcagggcggacaagtggtatgacattgccttctgcgtccggtcgatcagggaggatatc
ggggaagaacagtatgtcgagctatttttttgacttactggggatcaagcctgattgggagaaaataaaat
attatattttactggatgaattgttttagTACCTAGAATGCATGACCAAAATCCCTTAACGTGAGTTTTC
GTTCCACTGAGCGTCAGACCCCGTAGAAAAGATCAAAGGATCTTCTTGAGATCCTTTTTTTCTGCGCGTA
ATCTGCTGCTTGCAAACAAAAAAACCACCGCTACCAGCGGTGGTTTGTTTGCCGGATCAAGAGCTACCAA
CTCTTTTTCCGAAGGTAACTGGCTTCAGCAGAGCGCAGATACCAAATACTGTCCTTCTAGTGTAGCCGTA
GTTAGGCCACCACTTCAAGAACTCTGTAGCACCGCCTACATACCTCGCTCTGCTAATCCTGTTACCAGTG
GCTGCTGCCAGTGGCGATAAGTCGTGTCTTACCGGGTTGGACTCAAGACGATAGTTACCGGATAAGGCGC
AGCGGTCGGGCTGAACGGGGGGTTCGTGCACACAGCCCAGCTTGGAGCGAACGACCTACACCGAACTGAG
ATACCTACAGCGTGAGCTATGAGAAAGCGCCACGCTTCCCGAAGGGAGAAAGGCGGACAGGTATCCGGTA
AGCGGCAGGGTCGGAACAGGAGAGCGCACGAGGGAGCTTCCAGGGGGAAACGCCTGGTATCTTTATAGTC
CTGTCGGGTTTCGCCACCTCTGACTTGAGCGTCGATTTTTGTGATGCTCGTCAGGGGGGCGGAGCCTATG
GAAAAACGCCAGCAACGCGGCCTTTTTACGGTTCCTGGCCTTTTGCTGGCCTTTTGCTCACATGTTCTTT
CCTGCGTTATCCCCTGATTCTGTGGATAACCGTATTACCGCCTTTGAGTGAGCTGATACCGCTCGCCGCA
GCCGAACGACCGAGCGCAGCGAGTCAGTGAGCGAGGAAGCGGAAGAGCGCCTGATGCGGTATTTTCTCCT
TACGCATCTGTGCGGTATTTCACACCGCATATGGTGCACTCTCAGTACAATCTGCTCTGATGCCGCATAG
TTAAGCCAGTATACACTCCGCTATCGCTACGTGACTGGGTCATGGCTGCGCCCCGACACCCGCCAACACC

CGCTGACGCGCCCTGACGGGCTTGTCTGCTCCCGGCATCCGCTTACAGACAAGCTGTGACCGTCTCCGGG
AGCTGCATGTGTCAGAGGTTTTCACCGTCATCACCGAAACGCGCGAGGCAGGGTGCCTTGATGTGGGCGC
CGGCGGTCGAGTGGCGACGGCGCGGCTTGTCCGCGCCCTGGTAGATTGCCTGGCCGTAGGCCAGCCATTT
TTGAGCGGCCAGCGGCCGCGATAGGCCGACGCGAAGCGGCGGGGCGTAGGGAGCGCAGCGACCGAAGGGT
AGGCGCTTTTTGCAGCTCTTCGGCTGTGCGCTGGCCAGACAGTTATGCACAGGCCAGGCGGGTTTTAAGA
GTTTTAATAAGTTTTAAAGAGTTTTAGGCGGAAAAATCGCCTTTTTTCTCTTTTATATCAGTCACTTACA
TGTGTGACCGGTTCCCAATGTACGGCTTTGGGTTCCCAATGTACGGGTTCCGGTTCCCAATGTACGGCTT
TGGGTTCCCAATGTACGTGCTATCCACAGGAAAGAGAACTTTTCGACCTTTTTCCCCTGCTAGGGCAATT
TGCCCTAGCATCTGCTCCGTACATTAGGAACCGGCGGATGCTTCGCCCTCGATCAGGTTGCGGTAGCGCA
TGACTAGGATCGGGCCAGCCTGCCCCGCCTCCTCCTTCAAATCGTACTCCGGCAGGTCATTTGACCCGAT
CAGCTTGCGCACGGTGAAACAGAACTTCTTGAACTCTCCGGCGCTGCCACTGCGTTCGTAGATCGTCTTG
AACAACCATCTGGCTTCTGCCTTGCCTGCGGCGCGGCGTGCCAGGCGGTAGAGAAAACGGCCGATGCCGG
GATCGATCAAAAAGTAATCGGGGTGAACCGTCAGCACGTCCGGGTTCTTGCCTTCTGTGATCTCGCGGTA
CATCCAATCAGCTAGCTCGATCTCGATGTACTCCGGCCGCCCGGTTTCGCTCTTTACGATCTTGTAGCGG
CTAATCAAGGCTTCACCCTCGGATACCGTCACCAGGCGGCCGTTCTTGGCCTTCTTCGTACGCTGCATGG
CAACGTGCGTGGTGTTTAACCGAATGCAGGTTTCTACCAGGTCGTCTTTCTGCTTTCCGCCATCGGCTCG
CCGGCAGAACTTGAGTACGTCCGCAACGTGTGGACGGAACACGCGGCCGGGCTTGTCTCCCTTCCCTTCC
CGGTATCGGTTCATGGATTCGGTTAGATGGGAAACCGCCATCAGTACCAGGTCGTAATCCCACACACTGG
CCATGCCGGCCGGCCCTGCGGAAACCTCTACGTGCCCGTCTGGAAGCTCGTAGCGGATCACCTCGCCAGC
TCGTCGGTCACGCTTCGACAGACGGAAAACGGCCACGTCCATGATGCTGCGACTATCGCGGGTGCCCACG
TCATAGAGCATCGGAACGAAAAAATCTGGTTGCTCGTCGCCCTTGGGCGGCTTCCTAATCGACGGCGCAC
CGGCTGCCGGCGGTTGCCGGGATTCTTTGCGGATTCGATCAGCGGCCGCTTGCCACGATTCACCGGGGCG
TGCTTCTGCCTCGATGCGTTGCCGCTGGGCGGCCTGCGCGGCCTTCAACTTCTCCACCAGGTCATCACCC
AGCGCCGCGCCGATTTGTACCGGGCCGGATGGTTTGCGACCGTCACGCCGATTCCTCGGGCTTGGGGGTT
CCAGTGCCATTGCAGGGCCGGCAGACAACCCAGCCGCTTACGCCTGGCCAACCGCCCGTTCCTCCACACA
TGGGGCATTCCACGGCGTCGGTGCCTGGTTGTTCTTGATTTTCCATGCCGCCTCCTTTAGCCGCTAAAAT
TCATCTACTCATTTATTCATTTGCTCATTTACTCTGGTAGCTGCGCGATGTATTCAGATAGCAGCTCGGT
AATGGTCTTGCCTTGGCGTACCGCGTACATCTTCAGCTTGGTGTGATCCTCCGCCGGCAACTGAAAGTTG
ACCCGCTTCATGGCTGGCGTGTCTGCCAGGCTGGCCAACGTTGCAGCCTTGCTGCTGCGTGCGCTCGGAC
GGCCGGCACTTAGCGTGTTTGTGCTTTTGCTCATTTTCTCTTTACCTCATTAACTCAAATGAGTTTTGAT
TTAATTTCAGCGGCCAGCGCCTGGACCTCGCGGGCAGCGTCGCCCTCGGGTTCTGATTCAAGAACGGTTG
TGCCGGCGGCGGCAGTGCCTGGGTAGCTCACGCGCTGCGTGATACGGGACTCAAGAATGGGCAGCTCGTA
CCCGGCCAGCGCCTCGGCAACCTCACCGCCGATGCGCGTGCCTTTGATCGCCCGCGACACGACAAAGGCC

GCTTGTAGCCTTCCATCCGTGACCTCAATGCGCTGCTTAACCAGCTCCACCAGGTCGGCGGTGGCCCATA
TGTCGTAAGGGCTTGGCTGCACCGGAATCAGCACGAAGTCGGCTGCCTTGATCGCGGACACAGCCAAGTC
CGCCGCCTGGGGCGCTCCGTCGATCACTACGAAGTCGCGCCGGCCGATGGCCTTCACGTCGCGGTCAATC
GTCGGGCGGTCGATGCCGACAACGGTTAGCGGTTGATCTTCCCGCACGGCCGCCCAATCGCGGGCACTGC
CCTGGGGATCGGAATCGACTAACAGAACATCGGCCCCGGCGAGTTGCAGGGCGCGGGCTAGATGGGTTGC
GATGGTCGTCTTGCCTGACCCGCCTTTCTGGTTAAGTACAGCGATAACCTTCATGCGTTCCCCTTGCGTA
TTTGTTTATTTACTCATCGCATCATATACGCAGCGACCGCATGACGCAAGCTGTTTTACTCAAATACACA
TCACCTTTTTAGACGGCGGCGCTCGGTTTCTTCAGCGGCCAAGCTGGCCGGCCAGGCCGCCAGCTTGGCA
TCAGACAAACCGGCCAGGATTTCATGCAGCCGCACGGTTGAGACGTGCGCGGGCGGCTCGAACACGTACC
CGGCCGCGATCATCTCCGCCTCGATCTCTTCGGTAATGAAAAACGGTTCGTCCTGGCCGTCCTGGTGCGG
TTTCATGCTTGTTCCTCTTGGCGTTCATTCTCGGCGGCCGCCAGGGCGTCGGCCTCGGTCAATGCGTCCT
CACGGAAGGCACCGCGCCGCCTGGCCTCGGTGGGCGTCACTTCCTCGCTGCGCTCAAGTGCGCGGTACAG
GGTCGAGCGATGCACGCCAAGCAGTGCAGCCGCCTCTTTCACGGTGCGGCCTTCCTGGTCGATCAGCTCG
CGGGCGTGCGCGATCTGTGCCGGGGTGAGGGTAGGGCGGGGGCCAAACTTCACGCCTCGGGCCTTGGCGG
CCTCGCGCCCGCTCCGGGTGCGGTCGATGATTAGGGAACGCTCGAACTCGGCAATGCCGGCGAACACGGT
CAACACCATGCGGCCGGCCGGCGTGGTGGTGTCGGCCCACGGCTCTGCCAGGCTACGCAGGCCCGCGCCG
GCCTCCTGGATGCGCTCGGCAATGTCCAGTAGGTCGCGGGTGCTGCGGGCCAGGCGGTCTAGCCTGGTCA
CTGTCACAACGTCGCCAGGGCGTAGGTGGTCAAGCATCCTGGCCAGCTCCGGGCGGTCGCGCCTGGTGCC
GGTGATCTTCTCGGAAAACAGCTTGGTGCAGCCGGCCGCGTGCAGTTCGGCCCGTTGGTTGGTCAAGTCC
TGGTCGTCGGTGCTGACGCGGGCATAGCCCAGCAGGCCAGCGGCGGCGCTCTTGTTCATGGCGTAATGTC
TCCGGTTCTAGTCGCAAGTATTCTACTTTATGCGACTAAAACACGCGACAAGAAAACGCCAGGAAAAGGG
CAGGGCGGCAGCCTGTCGCGTAACTTAGGACTTGTGCGACATGTCGTTTTCAGAAGACGGCTGCACTGAA
CGTCAGAAGCCGACTGCACTATAGCAGCGGAGGGGTTGGATCAAAGTACTTTGATCCCGAGGGGAACCCT
GTGGTTGGCATGCACATACAAATGGACGAACGGATAAACCTTTTCACGCCCTTTTAAATATCCGTTATTC
TAATAAACGCTCTTTTCTCTTAGGtttacccgccaatatatcctgtcaAACACTGATAGTTTAAACTGAA
GGCGGGAAACGACAATCTGATCCAAGCTCAAGCTGCTCTAGCATTCGCCATTCAGGCTGCGCAACTGTTG
GGAAGGGCGATCGGTGCGGGCCTCTTCGCTATTACGCCAGCTGGCGAAAGGGGGATGTGCTGCAAGGCGA
TTAAGTTGGGTAACGCCAGGGTTTTCCCAGTCACGACGTTGTAAAACGACGGCCAGTGCCAAGCTTGGCG
TGCCTGCAGGTCAACATGGTGGAGCACGACACACTTGTCTACTCCAAAAATATCAAAGATACAGTCTCAG
AAGACCAAAGGGCAATTGAGACTTTTCAACAAAGGGTAATATCCGGAAACCTCCTCGGATTCCATTGCCC
AGCTATCTGTCACTTTATTGTGAAGATAGTGGAAAAGGAAGGTGGCTCCTACAAATGCCATCATTGCGAT
AAAGGAAAGGCCATCGTTGAAGATGCCTCTGCCGACAGTGGTCCCAAAGATGGACCCCCACCCACGAGGA
GCATCGTGGAAAAAGAAGACGTTCCAACCACGTCTTCAAAGCAAGTGGATTGATGTGATAACATGGTGGA

GCACGACACACTTGTCTACTCCAAAAATATCAAAGATACAGTCTCAGAAGACCAAAGGGCAATTGAGACT
TTTCAACAAAGGGTAATATCCGGAAACCTCCTCGGATTCCATTGCCCAGCTATCTGTCACTTTATTGTGA
AGATAGTGGAAAAGGAAGGTGGCTCCTACAAATGCCATCATTGCGATAAAGGAAAGGCCATCGTTGAAGA
TGCCTCTGCCGACAGTGGTCCCAAAGATGGACCCCCACCCACGAGGAGCATCGTGGAAAAAGAAGACGTT
CCAACCACGTCTTCAAAGCAAGTGGATTGATGTGATATCTCCACTGACGTAAGGGATGACGCACAATCCC
ACTATCCTTCGCAAGACCCTTCCTCTATATAAGGAAGTTCATTTCATTTGGAGAGGACCTCGACTCTAGA
GGATCCCCGGGTACCGGGCCCCCCCTCGAGGCGCGCCAAGCTATCAAACAAGTTTGTACAAAAAAGCAGG
CTCCGCGGCCGCCCCCTTCACCCCTTCACCAAACCTAAACCTAAACGGCTAAGCCCGACGTCAAATACCA
AAAAGAGAAAAACAAGAGCGCCGTCAAGCTCTGCAAATACGATCTGTAAGTCCATCTTAACACAAAAGTG
AGATGGGTTCTTAGATCATGTTCCGCCGTTAGATCGAGTCATGGTCTTGTCTCATAGAAAGGTACTTTCG
TTTACTTCTTTTGAGTATCGAGTAGAGCGTCGTCTATAGTTAGTTTGAGATTGCGTTTGTCAGAAGTTAG
GTTCAATGTCCCGGTCCAATTTTCACCAGCCATGTGTCAGTTTCGTTCCTTCCCGTCCTCTTCTTTGATT
TCGTTGGGTTACGGATGTTTTCGAGATGAAACAGCATTGTTTTGTTGTGATTTTTCTCTACAAGCGAATA
GACCATTTAAGAGACCATTAGGCACCCCAGGCTTTACACTTTATGCTTCCGGCTCGTATAATGTGTGGAT
TTTGAGTTAGGAGCCGTCGAGATTTTCAGGAGCTAAGGAAGCTAAAatggagaaaaaaatcactggatat
accaccgttgatatatcccaatggcatcgtaaagaacattttgaggcatttcagtcagttgctcaatgta
cctataaccagaccgttcagctggatattacggcctttttaaagaccgtaaagaaaaataagcacaagtt
ttatccggcctttattcacattcttgcccgcctgatgaatgctcatccggagttccgtatggcaatgaaa
gacggtgagctggtgatatgggatagtgttcacccttgttacaccgttttccatgagcaaactgaaacgt
tttcatcgctctggagtgaataccacgacgatttccggcagtttctacacatatattcgcaagatgtggc
gtgttacggtgaaaacctggcctatttccctaaagggtttattgagaatatgtttttcgtctcagccaat
ccctgggtgagtttcaccagttttgatttaaacgtggccaatatggacaacttcttcgcccccgttttca
ccatgggcaaatattatacgcaaggcgacaaggtgctgatgccgctggcgattcaggttcatcatgccgt
ttgtgatggcttccatgtcggcagaatgcttaatgaattacaacagtactgcgatgagtggcagggcggg
gcgtaaACGCGTGGAGCCGGCTTACTAAAAGCCAGATAACAGTATGCGTATTTGCGCGCTGATTTTTGCG
GTATAAGAATATATACTGATATGTATACCCGAAGTATGTCAAAAAGAGGTATGCTATGAAGCAGCGTATT
ACAGTGACAGTTGACAGCGACAGCTATCAGTTGCTCAAGGCATATATGATGTCAATATCTCCGGTCTGGT
AAGCACAACCATGCAGAATGAAGCCCGTCGTCTGCGTGCCGAACGCTGGAAAGCGGAAAATCAGGAAGGG
ATGGCTGAGGTCGCCCGGTTTATTGAAATGAACGGCTCTTTTGCTGACGAGAACAGGGGCTGGTGAAATG
CAGTTTAAGGTTTACACCTATAAAAGAGAGAGCCGTTATCGTCTGTTTGTGGATGTACAGAGTGATATTA
TTGACACGCCCGGCCGACGGATGGTGATCCCCCTGGCCAGTGCACGTCTGCTGTCAGATAAAGTCTCCCG
TGAACTTTACCCGGTGGTGCATATCGGGGATGAAAGCTGGCGCATGATGACCACCGATATGGCCAGTGTG
CCGGTTTCCGTTATCGGGGAAGAAGTGGCTGATCTCAGCCACCGCGAAAATGACATCAAAAACGCCATTA

ACCTGATGTTCTGGGGAATATAAATGTCAGGCTCCCTTATACACAGCCAGTCTGCACCTCGACggtctcA
tcggtggatcttagaaaattattctaagtccaacatagcgtattctaagttcaacatatcgacgaactag
aaaagacattggacatattccaggatatgcaaaagaaaacaatgaatattgttttgaatgtgttcaagta
aatgagattttcaagtcgtctaaagaacagttgctaatacagttacttatttcaataaataattggttct
aataatacaaaacatattcgaggatatgcagaaaaaaagatgtttgttattttgaaaagcttgagtagtt
tctctccgaggtgtagcgaagaagcatcatctactttgtaatgtaattttctttatgttttcactttgta
attttatttgtgttaatgtaccatggccgatatcggttttattgaaagaaaatttatgttacttctgttt
tggctttgcaatcagttatgctagttttcttataccctttcgtaagcttcctaaggaatcgttcattgat
ttccactgcttcattgtatattaaaactttacaactgtatcgaccatcatataattctgggtcaagagat
gaaaatagaacaccacatcgtaaagtgaaatAAGGGTGGGCGCGCCGACCCAGCTTTCTTGTACAAAGTG
GTTCGATAATTCCTTAATTAACTAGTTCTAGAGCGGCCGCCCACCGCGGTGGAGCTCGAATTTCCCCGAT
CGTTCAAACATTTGGCAATAAAGTTTCTTAAGATTGAATCCTGTTGCCGGTCTTGCGATGATTATCATAT
AATTTCTGTTGAATTACGTTAAGCATGTAATAATTAACATGTAATGCATGACGTTATTTATGAGATGGGT
TTTTATGATTAGAGTCCCGCAATTATACATTTAATACGCGATAGAAAACAAAATATAGCGCGCAAACTAG
GATAAATTATCGCGCGCGGTGTCATCTATGTTACTGAATTCGTAATCATGGTCATAGCTGTTTCCTGTGT
GAAATTGTTATCCGCTCACAATTCCACACAACATACGAGCCGGAAGCATAAAGTGTAAAGCCTGGGGTGC
CTAATGAGTGAGCTAACTCACATTAATTGCGTTGCGCTCACTGCCCGCTTTCCAGTCGGGAAACCTGTCG
TGCCAGCTGCATTAATGAATCGGCCAACGCGCGGGGAGAGGCGGTTTGCGTATTGGCTAGAGCAGCTTGC
CAACATGGTGGAGCACGACACTCTCGTCTACTCCAAGAATATCAAAGATACAGTCTCAGAAGACCAAAGG
GCTATTGAGACTTTTCAACAAAGGGTAATATCGGGAAACCTCCTCGGATTCCATTGCCCAGCTATCTGTC
ACTTCATCAAAAGGACAGTAGAAAAGGAAGGTGGCACCTACAAATGCCATCATTGCGATAAAGGAAAGGC
TATCGTTCAAGATGCCTCTGCCGACAGTGGTCCCAAAGATGGACCCCCACCCACGAGGAGCATCGTGGAA
AAAGAAGACGTTCCAACCACGTCTTCAAAGCAAGTGGATTGATGTGATAACatggtggagcacgacactc
tcgtctactccaagaatatcaaagatacagtctcagaagaccaaagggctattgagacttttcaacaaag
ggtaatatcgggaaacctcctcggattccattgcccagctatctgtcacttcatcaaaaggacagtagaa
aaggaaggtggcacctacaaatgccatcattgcgataaaggaaaggctatcgttcaagatgcctctgccg
acagtggtcccaaagatggacccccacccacgaggagcatcgtggaaaaagaagacgttccaaccacgtc
ttcaaagcaagtggattgatgtgatatctccactgacgtaagggatgacgcacaatcccactatccttcg
caagaccttcctctatataaggaagttcatttcatttggagaggACACGCTGAAATCACCAGTCTCTCTC
TACAAATCTATCTCTCTCGAGCTTTCGCAGATCCCGGGGGGCAATGAGATATGAAAAAGCCTGAACTCAC
CGCGACGTCTGTCGAGAAGTTTCTGATCGAAAAGTTCGACAGCGTCTCCGACCTGATGCAGCTCTCGGAG
GGCGAAGAATCTCGTGCTTTCAGCTTCGATGTAGGAGGGCGTGGATATGTCCTGCGGGTAAATAGCTGCG
CCGATGGTTTCTACAAAGATCGTTATGTTTATCGGCACTTTGCATCGGCCGCGCTCCCGATTCCGGAAGT

GCTTGACATTGGGGAGTTTAGCGAGAGCCTGACCTATTGCATCTCCCGCCGTGCACAGGGTGTCACGTTG
CAAGACCTGCCTGAAACCGAACTGCCCGCTGTTCTACAACCGGTCGCGGAGGCTATGGATGCGATCGCTG
CGGCCGATCTTAGCCAGACGAGCGGGTTCGGCCCATTCGGACCGCAAGGAATCGGTCAATACACTACATG
GCGTGATTTCATATGCGCGATTGCTGATCCCCATGTGTATCACTGGCAAACTGTGATGGACGACACCGTC
AGTGCGTCCGTCGCGCAGGCTCTCGATGAGCTGATGCTTTGGGCCGAGGACTGCCCCGAAGTCCGGCACC
TCGTGCACGCGGATTTCGGCTCCAACAATGTCCTGACGGACAATGGCCGCATAACAGCGGTCATTGACTG
GAGCGAGGCGATGTTCGGGGATTCCCAATACGAGGTCGCCAACATCTTCTTCTGGAGGCCGTGGTTGGCT
TGTATGGAGCAGCAGACGCGCTACTTCGAGCGGAGGCATCCGGAGCTTGCAGGATCGCCACGACTCCGGG
CGTATATGCTCCGCATTGGTCTTGACCAACTCTATCAGAGCTTGGTTGACGGCAATTTCGATGATGCAGC
TTGGGCGCAGGGTCGATGCGACGCAATCGTCCGATCCGGAGCCGGGACTGTCGGGCGTACACAAATCGCC
CGCAGAAGCGCGGCCGTCTGGACCGATGGCTGTGTAGAAGTACTCGCCGATAGTGGAAACCGACGCCCCA
GCACTCGTCCGAGGGCAAAGAAATAGAGTAGATGCCGACCGGATCTGTCGATCGACAAGCTCGAGtttct
ccataataatgtgtgagtagttcccagataagggaattagggttcctatagggtttcgctcatgtgttga
gcatataagaaacccttagtatgtatttgtatttgtaaaatacttctatcaataaaatttctaattccta
aaaccaaaatccagtactaaaatccagatcCCCCGAATTAATTCGGCGTTAATTCAGTACATTAAAAACG
TCCGCAATGTGTTATTAAGTTGTCTAAGCGTCAATTTGTTTACACCACAATATATCCTGCCA

brown/lowercase: kanamycin resistance gene

CYAN/UPPERCASE/UNDERLINED: C->A transversion to block vector's BsaI site

cyan/lowercase: T-DNA right border

GREEN/UPPERCASE: 2x35S CaMV promoter

ORANGE/UPPERCASE: attB1

BLUE/UPPERCASE: *AtTAS1c* 5' region

RED/UPPERCASE: *Bsa*I site

magenta/lowercase: chloramphenicol resistance gene

MAGENTA/UPPERCASE: *ccd*B gene

red/lowercase: inverted *Bsa*I site

blue/lowercase: *AtTAS1c* 3' region

ORANGE/UPPERCASE/UNDERLINED: attB2

GREY/UPPERCASE/UNDERLINED: Nos terminator

green/lowercase: CaMV promoter

BROWN/UPPERCASE: hygromycin resistance gene

green/lowercase/underlined: CaMV terminator

CYAN/UPPERCASE: T-DNA left border

>*pMDC32B-AtTAS1c-D2-B/c-AtMIR173* (14339 bp)

CCAGCCAGCCAACAGCTCCCCGACCGGCAGCTCGGCACAAAATCACCACTCGATACAGGCAGCCCATCAG
TCCGGGACGGCGTCAGCGGGAGAGCCGTTGTAAGGCGGCAGACTTTGCTCATGTTACCGATGCTATTCGG
AAGAACGGCAACTAAGCTGCCGGGTTTGAAACACGGATGATCTCGCGGAGGGTAGCATGTTGATTGTAAC
GATGACAGAGCGTTGCTGCCTGTGATCACCGCGGTTTCAAAATCGGCTCCGTCGATACTATGTTATACGC
CAACTTTGAAAACAACTTTGAAAAAGCTGTTTTCTGGTATTTAAGGTTTTAGAATGCAAGGAACAGTGAA
TTGGAGTTCGTCTTGTTATAATTAGCTTCTTGGGGTATCTTTAAATACTGTAGAAAAGAGGAAGGAAATA
ATAAatggctaaaatgagaatatcaccggaattgaaaaaactgatcgaaaaataccgctgcgtaaaagat
acggaaggaatgtctcctgctaaggtatataagctggtgggagaaaatgaaaacctatatttaaaaatga
cggacagccggtataaagggaccacctatgatgtggaacgggaaaaggacatgatgctatggctggaagg
aaagctgcctgttccaaaggtcctgcactttgaacggcatgatggctggagcaatctgctcatgagtgag
gccgatggcgtcctttgctcggaagagtatgaagatgaacaaagccctgaaaagattatcgagctgtatg
cggagtgcatcaggctctttcactccatcgacatatcggattgtccctatacgaatagcttagacagccg
cttagccgaattggattacttactgaataacgatctggccgatgtggattgcgaaaactgggaagaagac
actccatttaaagatccgcgcgagctgtatgatttttttaaagacggaaaagcccgaagaggaacttgtct
tttcccacggcgacctgggagacagcaacatctttgtgaaagatggcaaagtaagtggctttattgatct
tgggagaagcggcagggcggacaagtggtatgacattgccttctgcgtccggtcgatcagggaggatatc
ggggaagaacagtatgtcgagctatttttgacttactggggatcaagcctgattgggagaaaataaaat
attatattttactggatgaattgttttagTACCTAGAATGCATGACCAAAATCCCTTAACGTGAGTTTTC
GTTCCACTGAGCGTCAGACCCCGTAGAAAAGATCAAAGGATCTTCTTGAGATCCTTTTTTTCTGCGCGTA
ATCTGCTGCTTGCAAACAAAAAAACCACCGCTACCAGCGGTGGTTTGTTTGCCGGATCAAGAGCTACCAA
CTCTTTTTCCGAAGGTAACTGGCTTCAGCAGAGCGCAGATACCAAATACTGTCCTTCTAGTGTAGCCGTA
GTTAGGCCACCACTTCAAGAACTCTGTAGCACCGCCTACATACCTCGCTCTGCTAATCCTGTTACCAGTG
GCTGCTGCCAGTGGCGATAAGTCGTGTCTTACCGGGTTGGACTCAAGACGATAGTTACCGGATAAGGCGC
AGCGGTCGGGCTGAACGGGGGGTTCGTGCACACAGCCCAGCTTGGAGCGAACGACCTACACCGAACTGAG
ATACCTACAGCGTGAGCTATGAGAAAGCGCCACGCTTCCCGAAGGGAGAAAGGCGGACAGGTATCCGGTA
AGCGGCAGGGTCGGAACAGGAGAGCGCACGAGGGAGCTTCCAGGGGGAAACGCCTGGTATCTTTATAGTC
CTGTCGGGTTTCGCCACCTCTGACTTGAGCGTCGATTTTTGTGATGCTCGTCAGGGGGGCGGAGCCTATG
GAAAAACGCCAGCAACGCGGCCTTTTTACGGTTCCTGGCCTTTTGCTGGCCTTTTGCTCACATGTTCTTT
CCTGCGTTATCCCCTGATTCTGTGGATAACCGTATTACCGCCTTTGAGTGAGCTGATACCGCTCGCCGCA
GCCGAACGACCGAGCGCAGCGAGTCAGTGAGCGAGGAAGCGGAAGAGCGCCTGATGCGGTATTTTCTCCT
TACGCATCTGTGCGGTATTTCACACCGCATATGGTGCACTCTCAGTACAATCTGCTCTGATGCCGCATAG
TTAAGCCAGTATACACTCCGCTATCGCTACGTGACTGGGTCATGGCTGCGCCCCGACACCCGCCAACACC

CGCTGACGCGCCCTGACGGGCTTGTCTGCTCCCGGCATCCGCTTACAGACAAGCTGTGACCGTCTCCGGG

AGCTGCATGTGTCAGAGGTTTTCACCGTCATCACCGAAACGCGCGAGGCAGGGTGCCTTGATGTGGGCGC

CGGCGGTCGAGTGGCGACGGCGCGGCTTGTCCGCGCCCTGGTAGATTGCCTGGCCGTAGGCCAGCCATTT

TTGAGCGGCCAGCGGCCGCGATAGGCCGACGCGAAGCGGCGGGGCGTAGGGAGCGCAGCGACCGAAGGGT

AGGCGCTTTTTGCAGCTCTTCGGCTGTGCGCTGGCCAGACAGTTATGCACAGGCCAGGCGGGTTTTAAGA

GTTTTAATAAGTTTTAAAGAGTTTTAGGCGGAAAAATCGCCTTTTTTCTCTTTTATATCAGTCACTTACA

TGTGTGACCGGTTCCCAATGTACGGCTTTGGGTTCCCAATGTACGGGTTCCGGTTCCCAATGTACGGCTT

TGGGTTCCCAATGTACGTGCTATCCACAGGAAAGAGAACTTTTCGACCTTTTTCCCCTGCTAGGGCAATT

TGCCCTAGCATCTGCTCCGTACATTAGGAACCGGCGGATGCTTCGCCCTCGATCAGGTTGCGGTAGCGCA

TGACTAGGATCGGGCCAGCCTGCCCCGCCTCCTCCTTCAAATCGTACTCCGGCAGGTCATTTGACCCGAT

CAGCTTGCGCACGGTGAAACAGAACTTCTTGAACTCTCCGGCGCTGCCACTGCGTTCGTAGATCGTCTTG

AACAACCATCTGGCTTCTGCCTTGCCTGCGGCGCGGCGTGCCAGGCGGTAGAGAAAACGGCCGATGCCGG

GATCGATCAAAAAGTAATCGGGGTGAACCGTCAGCACGTCCGGGTTCTTGCCTTCTGTGATCTCGCGGTA

CATCCAATCAGCTAGCTCGATCTCGATGTACTCCGGCCGCCCGGTTTCGCTCTTTACGATCTTGTAGCGG

CTAATCAAGGCTTCACCCTCGGATACCGTCACCAGGCGGCCGTTCTTGGCCTTCTTCGTACGCTGCATGG

CAACGTGCGTGGTGTTTAACCGAATGCAGGTTTCTACCAGGTCGTCTTTCTGCTTTCCGCCATCGGCTCG

CCGGCAGAACTTGAGTACGTCCGCAACGTGTGGACGGAACACGCGGCCGGGCTTGTCTCCCTTCCCTTCC

CGGTATCGGTTCATGGATTCGGTTAGATGGGAAACCGCCATCAGTACCAGGTCGTAATCCCACACACTGG

CCATGCCGGCCGGCCCTGCGGAAACCTCTACGTGCCCGTCTGGAAGCTCGTAGCGGATCACCTCGCCAGC

TCGTCGGTCACGCTTCGACAGACGGAAAACGGCCACGTCCATGATGCTGCGACTATCGCGGGTGCCCACG

TCATAGAGCATCGGAACGAAAAAATCTGGTTGCTCGTCGCCCTTGGGCGGCTTCCTAATCGACGGCGCAC

CGGCTGCCGGCGGTTGCCGGGATTCTTTGCGGATTCGATCAGCGGCCGCTTGCCACGATTCACCGGGGCG

TGCTTCTGCCTCGATGCGTTGCCGCTGGGCGGCCTGCGCGGCCTTCAACTTCTCCACCAGGTCATCACCC

AGCGCCGCGCCGATTTGTACCGGGCCGGATGGTTTGCGACCGTCACGCCGATTCCTCGGGCTTGGGGGTT

CCAGTGCCATTGCAGGGCCGGCAGACAACCCAGCCGCTTACGCCTGGCCAACCGCCCGTTCCTCCACACA

TGGGGCATTCCACGGCGTCGGTGCCTGGTTGTTCTTGATTTTCCATGCCGCCTCCTTTAGCCGCTAAAAT

TCATCTACTCATTTATTCATTTGCTCATTTACTCTGGTAGCTGCGCGATGTATTCAGATAGCAGCTCGGT

AATGGTCTTGCCTTGGCGTACCGCGTACATCTTCAGCTTGGTGTGATCCTCCGCCGGCAACTGAAAGTTG

ACCCGCTTCATGGCTGGCGTGTCTGCCAGGCTGGCCAACGTTGCAGCCTTGCTGCTGCGTGCGCTCGGAC

GGCCGGCACTTAGCGTGTTTGTGCTTTTGCTCATTTTCTCTTTACCTCATTAACTCAAATGAGTTTTGAT

TTAATTTCAGCGGCCAGCGCCTGGACCTCGCGGGCAGCGTCGCCCTCGGGTTCTGATTCAAGAACGGTTG

TGCCGGCGGCGGCAGTGCCTGGGTAGCTCACGCGCTGCGTGATACGGGACTCAAGAATGGGCAGCTCGTA

CCCGGCCAGCGCCTCGGCAACCTCACCGCCGATGCGCGTGCCTTTGATCGCCCGCGACACGACAAAGGCC

GCTTGTAGCCTTCCATCCGTGACCTCAATGCGCTGCTTAACCAGCTCCACCAGGTCGGCGGTGGCCCATA

TGTCGTAAGGGCTTGGCTGCACCGGAATCAGCACGAAGTCGGCTGCCTTGATCGCGGACACAGCCAAGTC

CGCCGCCTGGGGCGCTCCGTCGATCACTACGAAGTCGCGCCGGCCGATGGCCTTCACGTCGCGGTCAATC

GTCGGGCGGTCGATGCCGACAACGGTTAGCGGTTGATCTTCCCGCACGGCCGCCCAATCGCGGGCACTGC

CCTGGGGATCGGAATCGACTAACAGAACATCGGCCCCGGCGAGTTGCAGGGCGCGGGCTAGATGGGTTGC

GATGGTCGTCTTGCCTGACCCGCCTTTCTGGTTAAGTACAGCGATAACCTTCATGCGTTCCCCTTGCGTA

TTTGTTTATTTACTCATCGCATCATATACGCAGCGACCGCATGACGCAAGCTGTTTTACTCAAATACACA

TCACCTTTTTAGACGGCGGCGCTCGGTTTCTTCAGCGGCCAAGCTGGCCGGCCAGGCCGCCAGCTTGGCA

TCAGACAAACCGGCCAGGATTTCATGCAGCCGCACGGTTGAGACGTGCGCGGGCGGCTCGAACACGTACC

CGGCCGCGATCATCTCCGCCTCGATCTCTTCGGTAATGAAAAACGGTTCGTCCTGGCCGTCCTGGTGCGG

TTTCATGCTTGTTCCTCTTGGCGTTCATTCTCGGCGGCCGCCAGGGCGTCGGCCTCGGTCAATGCGTCCT

CACGGAAGGCACCGCGCCGCCTGGCCTCGGTGGGCGTCACTTCCTCGCTGCGCTCAAGTGCGCGGTACAG

GGTCGAGCGATGCACGCCAAGCAGTGCAGCCGCCTCTTTCACGGTGCGGCCTTCCTGGTCGATCAGCTCG

CGGGCGTGCGCGATCTGTGCCGGGGTGAGGGTAGGGCGGGGGCCAAACTTCACGCCTCGGGCCTTGGCGG

CCTCGCGCCCGCTCCGGGTGCGGTCGATGATTAGGGAACGCTCGAACTCGGCAATGCCGGCGAACACGGT

CAACACCATGCGGCCGGCCGGCGTGGTGGTGTCGGCCCACGGCTCTGCCAGGCTACGCAGGCCCGCGCCG

GCCTCCTGGATGCGCTCGGCAATGTCCAGTAGGTCGCGGGTGCTGCGGGCCAGGCGGTCTAGCCTGGTCA

CTGTCACAACGTCGCCAGGGCGTAGGTGGTCAAGCATCCTGGCCAGCTCCGGGCGGTCGCGCCTGGTGCC

GGTGATCTTCTCGGAAAACAGCTTGGTGCAGCCGGCCGCGTGCAGTTCGGCCCGTTGGTTGGTCAAGTCC

TGGTCGTCGGTGCTGACGCGGGCATAGCCCAGCAGGCCAGCGGCGGCGCTCTTGTTCATGGCGTAATGTC

TCCGGTTCTAGTCGCAAGTATTCTACTTTATGCGACTAAAACACGCGACAAGAAAACGCCAGGAAAAGGG

CAGGGCGGCAGCCTGTCGCGTAACTTAGGACTTGTGCGACATGTCGTTTTCAGAAGACGGCTGCACTGAA

CGTCAGAAGCCGACTGCACTATAGCAGCGGAGGGGTTGGATCAAAGTACTTTGATCCCGAGGGGAACCCT

GTGGTTGGCATGCACATACAAATGGACGAACGGATAAACCTTTTCACGCCCTTTTAAATATCCGTTATTC

TAATAAACGCTCTTTTCTCTTAGGtttacccgccaatatatcctgtcaAACACTGATAGTTTAAACTGAA

GGCGGGAAACGACAATCTGATCCAAGCTCAAGCTGCTCTAGCATTCGCCATTCAGGCTGCGCAACTGTTG

GGAAGGGCGATCGGTGCGGGCCTCTTCGCTATTACGCCAGCTGGCGAAAGGGGGATGTGCTGCAAGGCGA

TTAAGTTGGGTAACGCCAGGGTTTTCCCAGTCACGACGTTGTAAAACGACGGCCAGTGCCAAGCTTGGCG

TGCCTGCAGGTCAACATGGTGGAGCACGACACACTTGTCTACTCCAAAAATATCAAAGATACAGTCTCAG

AAGACCAAAGGGCAATTGAGACTTTTCAACAAAGGGTAATATCCGGAAACCTCCTCGGATTCCATTGCCC

AGCTATCTGTCACTTTATTGTGAAGATAGTGGAAAAGGAAGGTGGCTCCTACAAATGCCATCATTGCGAT

AAAGGAAAGGCCATCGTTGAAGATGCCTCTGCCGACAGTGGTCCCAAAGATGGACCCCCACCCACGAGGA

GCATCGTGGAAAAAGAAGACGTTCCAACCACGTCTTCAAAGCAAGTGGATTGATGTGATAACATGGTGGA

GCACGACACACTTGTCTACTCCAAAAATATCAAAGATACAGTCTCAGAAGACCAAAGGGCAATTGAGACT
TTTCAACAAAGGGTAATATCCGGAAACCTCCTCGGATTCCATTGCCCAGCTATCTGTCACTTTATTGTGA
AGATAGTGGAAAAGGAAGGTGGCTCCTACAAATGCCATCATTGCGATAAAGGAAAGGCCATCGTTGAAGA
TGCCTCTGCCGACAGTGGTCCCAAAGATGGACCCCCACCCACGAGGAGCATCGTGGAAAAAGAAGACGTT
CCAACCACGTCTTCAAAGCAAGTGGATTGATGTGATATCTCCACTGACGTAAGGGATGACGCACAATCCC
ACTATCCTTCGCAAGACCCTTCCTCTATATAAGGAAGTTCATTTCATTTGGAGAGGACCTCGACTCTAGA
GGATCCCCGGGTACCGGGCCCCCCCTCGAGGCGCGCCAAGCTATCAAACAAGTTTGTACAAAAAAGCAGG
CTCCGCGGCCGCCCCCTTCACCCCTTCACCAAACCTAAACCTAAACGGCTAAGCCCGACGTCAAATACCA
AAAAGAGAAAAACAAGAGCGCCGTCAAGCTCTGCAAATACGATCTGTAAGTCCATCTTAACACAAAAGTG
AGATGGGTTCTTAGATCATGTTCCGCCGTTAGATCGAGTCATGGTCTTGTCTCATAGAAAGGTACTTTCG
TTTACTTCTTTTGAGTATCGAGTAGAGCGTCGTCTATAGTTAGTTTGAGATTGCGTTTGTCAGAAGTTAG
GTTCAATGTCCCGGTCCAATTTTCACCAGCCATGTGTCAGTTTCGTTCCTTCCCGTCCTCTTCTTTGATT
TCGTTGGGTTACGGATGTTTTCGAGATGAAACAGCATTGTTTTGTTGTGATTTTTCTCTACAAGCGAATA
GACCATTTAAGAGACCATTAGGCACCCCAGGCTTTACACTTTATGCTTCCGGCTCGTATAATGTGTGGAT
TTTGAGTTAGGAGCCGTCGAGATTTTCAGGAGCTAAGGAAGCTAAAatggagaaaaaaatcactggatat
accaccgttgatatatcccaatggcatcgtaaagaacattttgaggcatttcagtcagttgctcaatgta
cctataaccagaccgttcagctggatattacggcctttttaaagaccgtaaagaaaaataagcacaagtt
ttatccggcctttattcacattcttgcccgcctgatgaatgctcatccggagttccgtatggcaatgaaa
gacggtgagctggtgatatgggatagtgttcacccttgttacaccgttttccatgagcaaactgaaacgt
tttcatcgctctggagtgaataccacgacgatttccggcagtttctacacatatattcgcaagatgtggc
gtgttacggtgaaaacctggcctatttccctaaagggtttattgagaatatgtttttcgtctcagccaat
ccctgggtgagtttcaccagttttgatttaaacgtggccaatatggacaacttcttcgcccccgttttca
ccatgggcaaatattatacgcaaggcgacaaggtgctgatgccgctggcgattcaggttcatcatgccgt
ttgtgatggcttccatgtcggcagaatgcttaatgaattacaacagtactgcgatgagtggcagggcggg
gcgtaaACGCGTGGAGCCGGCTTACTAAAAGCCAGATAACAGTATGCGTATTTGCGCGCTGATTTTTGCG
GTATAAGAATATATACTGATATGTATACCCGAAGTATGTCAAAAAGAGGTATGCTATGAAGCAGCGTATT
ACAGTGACAGTTGACAGCGACAGCTATCAGTTGCTCAAGGCATATATGATGTCAATATCTCCGGTCTGGT
AAGCACAACCATGCAGAATGAAGCCCGTCGTCTGCGTGCCGAACGCTGGAAAGCGGAAAATCAGGAAGGG
ATGGCTGAGGTCGCCCGGTTTATTGAAATGAACGGCTCTTTTGCTGACGAGAACAGGGGCTGGTGAAATG
CAGTTTAAGGTTTACACCTATAAAAGAGAGAGCCGTTATCGTCTGTTTGTGGATGTACAGAGTGATATTA
TTGACACGCCCGGCCGACGGATGGTGATCCCCCTGGCCAGTGCACGTCTGCTGTCAGATAAAGTCTCCCG
TGAACTTTACCCGGTGGTGCATATCGGGGATGAAAGCTGGCGCATGATGACCACCGATATGGCCAGTGTG
CCGGTTTCCGTTATCGGGGAAGAAGTGGCTGATCTCAGCCACCGCGAAAATGACATCAAAAACGCCATTA

ACCTGATGTTCTGGGGAATATAAATGTCAGGCTCCCTTATACACAGCCAGTCTGCACCTCGACggtctcA
tcggtggatcttagaaaattattctaagtccaacatagcgtattctaagttcaacatatcgacgaactag
aaaagacattggacatattccaggatatgcaaaagaaaacaatgaatattgttttgaatgtgttcaagta
aatgagattttcaagtcgtctaaagaacagttgctaatacagttacttatttcaataaataattggttct
aataatacaaaacatattcgaggatatgcagaaaaaaagatgtttgttattttgaaaagcttgagtagtt
tctctccgaggtgtagcgaagaagcatcatctactttgtaatgtaattttctttatgttttcactttgta
attttatttgtgttaatgtaccatggccgatatcggttttattgaaagaaaatttatgttacttctgttt
tggctttgcaatcagttatgctagttttcttataccctttcgtaagcttcctaaggaatcgttcattgat
ttccactgcttcattgtatattaaaactttacaactgtatcgaccatcatataattctgggtcaagagat
gaaaatagaacaccacatcgtaaagtgaaatAAGGGTGGGCGCGCCGACCCAGCTTTCTTGTACAAAGTG
GTTCGATAATTCCTTAATTAACTAGTTCTAGAGCGGCCGCCCACCGCGGTGGAGCTCGAATTTCCCCGAT
CGTTCAAACATTTGGCAATAAAGTTTCTTAAGATTGAATCCTGTTGCCGGTCTTGCGATGATTATCATAT
AATTTCTGTTGAATTACGTTAAGCATGTAATAATTAACATGTAATGCATGACGTTATTTATGAGATGGGT
TTTTATGATTAGAGTCCCGCAATTATACATTTAATACGCGATAGAAAACAAAATATAGCGCGCAAACTAG
GATAAATTATCGCGCGCGGTGTCATCTATGTTACTGAATTCaagcttggcgtgcctgcaGGTCAACATGG
TGGAGCACGACACACTTGTCTACTCCAAAAATATCAAAGATACAGTCTCAGAAGACCAAAGGGCAATTGA
GACTTTTCAACAAAGGGTAATATCCGGAAACCTCCTCGGATTCCATTGCCCAGCTATCTGTCACTTTATT
GTGAAGATAGTGGAAAAGGAAGGTGGCTCCTACAAATGCCATCATTGCGATAAAGGAAAGGCCATCGTTG
AAGATGCCTCTGCCGACAGTGGTCCCAAAGATGGACCCCCACCCACGAGGAGCATCGTGGAAAAAGAAGA
CGTTCCAACCACGTCTTCAAAGCAAGTGGATTGATGTGATAACATGGTGGAGCACGACACACTTGTCTAC
TCCAAAAATATCAAAGATACAGTCTCAGAAGACCAAAGGGCAATTGAGACTTTTCAACAAAGGGTAATAT
CCGGAAACCTCCTCGGATTCCATTGCCCAGCTATCTGTCACTTTATTGTGAAGATAGTGGAAAAGGAAGG
TGGCTCCTACAAATGCCATCATTGCGATAAAGGAAAGGCCATCGTTGAAGATGCCTCTGCCGACAGTGGT
CCCAAAGATGGACCCCCACCCACGAGGAGCATCGTGGAAAAAGAAGACGTTCCAACCACGTCTTCAAAGC
AAGTGGATTGATGTGATATCTCCACTGACGTAAGGGATGACGCACAATCCCACTATCCTTCGCAAGACCC
TTCCTCTATATAAGGAAGTTCATTTCATTTGGAGAGGacctcgactctagaggatccccgggtaccgggc
ccccctcgaggcgcgccaagctatcaaacaagtttgtacaaaaaagcaggctccgcggccgcccgcttc
accataattagcaagtaataaggaataaaatcctgtaattatagctgaaaaaggaaaaatatttggagtc
cgtcagattcgaatctgaacaaagcataaaaaagtcaacaaaacttaaagcggcggactcatcgtaatct
cagcccaataccctattttcctctcccctatataaatactttcttcttctactgatcttcttctcacaaa
taaacccaaatatatcaatctactgtgttggtgattaagtactttcgcttgcagagagaaatcacagtgg
tcaaaaaagttgtagttttcttaaagtctctttcctctgtgattctctgtgtaagcgaaagagcttgctc
cctaaacttatctctctgatgatttaatgttagagatcttcgtaaatctatgtgtttgatagatctgatg

cgtttttgagttgatgatttgattatttttcactggaaagtatctcattagggtaacgataatgtttta

tggatttggttgtataacagataagggtgggcgcgccgacccagctttcttgtacaaagtggttcgataa

ttccttaattaactagttctagagcggccgcccaccgcggtggagctcGAATTTCCCCGATCGTTCAAAC

ATTTGGCAATAAAGTTTCTTAAGATTGAATCCTGTTGCCGGTCTTGCGATGATTATCATATAATTTCTGT

TGAATTACGTTAAGCATGTAATAATTAACATGTAATGCATGACGTTATTTATGAGATGGGTTTTTATGAT

TAGAGTCCCGCAATTATACATTTAATACGCGATAGAAAACAAAATATAGCGCGCAAACTAGGATAAATTA

TCGCGCGCGGTGTCATCTATGTTactgaattcgaattcGTAATCATGGTCATAGCTGTTTCCTGTGTGAA

ATTGTTATCCGCTCACAATTCCACACAACATACGAGCCGGAAGCATAAAGTGTAAAGCCTGGGGTGCCTA

ATGAGTGAGCTAACTCACATTAATTGCGTTGCGCTCACTGCCCGCTTTCCAGTCGGGAAACCTGTCGTGC

CAGCTGCATTAATGAATCGGCCAACGCGCGGGGAGAGGCGGTTTGCGTATTGGCTAGAGCAGCTTGCCAA

CATGGTGGAGCACGACACTCTCGTCTACTCCAAGAATATCAAAGATACAGTCTCAGAAGACCAAAGGGCT

ATTGAGACTTTTCAACAAAGGGTAATATCGGGAAACCTCCTCGGATTCCATTGCCCAGCTATCTGTCACT

TCATCAAAAGGACAGTAGAAAAGGAAGGTGGCACCTACAAATGCCATCATTGCGATAAAGGAAAGGCTAT

CGTTCAAGATGCCTCTGCCGACAGTGGTCCCAAAGATGGACCCCCACCCACGAGGAGCATCGTGGAAAAA

GAAGACGTTCCAACCACGTCTTCAAAGCAAGTGGATTGATGTGATAACatggtggagcacgacactctcg

tctactccaagaatatcaaagatacagtctcagaagaccaaagggctattgagacttttcaacaaagggt

aatatcgggaaacctcctcggattccattgcccagctatctgtcacttcatcaaaaggacagtagaaaag

gaaggtggcacctacaaatgccatcattgcgataaaggaaaggctatcgttcaagatgcctctgccgaca

gtggtcccaaagatggacccccacccacgaggagcatcgtggaaaaagaagacgttccaaccacgtcttc

aaagcaagtggattgatgtgatatctccactgacgtaagggatgacgcacaatcccactatccttcgcaa

gaccttcctctatataaggaagttcatttcatttggagaggACACGCTGAAATCACCAGTCTCTCTCTAC

AAATCTATCTCTCTCGAGCTTTCGCAGATCCCGGGGGGCAATGAGATATGAAAAAGCCTGAACTCACCGC

GACGTCTGTCGAGAAGTTTCTGATCGAAAAGTTCGACAGCGTCTCCGACCTGATGCAGCTCTCGGAGGGC

GAAGAATCTCGTGCTTTCAGCTTCGATGTAGGAGGGCGTGGATATGTCCTGCGGGTAAATAGCTGCGCCG

ATGGTTTCTACAAAGATCGTTATGTTTATCGGCACTTTGCATCGGCCGCGCTCCCGATTCCGGAAGTGCT

TGACATTGGGGAGTTTAGCGAGAGCCTGACCTATTGCATCTCCCGCCGTGCACAGGGTGTCACGTTGCAA

GACCTGCCTGAAACCGAACTGCCCGCTGTTCTACAACCGGTCGCGGAGGCTATGGATGCGATCGCTGCGG

CCGATCTTAGCCAGACGAGCGGGTTCGGCCCATTCGGACCGCAAGGAATCGGTCAATACACTACATGGCG

TGATTTCATATGCGCGATTGCTGATCCCCATGTGTATCACTGGCAAACTGTGATGGACGACACCGTCAGT

GCGTCCGTCGCGCAGGCTCTCGATGAGCTGATGCTTTGGGCCGAGGACTGCCCCGAAGTCCGGCACCTCG

TGCACGCGGATTTCGGCTCCAACAATGTCCTGACGGACAATGGCCGCATAACAGCGGTCATTGACTGGAG

CGAGGCGATGTTCGGGGATTCCCAATACGAGGTCGCCAACATCTTCTTCTGGAGGCCGTGGTTGGCTTGT

ATGGAGCAGCAGACGCGCTACTTCGAGCGGAGGCATCCGGAGCTTGCAGGATCGCCACGACTCCGGGCGT

ATATGCTCCGCATTGGTCTTGACCAACTCTATCAGAGCTTGGTTGACGGCAATTTCGATGATGCAGCTTG
GGCGCAGGGTCGATGCGACGCAATCGTCCGATCCGGAGCCGGGACTGTCGGGCGTACACAAATCGCCCGC
AGAAGCGCGGCCGTCTGGACCGATGGCTGTGTAGAAGTACTCGCCGATAGTGGAAACCGACGCCCCAGCA
CTCGTCCGAGGGCAAAGAAATAGAGTAGATGCCGACCGGATCTGTCGATCGACAAGCTCGAGtttctcca
taataatgtgtgagtagttcccagataagggaattagggttcctatagggtttcgctcatgtgttgagca
tataagaaacccttagtatgtatttgtatttgtaaaatacttctatcaataaaatttctaattcctaaaa
ccaaaatccagtactaaaatccagatcCCCCGAATTAATTCGGCGTTAATTCAGTACATTAAAAACGTCC
GCAATGTGTTATTAAGTTGTCTAAGCGTCAATTTGTTTACACCACAATATATCCTGCCA

brown/lowercase: kanamycin resistance gene

CYAN/UPPERCASE/UNDERLINED: C->A transversion to block vector's BsaI site

cyan/lowercase: T-DNA right border

GREEN/UPPERCASE: 2x35S CaMV promoter

ORANGE/UPPERCASE: attB1

BLUE/UPPERCASE: *AtTAS1c* 5' region

RED/UPPERCASE: *Bsa*I site

magenta/lowercase: chloramphenicol resistance gene

MAGENTA/UPPERCASE: *ccd*B gene

red/lowercase: inverted *Bsa*I site

blue/lowercase: *AtTAS1c* 3' region

orange/lowercase: *AtMIR173*

ORANGE/UPPERCASE/UNDERLINED: attB2

GREY/UPPERCASE/UNDERLINED: Nos terminator

green/lowercase: CaMV promoter

BROWN/UPPERCASE: hygromycin resistance gene

green/lowercase/underlined: CaMV terminator

CYAN/UPPERCASE: T-DNA left border
